# Supplementary material for: Using genomics to explore the epidemiology of vancomycin resistance in a sewage system
Source: Microbiol Spectr. 2024 Dec 10;13(1):e01489-24. doi: 10.1128/spectrum.01489-24 (PMC11705837; doi:10.1128/spectrum.01489-24)
Supplement: Supplemental material — Figure S1; Tables S1 to S15. [file spectrum.01489-24-s0001.docx]

**Supplementary material**

**Supplementary table 1**: Summary table of the 181 sequenced metagenomic sewage samples from Lynetten. The table contains the fragments per sample (fragCount), the number of fragments aligned per sample to one of the 989 PanRes glycopeptide reference genes from either ResFinder or ResfinderFG (Glyco fragCountAln), the percentage of aligned fragments out of the total number of fragmentsCount for that given sample (Glyco percAln (%)) as well as the number of aligned fragments to the entire PanRes database (fragCountAln) and the percentage of aligned fragments out of the total fragmentCount for that sample (percAln %). The table also contains the metadata for Lynetten samples (sampling date, sequencing flowcell and ENA run accession).

| **Sample** | **fragCount** | **Glyco fragCountAln** | **Glyco percAln (%)** | **fragCountAln** | **percAln (%)** | **Sampling date** | **Sequencing flowcell** | **ENA run accession** |
| --- | --- | --- | --- | --- | --- | --- | --- | --- |
| DTU_2020_1007249_1_MG_RL_CPH_Sewage_710_S135_L003_R1_001 | 55606129 | 2169 | 4.00E-05 | 102819 | 0.00185 | 2020-06-29 | Novaseq | ERR12510759 |
| DTU2017-195-PRJ1066-RL-Sewage-194_S2_L001_R1_001 | 3548260 | 212 | 6.00E-05 | 5581 | 0.00157 | 2017-12-01 | MiSeq214 | ERR3562989 |
| DTU_2020_1007192_1_MG_RL_CPH_Sewage_617_S74_L002_R1_001 | 53963827 | 4034 | 7.00E-05 | 85573 | 0.00159 | 2020-07-01 | Novaseq | ERR12510686 |
| DTU_2020_1007195_1_MG_RL_CPH_Sewage_620_S32_L001_R1_001 | 47460854 | 3406 | 7.00E-05 | 72191 | 0.00152 | 2020-01-14 | Novaseq | ERR12510690 |
| DTU2018-1622-PRJ1066-RL-CPH-Sewage-341_R1_001 | 22607888 | 1744 | 8.00E-05 | 38276 | 0.00169 | 2018-02-01 | Nextseq060 | ERR3563070 |
| DTU2016-1501-PRJ1066-RL-Sewage-92_S14_L001_R1_001 | 507823 | 29 | 6.00E-05 | 774 | 0.00152 | 2016-05-28 | Miseq190 | ERR3562882 |
| DTU2018-1600-PRJ1066-RL-CPH-Sewage-402_R1_001 | 23414053 | 1989 | 8.00E-05 | 44070 | 0.00188 | 2018-12-05 | Nextseq054 | ERR3563096 |
| DTU2016-1654-PRJ1066-RL-Sewage-117_S9_L001_R1_001 | 1842527 | 102 | 6.00E-05 | 3035 | 0.00165 | 2016-07-17 | MiSeq 197 | ERR3562911 |
| DTU2017-27-PRJ1066-RL-Sewage-185_S2_L001_R1_001 | 3242853 | 188 | 6.00E-05 | 5741 | 0.00177 | 2016-12-19 | MiSeq209 | ERR3562980 |
| DTU2017-1296-PRJ1066-CPH-Sewage-34_R1_001 | 22621317 | 1894 | 8.00E-05 | 35386 | 0.00156 | 2015-11-25 | Nextseq_037 | ERR3562862 |
| DTU_2020_1007216_2_MG_RL_CPH_Sewage_642_S101_L003_R1_001 | 14808210 | 1351 | 9.00E-05 | 24787 | 0.00167 | 2020-05-03 | Novaseq | ERR12510714 |
| DTU_2020_1007158_1_MG_RL_CPH_Sewage_518_S19_L001_R1_001 | 50975741 | 3031 | 6.00E-05 | 81322 | 0.0016 | 2019-03-27 | Novaseq | ERR12510645 |
| DTU_2020_1007236_1_MG_RL_CPH_Sewage_683_S127_L004_R1_001 | 2839140 | 144 | 5.00E-05 | 5189 | 0.00183 | 2020-05-28 | Novaseq | ERR12510741 |
| DTU2016-1766-PRJ1066-Metagenome-RL-48_S8_L001_R1_001 | 829897 | 51 | 6.00E-05 | 1340 | 0.00161 | 2016-08-02 | MiSeq199 | ERR3562874 |
| DTU_2020_1007209_1_MG_RL_CPH_Sewage_634_S81_L002_R1_001 | 56385683 | 4519 | 8.00E-05 | 94237 | 0.00167 | 2020-02-19 | Novaseq | ERR12510704 |
| DTU2017-1104-PRJ1066-CPH-Sewage-130_R1_001 | 27503047 | 3626 | 0.00013 | 55807 | 0.00203 | 2016-08-18 | Nextseq_003 | ERR3562923 |
| DTU2017-1125-PRJ1066-CPH-Sewage-151_R1_001 | 24353510 | 1878 | 8.00E-05 | 54629 | 0.00224 | 2016-06-10 | NextSeq_007 | ERR3562946 |
| DTU_2020_1007282_1_MG_RL_CPH_Sewage_2_677_S110_L003_R1_001 | 44568271 | 3236 | 7.00E-05 | 80879 | 0.00181 | 2020-05-20 | Novaseq | ERR12510800 |
| DTU2017-200-PRJ1066-RL-Sewage-199_S7_L001_R1_001 | 4327193 | 93 | 2.00E-05 | 8780 | 0.00203 | 2017-01-24 | MiSeq214 | ERR3562994 |
| DTU_2020_1007176_2_MG_RL_CPH_Sewage_597_S94_L003_R1_001 | 19677704 | 1284 | 7.00E-05 | 33266 | 0.00169 | 2019-02-11 | Novaseq | ERR12510658 |
| DTU2016-1136-PRJ1066-RL-Sewage-64_S4_L001_R1_001 | 1627353 | 83 | 5.00E-05 | 3055 | 0.00188 | 2016-04-13 | Miseq185 | ERR1467144 |
| DTU_2020_1007281_1_MG_RL_CPH_Sewage_1_677_S109_L003_R1_001 | 40667393 | 3052 | 8.00E-05 | 73941 | 0.00182 | 2020-05-20 | Novaseq | ERR12510798 |
| DTU2018-1624-PRJ1066-RL-CPH-Sewage-430_R1_001 | 22487886 | 1330 | 6.00E-05 | 48360 | 0.00215 | 2018-07-08 | Nextseq060 | ERR3563100 |
| DTU2017-290-PRJ1066-RL-Sewage-201_S2_L001_R1_001 | 4291247 | 322 | 8.00E-05 | 6801 | 0.00158 | 2017-01-27 | Miseq217 | ERR3562996 |
| DTU_2020_1007258_1_MG_RL_CPH_Sewage_719_S118_L003_R1_001 | 51061134 | 2770 | 5.00E-05 | 95045 | 0.00186 | 2020-07-23 | Novaseq | ERR12510768 |
| 71_RL_R1_001 | 27447345 | 1778 | 6.00E-05 | 39803 | 0.00145 | 2016-03-02 | HiSeq | ERR2607438 |
| DTU2017-1110-PRJ1066-CPH-Sewage-136_R1_001 | 30322974 | 1392 | 5.00E-05 | 71142 | 0.00235 | 2016-08-29 | Nextseq_003 | ERR3562929 |
| DTU2017-1139-PRJ1066-CPH-Sewage-165_R1_001 | 61471865 | 2780 | 5.00E-05 | 103999 | 0.00169 | 2016-11-13 | NextSeq_008 | ERR3562959 |
| DTU_2020_1007200_1_MG_RL_CPH_Sewage_625_S78_L002_R1_001 | 51818370 | 2599 | 5.00E-05 | 90799 | 0.00175 | 2020-02-29 | Novaseq | ERR12510695 |
| DTU2016-1163-PRJ1066-RL-Sewage-61_S5_L001_R1_001 | 2234070 | 88 | 4.00E-05 | 2834 | 0.00127 | 2016-03-29 | Miseq188 | ERR1470826 |
| DTU_2020_1007186_1_MG_RL_CPH_Sewage_608_S136_L004_R1_001 | 2423526 | 163 | 7.00E-05 | 4641 | 0.00191 | 2019-02-12 | Novaseq | ERR12510676 |
| DTU_2020_1007183_2_MG_RL_CPH_Sewage_605_S5_L001_R1_001 | 39732951 | 2961 | 7.00E-05 | 77591 | 0.00195 | 2019-11-24 | Novaseq | ERR12510671 |
| DTU2016-1137-PRJ1066-RL-Sewage-65_S5_L001_R1_001 | 2171942 | 181 | 8.00E-05 | 3783 | 0.00174 | 2016-04-17 | Miseq185 | ERR1467145 |
| DTU2016-1162-PRJ1066-RL-Sewage-60_S4_L001_R1_001 | 2153868 | 92 | 4.00E-05 | 3317 | 0.00154 | 2016-03-20 | Miseq188 | ERR1470825 |
| DTU2018-101-PRJ1066-CPH-Sewage-227_R1_001 | 21171594 | 2337 | 0.00011 | 32432 | 0.00153 | 2017-03-27 | Nextseq_015 | ERR3563024 |
| DTU2017-1300-PRJ1066-CPH-Sewage-38_R1_001 | 37995654 | 2200 | 6.00E-05 | 67069 | 0.00177 | 2015-12-17 | Nextseq_042 | ERR3562866 |
| DTU_2020_1007270_2_MG_RL_CPH_Sewage_734_S98_L003_R1_001 | 16271427 | 852 | 5.00E-05 | 27968 | 0.00172 | 2020-08-19 | Novaseq | ERR12510782 |
| DTU_2020_1007264_1_MG_RL_CPH_Sewage_725_S138_L003_R1_001 | 55761739 | 2447 | 4.00E-05 | 101673 | 0.00182 | 2020-04-08 | Novaseq | ERR12510774 |
| DTU_2020_1007267_2_MG_RL_CPH_Sewage_731_S145_L004_R1_001 | 8507699 | 495 | 6.00E-05 | 16253 | 0.00191 | 2020-12-08 | Novaseq | ERR12510778 |
| DTU2018-1623-PRJ1066-RL-CPH-Sewage-372_R1_001 | 27943298 | 1243 | 4.00E-05 | 47610 | 0.0017 | 2018-06-03 | Nextseq060 | ERR3563080 |
| DTU_2020_1007242_2_MG_RL_CPH_Sewage_695_S61_L002_R1_001 | 39356162 | 2449 | 6.00E-05 | 77119 | 0.00196 | 2020-10-06 | Novaseq | ERR12510752 |
| DTU2016-1652-PRJ1066-RL-Sewage-115_S7_L001_R1_001 | 1544369 | 63 | 4.00E-05 | 2540 | 0.00164 | 2016-07-13 | MiSeq 197 | ERR3562909 |
| DTU2016-1493-PRJ1066-RL-Sewage-56_S6_L001_R1_001 | 1889678 | 76 | 4.00E-05 | 2914 | 0.00154 | 2016-01-03 | Miseq190 | ERR1512996 |
| DTU_2020_1007230_2_MG_RL_CPH_Sewage_662_S421_L004_R1_001 | 40067070 | 2416 | 6.00E-05 | 74639 | 0.00186 | 2020-05-05 | Novaseq | ERR12510733 |
| DTU2018-1619-PRJ1066-RL-CPH-ReSewage-234_R1_001 | 23607641 | 1286 | 5.00E-05 | 62765 | 0.00266 | 2017-11-04 | Nextseq058 | ERR3563030 |
| DTU2017-1119-PRJ1066-CPH-Sewage-145_R1_001 | 31800759 | 1721 | 5.00E-05 | 50447 | 0.00159 | 2016-09-21 | NextSeq_005 | ERR3562940 |
| DTU2019-195-PRJ1066-RL-CPH-Sewage-219_R1_001 | 23482357 | 1544 | 7.00E-05 | 33325 | 0.00142 | 2017-02-22 | NextSeq_081 | ERR3563015 |
| DTU2017-1135-PRJ1066-CPH-Sewage-161_R1_001 | 24418485 | 1516 | 6.00E-05 | 30678 | 0.00126 | 2016-10-23 | NextSeq_006 | ERR3562955 |
| DTU2017-585-PRJ1066-RL-Sewage-221_S4_L001_R1_001 | 7173887 | 669 | 9.00E-05 | 11240 | 0.00157 | 2017-05-03 | Miseq232 | ERR3563017 |
| DTU_2023_1016969_1_MG_RL_CPH_Sewage_1025_R1_001 | 22695643 | 1586 | 7.00E-05 | 30359 | 0.00134 | 2022-10-25 | Nextseq | ERR13597803 |
| DTU2017-583-PRJ1066-RL-Sewage-219_S2_L001_R1_001 | 1935509 | 121 | 6.00E-05 | 2315 | 0.0012 | 2017-02-22 | Miseq232 | ERR3563014 |
| DTU_2020_1007180_1_MG_RL_CPH_Sewage_602_S148_L004_R1_001 | 21542799 | 856 | 4.00E-05 | 31642 | 0.00147 | 2019-11-17 | Novaseq | ERR12510664 |
| DTU_2020_1007198_1_MG_RL_CPH_Sewage_623_S76_L002_R1_001 | 49927942 | 2101 | 4.00E-05 | 95823 | 0.00192 | 2020-01-22 | Novaseq | ERR12510693 |
| DTU2019-199-PRJ1066-RL-CPH-Sewage-227_R1_001 | 21506613 | 2110 | 0.0001 | 31848 | 0.00148 | 2017-03-27 | NextSeq_081 | ERR3563025 |
| DTU2016-1773-PRJ1066-Metagenome-RL-45_S15_L001_R1_001 | 648355 | 40 | 6.00E-05 | 995 | 0.00153 | 2016-01-17 | MiSeq199 | ERR3562872 |
| DTU_2020_1007275_2_MG_RL_CPH_Sewage_1_586_S150_L004_R1_001 | 9264370 | 293 | 3.00E-05 | 17739 | 0.00191 | 2020-09-28 | Novaseq | ERR12510791 |
| DTU2016-1606-PRJ1066-RL-Sewage-102_S6_L001_R1_001 | 611142 | 26 | 4.00E-05 | 877 | 0.00144 | 2016-06-14 | Miseq195 | ERR3562897 |
| DTU2016-1144-PRJ1066-RL-Sewage-78_S12_L001_R1_001 | 1906186 | 102 | 5.00E-05 | 3438 | 0.0018 | 2016-02-04 | Miseq185 | ERR1467152 |
| DTU2019-224-PRJ1066-RL-CPH-Sewage-380_R1_001 | 26049139 | 1922 | 7.00E-05 | 34418 | 0.00132 | 2018-03-20 | Nextseq_085 | ERR3563088 |
| DTU2016-1161-PRJ1066-RL-Sewage-59_S3_L001_R1_001 | 2078154 | 113 | 5.00E-05 | 3086 | 0.00148 | 2016-03-15 | Miseq188 | ERR1470824 |
| DTU_2020_1007216_1_MG_RL_CPH_Sewage_642_S10_L001_R1_001 | 33543462 | 2988 | 9.00E-05 | 57372 | 0.00171 | 2020-05-03 | Novaseq | ERR12510713 |
| DTU2017-1299-PRJ1066-CPH-Sewage-37_R1_001 | 33353283 | 2347 | 7.00E-05 | 46510 | 0.00139 | 2015-12-15 | Nextseq_042 | ERR3562865 |
| DTU2017-1128-PRJ1066-CPH-Sewage-154_R1_001 | 34786429 | 1719 | 5.00E-05 | 51147 | 0.00147 | 2016-09-10 | NextSeq_006 | ERR3562949 |
| DTU2017-190-PRJ1066-RL-Sewage-190_S3_L001_R1_001 | 5135117 | 353 | 7.00E-05 | 7067 | 0.00138 | 2017-04-01 | MiSeq213 | ERR3562985 |
| DTU2018-100-PRJ1066-RL-CPH-Sewage-252_R1_001 | 18115460 | 921 | 5.00E-05 | 27462 | 0.00152 | 2017-09-05 | Nextseq054 | ERR3563045 |
| DTU_2020_1007276_1_MG_RL_CPH_Sewage_2_586_S106_L003_R1_001 | 44080934 | 1372 | 3.00E-05 | 86011 | 0.00195 | 2020-09-28 | Novaseq | ERR12510792 |
| DTU2018-96-PRJ1066-RL-CPH-Sewage-239_R1_001 | 20535350 | 1069 | 5.00E-05 | 54775 | 0.00267 | 2017-05-15 | Nextseq054 | ERR3563035 |
| DTU_2020_1007215_2_MG_RL_CPH_Sewage_640_S41_L001_R1_001 | 38429277 | 2019 | 5.00E-05 | 65026 | 0.00169 | 2020-02-04 | Novaseq | ERR12510712 |
| DTU2018-1621-PRJ1066-RL-CPH-Sewage-298_R1_001 | 26930966 | 1034 | 4.00E-05 | 40800 | 0.00151 | 2017-09-13 | Nextseq060 | ERR3563059 |
| DTU2016-1604-PRJ1066-RL-Sewage-100_S4_L001_R1_001 | 682435 | 38 | 6.00E-05 | 1035 | 0.00152 | 2016-12-06 | Miseq195 | ERR3562893 |
| DTU2016-1663-PRJ1066-RL-Sewage-98_S18_L001_R1_001 | 1458070 | 57 | 4.00E-05 | 2341 | 0.00161 | 2016-08-06 | MiSeq 197 | ERR3562888 |
| DTU2016-1536-PRJ1066-RL-Sewage-90_S3_L001_R1_001 | 2196280 | 89 | 4.00E-05 | 4028 | 0.00183 | 2016-05-25 | Miseq193 | ERR1513004 |
| DTU2018-1598-PRJ1066-RL-CPH-Sewage-319_R1_001 | 19636598 | 1914 | 0.0001 | 38267 | 0.00195 | 2017-04-11 | Nextseq054 | ERR3563067 |
| DTU2018-1597-PRJ1066-RL-CPH-Sewage-316_R1_001 | 16255120 | 1557 | 0.0001 | 30386 | 0.00187 | 2017-10-30 | Nextseq054 | ERR3563064 |
| DTU2016-1534-PRJ1066-RL-Sewage-88_S1_L001_R1_001 | 2395472 | 104 | 4.00E-05 | 3410 | 0.00142 | 2016-05-20 | Miseq193 | ERR1513002 |
| DTU2017-23-PRJ1066-RL-Sewage-181_S4_L001_R1_001 | 122618 | 6 | 5.00E-05 | 262 | 0.00214 | 2016-12-12 | MiSeq210 | ERR3562975 |
| DTU2016-1134-PRJ1066-RL-Sewage-62_S2_L001_R1_001 | 1457568 | 89 | 6.00E-05 | 2428 | 0.00167 | 2016-11-04 | Miseq185 | ERR1467142 |
| DTU2016-1538-PRJ1066-RL-Sewage-92_S5_L001_R1_001 | 2006168 | 95 | 5.00E-05 | 3423 | 0.00171 | 2016-05-28 | Miseq193 | ERR1513006 |
| DTU2016-1496-PRJ1066-RL-Sewage-87_S9_L001_R1_001 | 1860751 | 61 | 3.00E-05 | 3712 | 0.00199 | 2016-05-18 | Miseq190 | ERR1512998 |
| DTU2017-584-PRJ1066-RL-Sewage-220_S3_L001_R1_001 | 3350863 | 308 | 9.00E-05 | 4961 | 0.00148 | 2017-02-03 | Miseq232 | ERR3563016 |
| DTU2016-1650-PRJ1066-RL-Sewage-113_S5_L001_R1_001 | 1792393 | 44 | 2.00E-05 | 3490 | 0.00195 | 2016-02-07 | MiSeq 197 | ERR3562907 |
| DTU2016-1617-PRJ1066-RL-Sewage-49_S16_L001_R1_001 | 1455986 | 104 | 7.00E-05 | 2029 | 0.00139 | 2016-12-02 | Miseq 195 | ERR3562875 |
| DTU2017-189-PRJ1066-RL-Sewage-181_S2_L001_R1_001 | 4285442 | 389 | 9.00E-05 | 8381 | 0.00196 | 2016-12-12 | Miseq213 | ERR3562976 |
| DTU2017-1113-PRJ1066-CPH-Sewage-139_R1_001 | 4570349 | 264 | 6.00E-05 | 7279 | 0.00159 | 2016-07-09 | NextSeq_004 | ERR3562933 |
| DTU_2020_1007255_1_MG_RL_CPH_Sewage_716_S116_L003_R1_001 | 50148939 | 2631 | 5.00E-05 | 96241 | 0.00192 | 2020-07-14 | Novaseq | ERR12510765 |
| DTU2019-219-PRJ1066-RL-CPH-Sewage-375_R1_001 | 30929742 | 2635 | 9.00E-05 | 49463 | 0.0016 | 2018-12-03 | Nextseq_085 | ERR3563083 |
| DTU2017-192-PRJ1066-RL-Sewage-192_S5_L001_R1_001 | 6426638 | 316 | 5.00E-05 | 11350 | 0.00177 | 2017-08-01 | MiSeq213 | ERR3562987 |
| DTU_2020_1007189_1_MG_RL_CPH_Sewage_614_S73_L002_R1_001 | 61130632 | 4308 | 7.00E-05 | 99586 | 0.00163 | 2019-12-20 | Novaseq | ERR12510682 |
| DTU_2023_1016974_1_MG_RL_CPH_Sewage_1051_R1_001 | 10512441 | 800 | 8.00E-05 | 15713 | 0.00149 | 2023-01-15 | Nextseq | ERR13597804 |
| DTU2016-1172-PRJ1066-RL-Sewage-85_S14_L001_R1_001 | 90752 | 5 | 6.00E-05 | 152 | 0.00167 | 2016-05-16 | Miseq188 | ERR3562877 |
| DTU2019-216-PRJ1066-RL-CPH-Sewage-369_R1_001 | 18688244 | 1169 | 6.00E-05 | 35574 | 0.0019 | 2018-01-03 | Nextseq_084 | ERR3563077 |
| DTU2017-1130-PRJ1066-CPH-Sewage-156_R1_001 | 38185727 | 2737 | 7.00E-05 | 63056 | 0.00165 | 2016-12-10 | NextSeq_007 | ERR3562951 |
| DTU2017-1145-PRJ1066-CPH-Sewage-171_R1_001 | 5196258 | 255 | 5.00E-05 | 12982 | 0.0025 | 2016-09-11 | NextSeq_009 | ERR3562965 |
| DTU2018-1599-PRJ1066-RL-CPH-Sewage-399_R1_001 | 20946657 | 1494 | 7.00E-05 | 37399 | 0.00179 | 2018-04-05 | Nextseq054 | ERR3563093 |
| DTU2017-1278-PRJ1066-CPH-Sewage-143_R1_001 | 7016491 | 353 | 5.00E-05 | 14089 | 0.00201 | 2016-09-18 | NextSes_010 | ERR3562938 |
| DTU2019-397-PRJ1066-RL-CPH-Sewage-285_R1_001 | 14492602 | 960 | 7.00E-05 | 34704 | 0.00239 | 2017-08-08 | Nextseq_089 | ERR3563054 |
| DTU2019-202-PRJ1066-RL-CPH-Sewage-232_R1_001 | 19460288 | 1082 | 6.00E-05 | 64753 | 0.00333 | 2017-05-22 | Nextseq_082 | ERR3563028 |
| DTU2017-1144-PRJ1066-CPH-Sewage-170_R1_001 | 5198160 | 289 | 6.00E-05 | 12002 | 0.00231 | 2016-02-11 | NextSeq_009 | ERR3562964 |
| DTU2019-214-PRJ1066-RL-CPH-Sewage-367_R1_001 | 22873953 | 2114 | 9.00E-05 | 42975 | 0.00188 | 2018-02-26 | Nextseq_084 | ERR3563075 |
| DTU_2020_1007267_1_MG_RL_CPH_Sewage_731_S124_L003_R1_001 | 37025165 | 2257 | 6.00E-05 | 70887 | 0.00191 | 2020-12-08 | Novaseq | ERR12510777 |
| DTU2017-1297-PRJ1066-CPH-Sewage-35_R1_001 | 24437045 | 1663 | 7.00E-05 | 36026 | 0.00147 | 2015-01-12 | Nextseq_037 | ERR3562863 |
| DTU2017-1106-PRJ1066-CPH-Sewage-132_R1_001 | 4664614 | 235 | 5.00E-05 | 8636 | 0.00185 | 2016-08-22 | NextSeq_004 | ERR3562925 |
| DTU2017-1141-PRJ1066-CPH-Sewage-167_R1_001 | 28799693 | 1659 | 6.00E-05 | 48612 | 0.00169 | 2016-11-17 | NextSeq_009 | ERR3562961 |
| DTU2017-297-PRJ1066-RL-Sewage-208_S3_L001_R1_001 | 5969925 | 403 | 7.00E-05 | 9869 | 0.00165 | 2017-11-02 | MiSeq218 | ERR3563003 |
| DTU2016-1656-PRJ1066-RL-Sewage-119_S11_L001_R1_001 | 1858855 | 113 | 6.00E-05 | 3179 | 0.00171 | 2016-07-20 | MiSeq 197 | ERR3562913 |
| DTU2017-1148-PRJ1066-CPH-Sewage-174_R1_001 | 14261075 | 1224 | 9.00E-05 | 27070 | 0.0019 | 2016-11-26 | NextSeq_008 | ERR3562968 |
| DTU_2020_1007206_1_MG_RL_CPH_Sewage_631_S36_L001_R1_001 | 46727361 | 1500 | 3.00E-05 | 31700 | 0.00068 | 2020-11-02 | Novaseq | ERR12510701 |
| DTU2019-200-PRJ1066-RL-CPH-Sewage-228_R1_001 | 15166044 | 1646 | 0.00011 | 25731 | 0.0017 | 2017-10-03 | Nextseq_081 | ERR3563026 |
| DTU_2020_1007180_2_MG_RL_CPH_Sewage_602_S60_L002_R1_001 | 30129899 | 1223 | 4.00E-05 | 43964 | 0.00146 | 2019-11-17 | Novaseq | ERR12510665 |
| DTU2017-1302-PRJ1066-CPH-Sewage-40_R1_001 | 29281853 | 2069 | 7.00E-05 | 47162 | 0.00161 | 2015-12-28 | Nextseq_042 | ERR3562868 |
| DTU_2020_1007245_1_MG_RL_CPH_Sewage_701_S84_L002_R1_001 | 59456446 | 3353 | 6.00E-05 | 112456 | 0.00189 | 2020-06-17 | Novaseq | ERR12510755 |
| DTU2017-295-PRJ1066-RL-Sewage-206_S1_L001_R1_001 | 592386 | 54 | 9.00E-05 | 956 | 0.00161 | 2017-02-02 | MiSeq218 | ERR3563001 |
| DTU2017-1122-PRJ1066-CPH-Sewage-148_R1_001 | 35218943 | 1819 | 5.00E-05 | 55297 | 0.00157 | 2016-09-24 | NextSeq_006 | ERR3562943 |
| DTU2016-1495-PRJ1066-RL-Sewage-85_S8_L001_R1_001 | 1637611 | 94 | 6.00E-05 | 2588 | 0.00158 | 2016-05-16 | Miseq190 | ERR1512990 |
| DTU2017-1303-PRJ1066-CPH-Sewage-41_R1_001 | 32048014 | 1964 | 6.00E-05 | 50432 | 0.00157 | 2015-12-29 | Nextseq_042 | ERR3562869 |
| DTU2016-1169-PRJ1066-RL-Sewage-82_S11_L001_R1_001 | 1870858 | 81 | 4.00E-05 | 2817 | 0.00151 | 2016-10-05 | Miseq188 | ERR1512985 |
| DTU_2020_1007224_1_MG_RL_CPH_Sewage_651_S45_L001_R1_001 | 53643387 | 4254 | 8.00E-05 | 102207 | 0.00191 | 2020-03-26 | Novaseq | ERR12510726 |
| DTU_2020_1007222_1_MG_RL_CPH_Sewage_649_S13_L001_R1_001 | 43966749 | 3056 | 7.00E-05 | 70838 | 0.00161 | 2020-03-20 | Novaseq | ERR12510723 |
| DTU_2020_1007203_1_MG_RL_CPH_Sewage_628_S35_L001_R1_001 | 48402495 | 2083 | 4.00E-05 | 71378 | 0.00147 | 2020-04-02 | Novaseq | ERR12510698 |
| DTU_2020_1007275_1_MG_RL_CPH_Sewage_1_586_S105_L003_R1_001 | 37341232 | 1218 | 3.00E-05 | 73112 | 0.00196 | 2020-09-28 | Novaseq | ERR12510790 |
| DTU2016-1497-PRJ1066-RL-Sewage-88_S10_L001_R1_001 | 62315 | 2 | 3.00E-05 | 47 | 0.00075 | 2016-05-20 | Miseq190 | ERR3562878 |
| DTU2016-1647-PRJ1066-RL-Sewage-110_S2_L001_R1_001 | 1780779 | 69 | 4.00E-05 | 3004 | 0.00169 | 2016-04-07 | MiSeq 197 | ERR3562904 |
| DTU_2020_1007236_2_MG_RL_CPH_Sewage_683_S66_L002_R1_001 | 41384795 | 2571 | 6.00E-05 | 79465 | 0.00192 | 2020-05-28 | Novaseq | ERR12510742 |
| DTU2017-1101-PRJ1066-CPH-Sewage-127_R1_001 | 25047918 | 2364 | 9.00E-05 | 85519 | 0.00341 | 2016-10-08 | NextSeq_002 | ERR3562920 |
| DTU_2020_1007215_1_MG_RL_CPH_Sewage_640_S139_L004_R1_001 | 8199636 | 387 | 5.00E-05 | 13394 | 0.00163 | 2020-02-04 | Novaseq | ERR12510711 |
| DTU2017-1295-PRJ1066-CPH-Sewage-33_R1_001 | 42237549 | 3014 | 7.00E-05 | 63390 | 0.0015 | 2015-11-20 | Nextseq_037 | ERR3562861 |
| DTU2016-1612-PRJ1066-RL-Sewage-107_S11_L001_R1_001 | 937066 | 41 | 4.00E-05 | 2690 | 0.00287 | 2016-06-27 | Miseq 195 | ERR3562901 |
| DTU2016-1145-PRJ1066-RL-Sewage-79_S13_L001_R1_001 | 1230636 | 83 | 7.00E-05 | 2370 | 0.00193 | 2016-04-29 | Miseq185 | ERR1467153 |
| DTU_2020_1007239_1_MG_RL_CPH_Sewage_689_S53_L001_R1_001 | 45568944 | 3364 | 7.00E-05 | 74289 | 0.00163 | 2020-04-06 | Novaseq | ERR12510747 |
| DTU2016-1774-PRJ1066-Metagenome-RL-44_S16_L001_R1_001 | 633837 | 40 | 6.00E-05 | 930 | 0.00147 | 2016-11-01 | MiSeq199 | ERR3562871 |
| DTU2016-1658-PRJ1066-RL-Sewage-121_S13_L001_R1_001 | 1850830 | 99 | 5.00E-05 | 2708 | 0.00146 | 2016-07-22 | MiSeq 197 | ERR3562915 |
| DTU2017-1116-PRJ1066-CPH-Sewage-142_R1_001 | 40953812 | 2079 | 5.00E-05 | 70829 | 0.00173 | 2016-09-13 | NextSeq_002 | ERR3562937 |
| DTU_2020_1007230_1_MG_RL_CPH_Sewage_662_S142_L004_R1_001 | 46781 | 3 | 6.00E-05 | 105 | 0.00224 | 2020-05-05 | Novaseq | ERR12510732 |
| DTU_2020_1007272_1_MG_RL_CPH_Sewage_740_S127_L003_R1_001 | 40103698 | 2213 | 6.00E-05 | 77796 | 0.00194 | 2020-08-27 | Novaseq | ERR12510785 |
| DTU2016-1570-PRJ1066-RL-Sewage-95_S11_L001_R1_001 | 2537853 | 137 | 5.00E-05 | 3407 | 0.00134 | 2016-01-06 | Miseq193 | ERR1514428 |
| DTU_2020_1007252_1_MG_RL_CPH_Sewage_713_S113_L003_R1_001 | 43513069 | 2248 | 5.00E-05 | 79564 | 0.00183 | 2020-07-07 | Novaseq | ERR12510762 |
| DTU2016-1159-PRJ1066-RL-Sewage-57_S1_L001_R1_001 | 2214998 | 189 | 9.00E-05 | 3129 | 0.00141 | 2016-10-03 | Miseq188 | ERR1470822 |
| DTU2016-1767-PRJ1066-Metagenome-RL-46_S9_L001_R1_001 | 687674 | 52 | 8.00E-05 | 1031 | 0.0015 | 2016-01-28 | MiSeq199 | ERR3562873 |
| DTU_2020_1007242_1_MG_RL_CPH_Sewage_695_S144_L004_R1_001 | 7985901 | 434 | 5.00E-05 | 15791 | 0.00198 | 2020-10-06 | Novaseq | ERR12510751 |
| DTU_2020_1007261_1_MG_RL_CPH_Sewage_722_S120_L003_R1_001 | 55453193 | 2798 | 5.00E-05 | 118201 | 0.00213 | 2020-07-29 | Novaseq | ERR12510771 |
| DTU2017-1301-PRJ1066-CPH-Sewage-39_R1_001 | 35052863 | 2533 | 7.00E-05 | 58897 | 0.00168 | 2015-12-21 | Nextseq_042 | ERR3562867 |
| DTU2017-1274-PRJ1066-CPH-Sewage-129_R1_001 | 32657986 | 4259 | 0.00013 | 62171 | 0.0019 | 2016-08-16 | NextSeq_011 | ERR3562922 |
| DTU_2020_1007230_3_MG_RL_CPH_Sewage_662_S70_L002_R1_001 | 35759556 | 2217 | 6.00E-05 | 66385 | 0.00186 | 2020-05-05 | Novaseq | ERR12510734 |
| DTU2017-299-PRJ1066-RL-Sewage-210_S5_L001_R1_001 | 7209143 | 509 | 7.00E-05 | 11462 | 0.00159 | 2017-02-13 | MiSeq218 | ERR3563005 |
| DTU_2020_1007226_1_MG_RL_CPH_Sewage_653_S47_L001_R1_001 | 64473542 | 4811 | 7.00E-05 | 119055 | 0.00185 | 2020-04-16 | Novaseq | ERR12510728 |
| DTU2016-1611-PRJ1066-RL-Sewage-106_S10_L001_R1_001 | 2036344 | 76 | 4.00E-05 | 3583 | 0.00176 | 2016-06-18 | Miseq 195 | ERR3562900 |
| DTU_2020_1007219_1_MG_RL_CPH_Sewage_646_S11_L001_R1_001 | 27588898 | 2374 | 9.00E-05 | 44970 | 0.00163 | 2020-12-03 | Novaseq | ERR12510718 |
| DTU2017-29-PRJ1066-RL-Sewage-187_S4_L001_R1_001 | 2964643 | 172 | 6.00E-05 | 4105 | 0.00138 | 2016-12-22 | MiSeq209 | ERR3562982 |
| DTU2016-1489-PRJ1066-RL-Sewage-51_S2_L001_R1_001 | 1494361 | 70 | 5.00E-05 | 2133 | 0.00143 | 2016-02-20 | Miseq190 | ERR1512992 |
| DTU_2020_1007186_2_MG_RL_CPH_Sewage_608_S27_L001_R1_001 | 41209180 | 2981 | 7.00E-05 | 80776 | 0.00196 | 2019-02-12 | Novaseq | ERR12510677 |
| DTU_2023_1016977_1_MG_RL_CPH_Sewage_1042_R1_001 | 36865186 | 2323 | 6.00E-05 | 48922 | 0.00133 | 2022-09-12 | Nextseq | ERR13597805 |
| DTU2016-1146-PRJ1066-RL-Sewage-80_S14_L001_R1_001 | 949585 | 36 | 4.00E-05 | 1694 | 0.00178 | 2016-02-05 | Miseq185 | ERR1467154 |
| DTU_2020_1007176_1_MG_RL_CPH_Sewage_597_S23_L001_R1_001 | 29634364 | 1874 | 6.00E-05 | 50241 | 0.0017 | 2019-02-11 | Novaseq | ERR12510657 |
| DTU_2020_1007219_2_MG_RL_CPH_Sewage_646_S155_L004_R1_001 | 28556817 | 2593 | 9.00E-05 | 47156 | 0.00165 | 2020-12-03 | Novaseq | ERR12510719 |
| DTU2017-1298-PRJ1066-CPH-Sewage-36_R1_001 | 26999883 | 1176 | 4.00E-05 | 39984 | 0.00148 | 2015-09-12 | Nextseq_037 | ERR3562864 |
| DTU2017-1276-PRJ1066-CPH-Sewage-136_R1_001 | 15801774 | 532 | 3.00E-05 | 22646 | 0.00143 | 2016-08-29 | Nextseq_036 | ERR3562930 |
| DTU_2020_1007170_1_MG_RL_CPH_Sewage_571_S21_L001_R1_001 | 42868128 | 2683 | 6.00E-05 | 79866 | 0.00186 | 2019-08-29 | Novaseq | ERR12510651 |
| DTU2016-1615-PRJ1066-RL-Sewage-50_S14_L001_R1_001 | 1685368 | 142 | 8.00E-05 | 2479 | 0.00147 | 2016-02-18 | Miseq 195 | ERR3562876 |
| DTU_2020_1007281_2_MG_RL_CPH_Sewage_1_677_S147_L004_R1_001 | 3726940 | 243 | 7.00E-05 | 6638 | 0.00178 | 2020-05-20 | Novaseq | ERR12510799 |
| DTU_2020_1007173_1_MG_RL_CPH_Sewage_592_S64_L002_R1_001 | 43764329 | 1434 | 3.00E-05 | 79469 | 0.00182 | 2019-10-13 | Novaseq | ERR12510652 |
| DTU2017-188-PRJ1066-RL-Sewage-177_S1_L001_R1_001 | 4696430 | 231 | 5.00E-05 | 7721 | 0.00164 | 2016-07-12 | MiSeq213 | ERR3562971 |
| DTU2017-582-PRJ1066-RL-Sewage-218_S1_L001_R1_001 | 4609791 | 358 | 8.00E-05 | 7517 | 0.00163 | 2017-02-17 | Miseq232 | ERR3563013 |
| DTU2016-1135-PRJ1066-RL-Sewage-63_S3_L001_R1_001 | 2902363 | 184 | 6.00E-05 | 3788 | 0.00131 | 2016-07-04 | Miseq185 | ERR1467143 |
| DTU_2020_1007227_1_MG_RL_CPH_Sewage_654_S71_L002_R1_001 | 48766212 | 3315 | 7.00E-05 | 94714 | 0.00194 | 2020-04-27 | Novaseq | ERR12510729 |
| DTU2018-1620-PRJ1066-RL-CPH-Sewage-275_R1_001 | 20670653 | 1079 | 5.00E-05 | 51113 | 0.00247 | 2017-05-07 | Nextseq060 | ERR3563050 |
| DTU2017-1304-PRJ1066-CPH-Sewage-42_R1_001 | 32761402 | 1600 | 5.00E-05 | 40280 | 0.00123 | 2016-05-01 | Nextseq_042 | ERR3562870 |
| DTU2017-1149-PRJ1066-CPH-Sewage-175_R1_001 | 7661856 | 303 | 4.00E-05 | 12291 | 0.0016 | 2016-04-12 | NextSeq_009 | ERR3562969 |
| DTU_2020_1007233_1_MG_RL_CPH_Sewage_671_S49_L001_R1_001 | 52552768 | 3775 | 7.00E-05 | 100835 | 0.00192 | 2020-05-14 | Novaseq | ERR12510738 |
| DTU2019-209-PRJ1066-RL-CPH-Sewage-243_R1_001 | 18709689 | 461 | 2.00E-05 | 33189 | 0.00177 | 2017-04-20 | Nextseq_083 | ERR3563038 |
| DTU_2020_1007247_1_MG_RL_CPH_Sewage_707_S99_L003_R1_001 | 44309671 | 2699 | 6.00E-05 | 94973 | 0.00214 | 2020-06-24 | Novaseq | ERR12510757 |
| DTU2016-1160-PRJ1066-RL-Sewage-58_S2_L001_R1_001 | 597156 | 39 | 7.00E-05 | 933 | 0.00156 | 2016-03-13 | Miseq188 | ERR1470823 |
| DTU2016-1492-PRJ1066-RL-Sewage-55_S5_L001_R1_001 | 2016557 | 34 | 2.00E-05 | 3517 | 0.00174 | 2016-02-23 | Miseq190 | ERR1512995 |
| DTU2019-228-PRJ1066-RL-CPH-Sewage-459_R1_001 | 15129678 | 624 | 4.00E-05 | 26820 | 0.00177 | 2018-10-18 | Nextseq_086 | ERR3563110 |
| DTU_2020_1007272_2_MG_RL_CPH_Sewage_740_S146_L004_R1_001 | 4769048 | 242 | 5.00E-05 | 9073 | 0.0019 | 2020-08-27 | Novaseq | ERR12510786 |
| DTU_2020_1007183_1_MG_RL_CPH_Sewage_605_S149_L004_R1_001 | 4902340 | 327 | 7.00E-05 | 9602 | 0.00196 | 2019-11-24 | Novaseq | ERR12510670 |
| DTU_2020_1007270_1_MG_RL_CPH_Sewage_734_S126_L003_R1_001 | 34312227 | 1794 | 5.00E-05 | 60041 | 0.00175 | 2020-08-19 | Novaseq | ERR12510781 |
| DTU2019-221-PRJ1066-RL-CPH-Sewage-377_R1_001 | 17508702 | 1499 | 9.00E-05 | 24919 | 0.00142 | 2018-03-16 | Nextseq_085 | ERR3563085 |
| DTU2017-293-PRJ1066-RL-Sewage-204_S5_L001_R1_001 | 4070264 | 216 | 5.00E-05 | 5438 | 0.00134 | 2017-07-02 | Miseq217 | ERR3562999 |
| DTU2017-20-PRJ1066-RL-Sewage-178_S1_L001_R1_001 | 5426713 | 195 | 4.00E-05 | 8314 | 0.00153 | 2016-09-12 | MiSeq210 | ERR3562972 |
| DTU2017-31-PRJ1066-RL-Sewage-189_S6_L001_R1_001 | 6893619 | 475 | 7.00E-05 | 11119 | 0.00161 | 2017-01-01 | MiSeq209 | ERR3562984 |
| DTU2016-1499-PRJ1066-RL-Sewage-90_S12_L001_R1_001 | 36329 | 0 | 0 | 29 | 0.0008 | 2016-05-25 | Miseq190 | ERR3562880 |
| cph-617 | NA | NA | NA | NA | NA | NA | NovaSeq - HiC | ERR12510657 |
| cph-623 | NA | NA | NA | NA | NA | NA | NovaSeq - HiC | ERR12510693 |
| cph-625 | NA | NA | NA | NA | NA | NA | NovaSeq - HiC | ERR12510695 |
| cph-631 | NA | NA | NA | NA | NA | NA | NovaSeq - HiC | ERR12510701 |
| cph-640 | NA | NA | NA | NA | NA | NA | NovaSeq - HiC | ERR12510712 |
| cph-642 | NA | NA | NA | NA | NA | NA | NovaSeq - HiC | ERR12510714 |
| cph-649 | NA | NA | NA | NA | NA | NA | NovaSeq - HiC | ERR12510719 |
| cph-654 | NA | NA | NA | NA | NA | NA | NovaSeq - HiC | ERR12510729 |
| cph-662 | NA | NA | NA | NA | NA | NA | NovaSeq - HiC | ERR12510733 |
| cph-683 | NA | NA | NA | NA | NA | NA | NovaSeq - HiC | ERR12510741 |
| cph-689 | NA | NA | NA | NA | NA | NA | NovaSeq - HiC | ERR12510747 |
| cph-710 | NA | NA | NA | NA | NA | NA | NovaSeq - HiC | ERR12510759 |

**Supplementary figure 1:** Glycopeptide resistance in Rensningsanlæg Lynetten is compositionally stable in time.


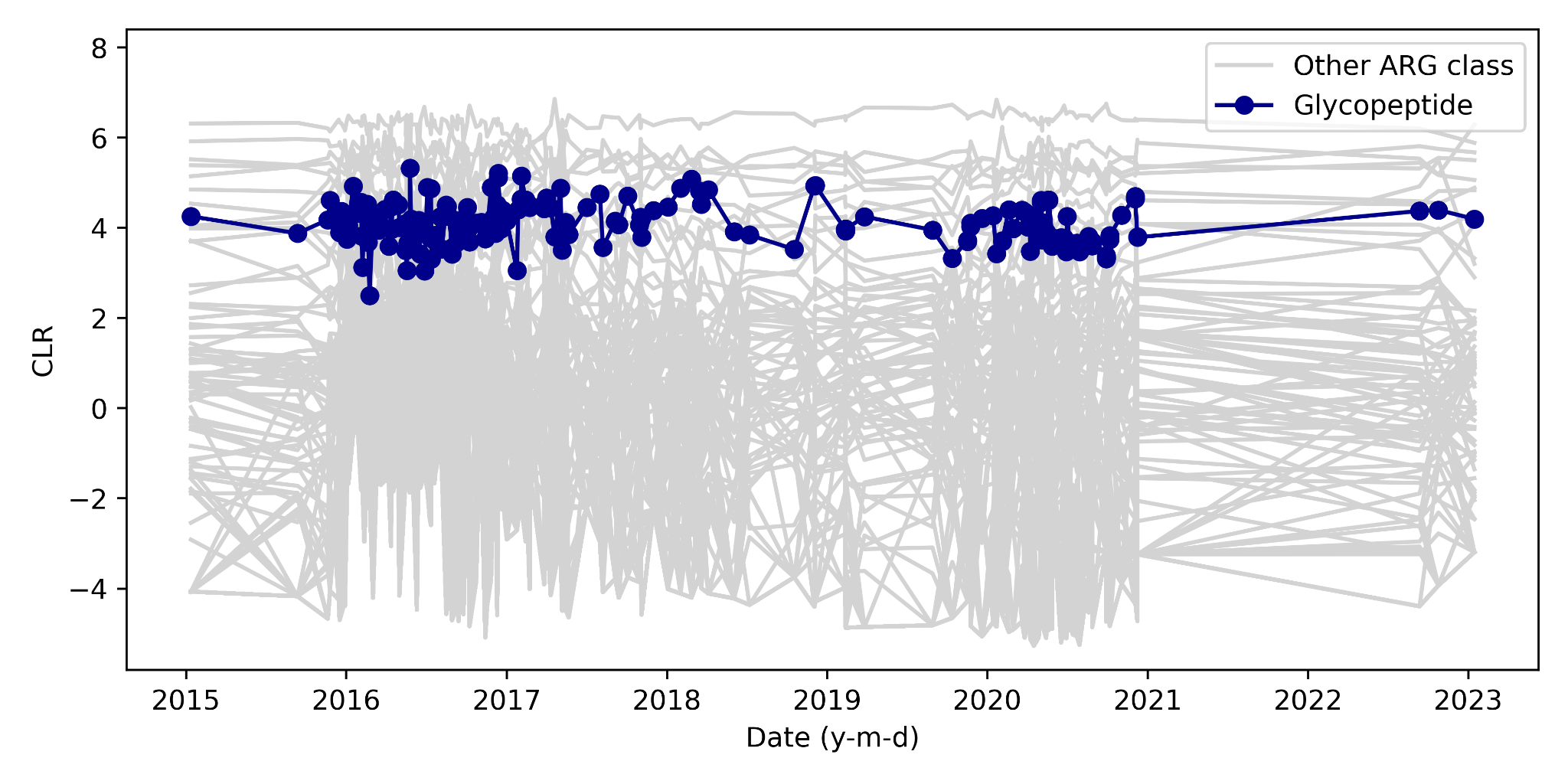


**Supplementary table 2**: Summary table of the 22 identified *vanHAX* gene clusters within the assembled metagenomic contigs. The table contains the contig sequence name (contig name), the start position of the target gene (start), the end position of the target gene (end), whether the strand is the coding (+) or the template (-) strand (strand), the length of the contig (contig length), the collection date of the sample (metadata), the observation ID (Obs ID), the variant name of the target gene (variant), the length of the flank upstream of the target gene in bp (up flank) and the length of the flank downstream of the target gene bp (down flank). The contigs used for the visualisation in Figure 2A are coloured grey.

| **Contig name** | **Start (bp)** | **End (bp)** | **Strand** | **Contig length (bp)** | **Metadata** | **Obs ID** | **Variant** | **Up flank (bp)** | **Down flank (bp)** |
| --- | --- | --- | --- | --- | --- | --- | --- | --- | --- |
| DTU_2020_1007203_1_MG_RL_CPH_Sewage_628_S35_L001_contig=k141.472694_flag=0_coverage=12.5386_length=8996 | 6289 | 8895 | - | 8996 | 2020/02/04 | DT9 | v_1 | 101 | 6289 |
| DTU_2020_1007216_1_MG_RL_CPH_Sewage_642_S10_L001_contig=k141.598620_flag=0_coverage=19.0291_length=7700 | 4993 | 7599 | - | 7700 | 2020/03/05 | DT12 | v_1 | 101 | 4993 |
| DTU_2020_1007183_2_MG_RL_CPH_Sewage_605_S5_L001_contig=k141.220326_flag=0_coverage=10.5869_length=6750 | 102 | 2708 | + | 6750 | 2019/11/24 | DT3 | v_1 | 102 | 4042 |
| DTU_2020_1007219_2_MG_RL_CPH_Sewage_646_S155_L004_contig=k141.778204_flag=0_coverage=22.0000_length=2895 | 102 | 2708 | + | 2895 | 2020/03/12 | DT15 | v_1 | 102 | 187 |
| DTU_2020_1007261_1_MG_RL_CPH_Sewage_722_S120_L003_contig=k141.59379_flag=1_coverage=5.0000_length=3494 | 580 | 3186 | - | 3494 | 2020/07/29 | DT27 | v_1 | 308 | 580 |
| DTU_2020_1007186_2_MG_RL_CPH_Sewage_608_S27_L001_contig=k141.304762_flag=1_coverage=9.1216_length=4162 | 933 | 3539 | + | 4162 | 2019/12/02 | DT4 | v_1 | 933 | 623 |
| DTU_2020_1007226_1_MG_RL_CPH_Sewage_653_S47_L001_contig=k141.2164112_flag=0_coverage=9.3948_length=4027 | 1102 | 3708 | + | 4027 | 2020/04/16 | DT18 | v_1 | 1102 | 319 |
| DTU_2020_1007206_1_MG_RL_CPH_Sewage_631_S36_L001_contig=k141.731722_flag=1_coverage=6.0000_length=5312 | 1378 | 3984 | + | 5312 | 2020/02/11 | DT10 | v_1 | 1378 | 1328 |
| DTU_2020_1007233_1_MG_RL_CPH_Sewage_671_S49_L001_contig=k141.302991_flag=1_coverage=7.0000_length=6139 | 2139 | 4745 | - | 6139 | 2020/05/14 | DT20 | v_1 | 1394 | 2139 |
| DTU_2020_1007216_2_MG_RL_CPH_Sewage_642_S101_L003_contig=k141.147418_flag=1_coverage=9.0000_length=11514 | 6057 | 8663 | + | 11514 | 2020/03/05 | DT13 | v_1 | 6057 | 2851 |
| DTU_2020_1007158_1_MG_RL_CPH_Sewage_518_S19_L001_contig=k141.1302651_flag=0_coverage=20.9198_length=11118 | 6088 | 8694 | + | 11118 | 2019/03/27 | DT1 | v_1 | 6088 | 2424 |
| DTU_2020_1007192_1_MG_RL_CPH_Sewage_617_S74_L002_contig=k141.969396_flag=0_coverage=12.0000_length=8899 | 6106 | 8712 | + | 8899 | 2020/01/07 | DT6 | v_1 | 6106 | 187 |
| DTU_2020_1007219_1_MG_RL_CPH_Sewage_646_S11_L001_contig=k141.834467_flag=0_coverage=20.1061_length=9073 | 6148 | 8754 | + | 9073 | 2020/03/12 | DT14 | v_1 | 6148 | 319 |
| DTU_2020_1007222_1_MG_RL_CPH_Sewage_649_S13_L001_contig=k141.1124866_flag=1_coverage=20.9603_length=11234 | 6152 | 8758 | + | 11234 | 2020/03/20 | DT16 | v_1 | 6152 | 2476 |
| DTU_2020_1007209_1_MG_RL_CPH_Sewage_634_S81_L002_contig=k141.97176_flag=1_coverage=20.9523_length=9793 | 1031 | 3637 | - | 9793 | 2020/02/19 | DT11 | v_1 | 6156 | 1031 |
| DTU_2020_1007200_1_MG_RL_CPH_Sewage_625_S78_L002_contig=k141.998976_flag=0_coverage=27.2334_length=11620 | 6158 | 8764 | + | 11620 | 2020/02/29 | DT8 | v_1 | 6158 | 2856 |
| DTU_2020_1007224_1_MG_RL_CPH_Sewage_651_S45_L001_contig=k141.1792110_flag=0_coverage=26.1828_length=9307 | 6158 | 8764 | + | 9307 | 2020/03/26 | DT17 | v_1 | 6158 | 543 |
| DTU_2020_1007281_1_MG_RL_CPH_Sewage_1_677_S109_L003_contig=k141.437863_flag=0_coverage=34.9180_length=9259 | 6158 | 8764 | + | 9259 | 2020/05/20 | DT28 | v_1 | 6158 | 495 |
| DTU_2020_1007195_1_MG_RL_CPH_Sewage_620_S32_L001_contig=k141.357718_flag=0_coverage=11.9463_length=9146 | 6221 | 8827 | + | 9146 | 2020/01/14 | DT7 | v_1 | 6221 | 319 |
| DTU_2020_1007189_1_MG_RL_CPH_Sewage_614_S73_L002_contig=k141.996307_flag=0_coverage=25.9388_length=9337 | 6544 | 9150 | + | 9337 | 2019/12/20 | DT5 | v_1 | 6544 | 187 |
| DTU_2020_1007227_1_MG_RL_CPH_Sewage_654_S71_L002_contig=k141.1235928_flag=0_coverage=34.0000_length=11174 | 1876 | 4482 | - | 11174 | 2020/04/27 | DT19 | v_1 | 6692 | 1876 |
| DTU_2020_1007282_1_MG_RL_CPH_Sewage_2_677_S110_L003_contig=k141.690041_flag=0_coverage=42.9164_length=16677 | 4584 | 7190 | - | 16677 | 2020/05/20 | DT29 | v_1 | 9487 | 4584 |

**Supplementary figure 2: A)** Longest contig containing *vanHAX* (9486 bp downstream flank and 4584 bp upstream flank). **B)** Longest contig containing *vanHBX* (25456 bp downstream flank and 3479 bp upstream flank).

**A**


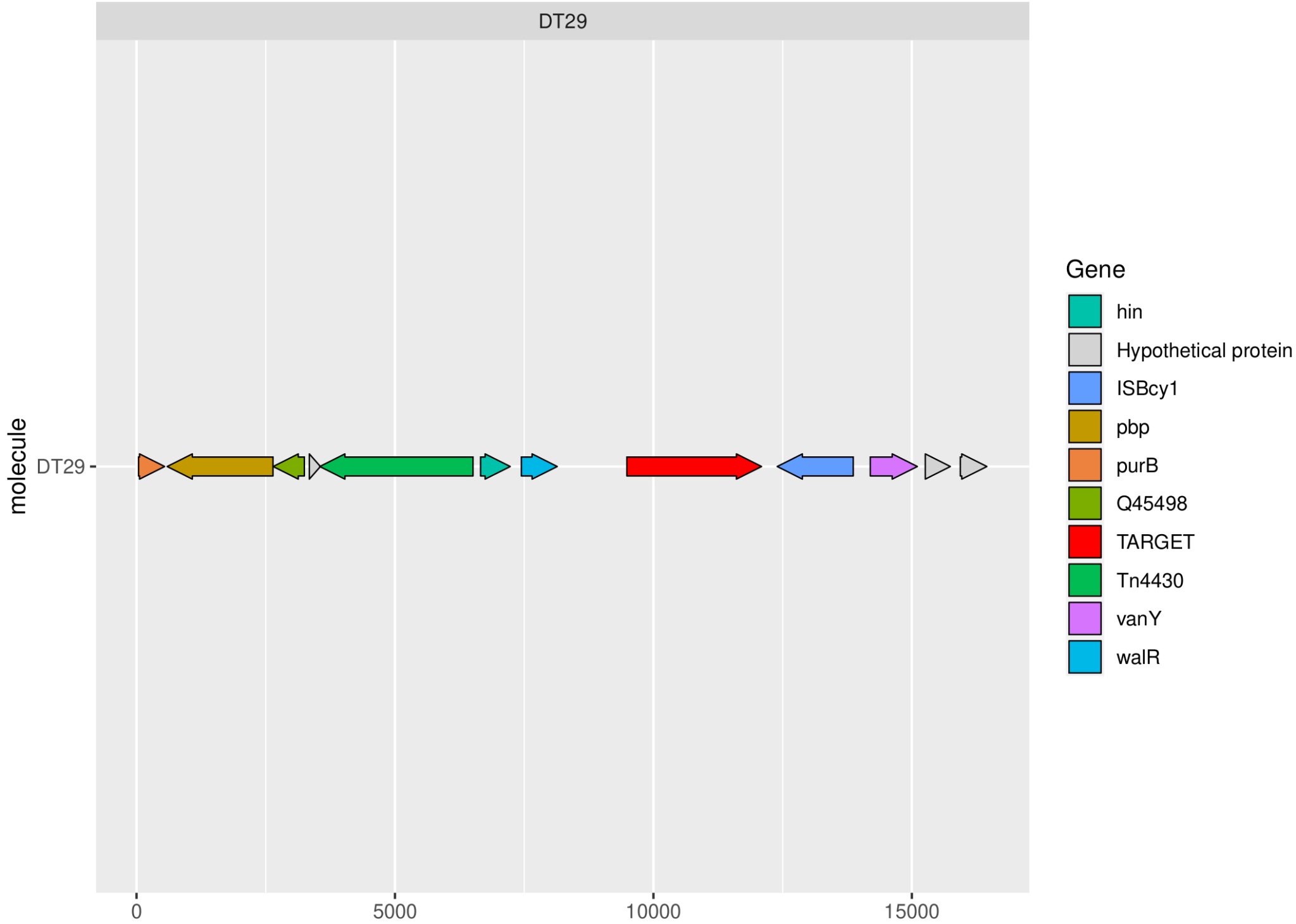


**B**


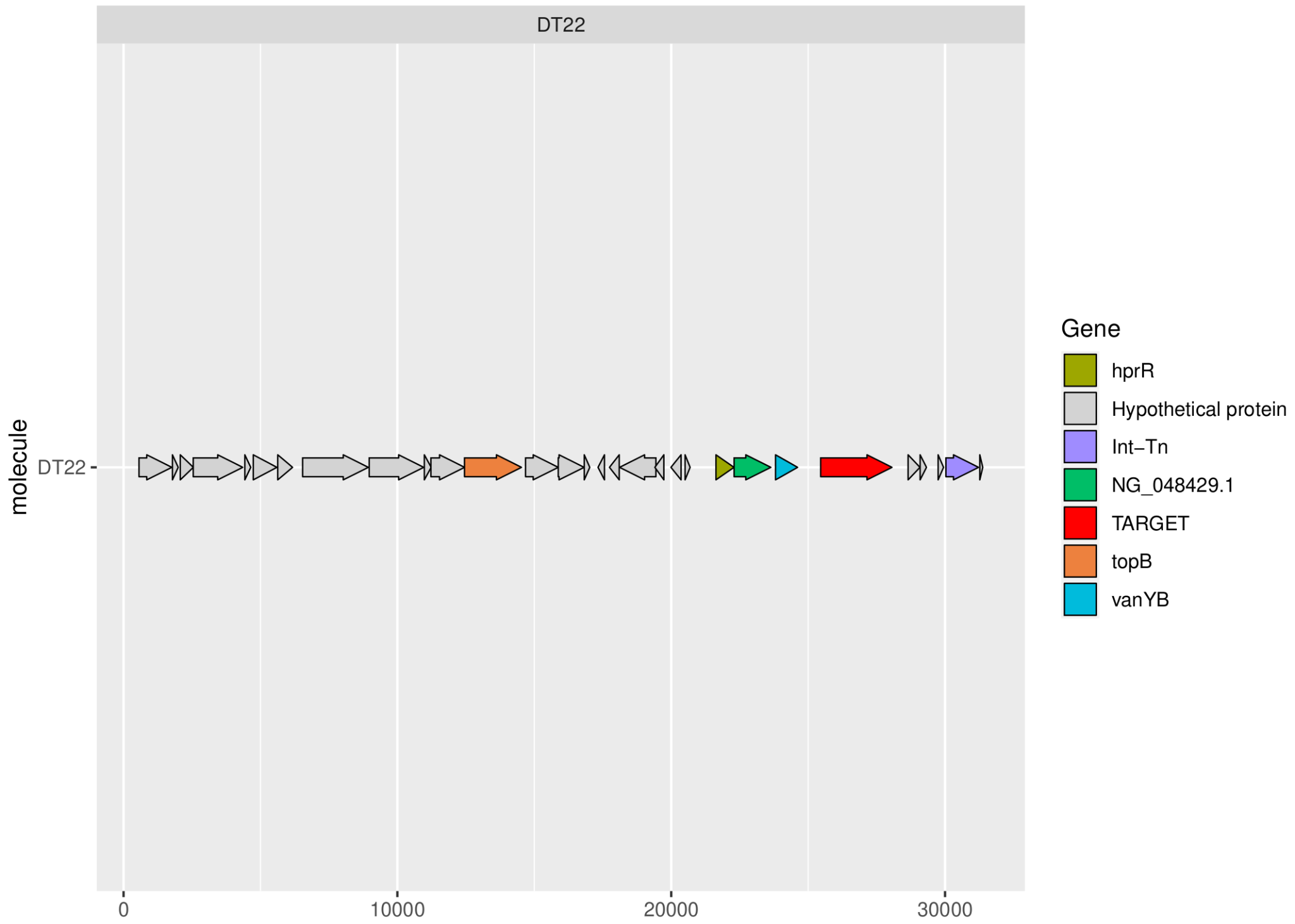


**Supplementary table 3**: Summary table of the 2 identified *vanHBX* gene clusters within the assembled metagenomic contigs. The table contains the contig sequence name (contig name), the start position of the target gene (start), the end position of the target gene (end), whether the strand is the coding (+) or the template (-) strand (strand), the length of the contig (contig length), the collection date of the sample (metadata), the observation ID (Obs ID), the variant name of the target gene (variant), the length of the flank upstream of the target gene in bp (up flank) and the length of the flank downstream of the target gene bp (down flank). The contigs used for the visualisation in Figure 2B are coloured grey.

| **Contig name** | **Start (bp)** | **End (bp)** | **Strand** | **Contig length (bp)** | **Metadata** | **Obs ID** | **Variant** | **Up flank (bp)** | **Down flank (bp)** |
| --- | --- | --- | --- | --- | --- | --- | --- | --- | --- |
| DTU_2020_1007170_1_MG_RL_CPH_Sewage_571_S21_L001_contig=k141.480401_flag=1_coverage=7.0000_length=8167 | 3197 | 5803 | + | 8167 | 2019/08/29 | DT2 | v_2 | 3197 | 2364 |
| DTU_2020_1007239_1_MG_RL_CPH_Sewage_689_S53_L001_contig=k141.153683_flag=0_coverage=11.9923_length=31543 | 25457 | 28063 | + | 31543 | 2020/06/04 | DT22 | v_2 | 25457 | 3480 |

**Supplementary table 4:** Quality and taxonomic identification of the metagenomic assembled genomes using CheckM and GTDB_Tk. High quality bins (HQ, bin>=90% complete and bin=<5% contamination) are colored green and medium quality bins (MQ, bin>=70% complete and bin=<10% contamination) are colored yellow.

| **Bin** | **CheckM taxa** | **Completeness** | **Contamiantion** | **Strain heterogeneity** | **Resistance gene** | **GTDB_Tk** |
| --- | --- | --- | --- | --- | --- | --- |
| DTU_2020_1007200_1_MG_RL_CPH_Sewage_625_S78_L002.bin.67 | o__Lactobacillales | 91.17 | 3.51 | 28.57 | *vanHAX_2_m97297* | d__Bacteria;p__Firmicutes;c__Bacilli;o__Lactobacillales;f__Streptococcaceae;g__Streptococcus;s__Streptococcus |
| DTU_2020_1007216_2_MG_RL_CPH_Sewage_642_S101_L003.bin.10 | o__Lactobacillales | 96.08 | 4.95 | 27.27 | *vanHAX_2_m97297* | d__Bacteria;p__Firmicutes;c__Bacilli;o__Lactobacillales;f__Streptococcaceae;g__Streptococcus;s__Streptococcus |
| DTU_2020_1007230_3_MG_RL_CPH_Sewage_662_S70_L002.bin.44 | k__Bacteria | 92.83 | 2.89 | 14.29 | *vanHAX_2_m97297* | d__Bacteria;p__Bacteroidota;c__Bacteroidia;o__Bacteroidales;f__Paludibacteraceae;g__UPXZ01;s__UPXZ01 |
| DTU_2020_1007216_1_MG_RL_CPH_Sewage_642_S10_L001.bin.80 | o__Lactobacillales | 88.95 | 1.84 | 50 | *vanHAX_2_m97297* | d__Bacteria;p__Firmicutes;c__Bacilli;o__Lactobacillales;f__Streptococcaceae;g__Streptococcus;s__Streptococcus |
| DTU_2020_1007281_2_MG_RL_CPH_Sewage_1_677_S147_L004.bin.3 | o__Lactobacillales | 80.88 | 3.48 | 0 | *vanHAX_2_m97297* | d__Bacteria;p__Firmicutes;c__Bacilli;o__Lactobacillales;f__Aerococcaceae;g__Trichococcus;s__Trichococcus |
| DTU_2020_1007258_1_MG_RL_CPH_Sewage_719_S118_L003.bin.90 | k__Bacteria | 55.17 | 1.72 | 0 | *vanHBX_1_af192329* | d__Bacteria;p__Bacteroidota;c__Bacteroidia;o__Bacteroidales;f__Bacteroidaceae;g__Bacteroides;s__Bacteroides |
| DTU_2020_1007183_2_MG_RL_CPH_Sewage_605_S5_L001.bin.30 | k__Archaea | 60.68 | 33.99 | 5.56 | *vanHAX_2_m97297* | d__Archaea;p__Methanobacteriota;c__Methanobacteria;o__Methanobacteriales;f__Methanobacteriaceae;g__Methanobrevibacter_A;s__ |
| DTU_2020_1007192_1_MG_RL_CPH_Sewage_617_S74_L002.bin.43 | f__Flavobacteriaceae | 93.02 | 14.82 | 14.94 | *vanHAX_2_m97297* | d__Bacteria;p__Bacteroidota;c__Bacteroidia;o__Flavobacteriales;f__Flavobacteriaceae;g__Flavobacterium;s__Flavobacterium |
| DTU_2020_1007195_1_MG_RL_CPH_Sewage_620_S32_L001.bin.19 | f__Flavobacteriaceae | 87.8 | 14.44 | 16.46 | *vanHAX_2_m97297* | d__Bacteria;p__Bacteroidota;c__Bacteroidia;o__Flavobacteriales;f__Flavobacteriaceae;g__Flavobacterium;s__Flavobacterium |
| DTU_2020_1007203_1_MG_RL_CPH_Sewage_628_S35_L001.bin.34 | k__Bacteria | 54.62 | 21.79 | 7.14 | *vanHAX_2_m97297* | d__Archaea;p__Methanobacteriota;c__Methanobacteria;o__Methanobacteriales;f__Methanobacteriaceae;g__Methanobrevibacter_A;s__ |
| DTU_2020_1007203_1_MG_RL_CPH_Sewage_628_S35_L001.bin.64 | root | 0 | 0 | 0 | *vanHAX_2_m97297* | Unclassified |
| DTU_2020_1007206_1_MG_RL_CPH_Sewage_631_S36_L001.bin.101 | k__Bacteria | 13.79 | 0 | 0 | *vanHAX_2_m97297* | d__Bacteria;p__Firmicutes_C;c__Negativicutes;o__Veillonellales;f__Dialisteraceae;g__Dialister;s__Dialister |
| DTU_2020_1007216_1_MG_RL_CPH_Sewage_642_S10_L001.bin.36 | root | 0 | 0 | 0 | *vanHAX_2_m97297* | Unclassified |
| DTU_2020_1007216_1_MG_RL_CPH_Sewage_642_S10_L001.bin.42 | k__Archaea | 70.58 | 99.55 | 1.16 | *vanHAX_2_m97297* | d__Archaea;p__Methanobacteriota;c__Methanobacteria;o__Methanobacteriales;f__Methanobacteriaceae;g__Methanobrevibacter_A;s__ |
| DTU_2020_1007233_1_MG_RL_CPH_Sewage_671_S49_L001.bin.21 | k__Bacteria | 72.57 | 10.34 | 50 | *vanHAX_2_m97297* | d__Bacteria;p__Bacteroidota;c__Bacteroidia;o__Bacteroidales;f__Tannerellaceae;g__Macellibacteroides;s__Macellibacteroides |
| DTU_2020_1007242_2_MG_RL_CPH_Sewage_695_S61_L002.bin.68 | k__Bacteria | 99.14 | 237.58 | 8.56 | *vanHAX_2_m97297* | d__Bacteria;p__Fusobacteriota;c__Fusobacteriia;o__Fusobacteriales;f__Leptotrichiaceae;g__JAGOWQ01;s__ |
| DTU_2020_1007245_1_MG_RL_CPH_Sewage_701_S84_L002.bin.5 | o__Bacteroidales | 68.93 | 13.06 | 12.73 | *vanHAX_2_m97297* | d__Bacteria;p__Bacteroidota;c__Bacteroidia;o__Bacteroidales;f__Bacteroidaceae;g__Phocaeicola;s__Phocaeicola |
| DTU_2020_1007245_1_MG_RL_CPH_Sewage_701_S84_L002.bin.44 | k__Bacteria | 100 | 364.05 | 6.76 | *vanHAX_2_m97297* | d__Archaea;p__Methanobacteriota;c__Methanobacteria;o__Methanobacteriales;f__Methanobacteriaceae;g__Methanobrevibacter_A;s__ |
| DTU_2020_1007261_1_MG_RL_CPH_Sewage_722_S120_L003.bin.93 | k__Bacteria | 70.34 | 27.59 | 47.62 | *vanHAX_2_m97297* | d__Bacteria;p__Bacteroidota;c__Bacteroidia;o__Bacteroidales;f__Bacteroidaceae;g__Phocaeicola;s__Phocaeicola |
| DTU_2020_1007252_1_MG_RL_CPH_Sewage_713_S113_L003.bin.12 | k__Bacteria | 49.79 | 0.88 | 100 | *vanHBX_1_af192329* | d__Bacteria;p__Firmicutes_A;c__Clostridia;o__Lachnospirales;f__Lachnospiraceae;g__Eisenbergiella;s__Eisenbergiella |

**Supplementary table 5**: Taxonomy assignment of the 40 *vanHAX*-containing contigs using a BLASTN lowest common tax level approach (see methods). Only hits of high confidence were considered (e-value < 1e-50). Contig k141.1302651 and k141.690041 were resolved using mmseqs2 with the LCA algorithm using the GTDB database after ambiguous classification by BLASTN.

| **Contig** | **Species** | **Genus** | **Family** | **Order** | **Class** | **Phylum** |
| --- | --- | --- | --- | --- | --- | --- |
| DTU_2020_1007206_1_MG_RL_CPH_Sewage_631_S36_L001_contig=k141.731722_flag=1_coverage=6.0000_length=5312 | Enterococcus faecium | Enterococcus | Enterococcaceae | Lactobacillales | Bacilli | Bacillota |
| DTU_2020_1007233_1_MG_RL_CPH_Sewage_671_S49_L001_contig=k141.302991_flag=1_coverage=7.0000_length=6139 | Enterococcus faecium | Enterococcus | Enterococcaceae | Lactobacillales | Bacilli | Bacillota |
| DTU_2020_1007203_1_MG_RL_CPH_Sewage_628_S35_L001_contig=k141.472694_flag=0_coverage=12.5386_length=8996 |  |  |  | Lactobacillales | Bacilli | Bacillota |
| DTU_2020_1007158_1_MG_RL_CPH_Sewage_518_S19_L001_contig=k141.1302651_flag=0_coverage=20.9198_length=11118 | Enterococcus C saigonensis | Enterococcus | Enterococcaceae | Lactobacillales | Bacilli | Bacillota |
| DTU_2020_1007183_2_MG_RL_CPH_Sewage_605_S5_L001_contig=k141.1475009_flag=0_coverage=10.9653_length=6477 |  |  |  |  | Bacilli | Bacillota |
| DTU_2020_1007209_1_MG_RL_CPH_Sewage_634_S81_L002_contig=k141.97176_flag=1_coverage=20.9523_length=9793 |  |  |  |  | Bacilli | Bacillota |
| DTU_2020_1007189_1_MG_RL_CPH_Sewage_614_S73_L002_contig=k141.996307_flag=0_coverage=25.9388_length=9337 |  |  |  |  | Bacilli | Bacillota |
| DTU_2020_1007192_1_MG_RL_CPH_Sewage_617_S74_L002_contig=k141.969396_flag=0_coverage=12.0000_length=8899 |  |  |  |  | Bacilli | Bacillota |
| DTU_2020_1007195_1_MG_RL_CPH_Sewage_620_S32_L001_contig=k141.357718_flag=0_coverage=11.9463_length=9146 |  |  |  |  | Bacilli | Bacillota |
| DTU_2020_1007200_1_MG_RL_CPH_Sewage_625_S78_L002_contig=k141.998976_flag=0_coverage=27.2334_length=11620 |  |  |  |  | Bacilli | Bacillota |
| DTU_2020_1007200_1_MG_RL_CPH_Sewage_625_S78_L002_contig=k141.1853072_flag=0_coverage=30.4378_length=1315 |  |  |  |  | Bacilli | Bacillota |
| DTU_2020_1007216_1_MG_RL_CPH_Sewage_642_S10_L001_contig=k141.551381_flag=0_coverage=5.9738_length=4343 |  |  |  |  | Bacilli | Bacillota |
| DTU_2020_1007203_1_MG_RL_CPH_Sewage_628_S35_L001_contig=k141.1545277_flag=0_coverage=3.9122_length=1644 |  |  |  |  | Bacilli | Bacillota |
| DTU_2020_1007203_1_MG_RL_CPH_Sewage_628_S35_L001_contig=k141.1054120_flag=0_coverage=10.9633_length=6136 |  |  |  |  | Bacilli | Bacillota |
| DTU_2020_1007216_1_MG_RL_CPH_Sewage_642_S10_L001_contig=k141.589603_flag=0_coverage=19.8588_length=6196 |  |  |  |  | Bacilli | Bacillota |
| DTU_2020_1007216_2_MG_RL_CPH_Sewage_642_S101_L003_contig=k141.147418_flag=1_coverage=9.0000_length=11514 |  |  |  |  | Bacilli | Bacillota |
| DTU_2020_1007219_1_MG_RL_CPH_Sewage_646_S11_L001_contig=k141.834467_flag=0_coverage=20.1061_length=9073 |  |  |  |  | Bacilli | Bacillota |
| DTU_2020_1007219_2_MG_RL_CPH_Sewage_646_S155_L004_contig=k141.893480_flag=0_coverage=18.9337_length=6114 |  |  |  |  | Bacilli | Bacillota |
| DTU_2020_1007282_1_MG_RL_CPH_Sewage_2_677_S110_L003_contig=k141.690041_flag=0_coverage=42.9164_length=16677 | Enterococcus C saigonensis | Enterococcus | Enterococcaceae | Lactobacillales | Bacilli | Bacillota |
| DTU_2020_1007222_1_MG_RL_CPH_Sewage_649_S13_L001_contig=k141.1124866_flag=1_coverage=20.9603_length=11234 |  |  |  |  | Bacilli | Bacillota |
| DTU_2020_1007281_1_MG_RL_CPH_Sewage_1_677_S109_L003_contig=k141.437863_flag=0_coverage=34.9180_length=9259 |  |  |  |  | Bacilli | Bacillota |
| DTU_2020_1007224_1_MG_RL_CPH_Sewage_651_S45_L001_contig=k141.1792110_flag=0_coverage=26.1828_length=9307 |  |  |  |  | Bacilli | Bacillota |
| DTU_2020_1007242_2_MG_RL_CPH_Sewage_695_S61_L002_contig=k141.528624_flag=1_coverage=5.0000_length=2158 |  |  |  |  | Bacilli | Bacillota |
| DTU_2020_1007245_1_MG_RL_CPH_Sewage_701_S84_L002_contig=k141.1213296_flag=1_coverage=7.0000_length=3065 |  |  |  |  | Bacilli | Bacillota |
| DTU_2020_1007227_1_MG_RL_CPH_Sewage_654_S71_L002_contig=k141.1235928_flag=0_coverage=34.0000_length=11174 |  |  |  |  | Bacilli | Bacillota |
| DTU_2020_1007230_2_MG_RL_CPH_Sewage_662_S421_L004_contig=k141.61240_flag=1_coverage=4.0000_length=1663 |  |  |  |  |  |  |
| DTU_2020_1007176_1_MG_RL_CPH_Sewage_597_S23_L001_contig=k141.88194_flag=1_coverage=4.0000_length=1270 |  |  |  |  |  |  |
| DTU_2020_1007180_2_MG_RL_CPH_Sewage_602_S60_L002_contig=k141.755715_flag=1_coverage=6.0000_length=3062 |  |  |  |  |  |  |
| DTU_2020_1007183_2_MG_RL_CPH_Sewage_605_S5_L001_contig=k141.220326_flag=0_coverage=10.5869_length=6750 |  |  |  |  |  |  |
| DTU_2020_1007186_2_MG_RL_CPH_Sewage_608_S27_L001_contig=k141.304762_flag=1_coverage=9.1216_length=4162 |  |  |  |  |  |  |
| DTU_2020_1007216_1_MG_RL_CPH_Sewage_642_S10_L001_contig=k141.598620_flag=0_coverage=19.0291_length=7700 |  |  |  |  |  |  |
| DTU_2020_1007219_2_MG_RL_CPH_Sewage_646_S155_L004_contig=k141.778204_flag=0_coverage=22.0000_length=2895 |  |  |  |  |  |  |
| DTU_2020_1007226_1_MG_RL_CPH_Sewage_653_S47_L001_contig=k141.2164112_flag=0_coverage=9.3948_length=4027 |  |  |  |  |  |  |
| DTU_2020_1007230_3_MG_RL_CPH_Sewage_662_S70_L002_contig=k141.468504_flag=1_coverage=5.0000_length=1784 |  |  |  |  |  |  |
| DTU_2020_1007230_3_MG_RL_CPH_Sewage_662_S70_L002_contig=k141.1151667_flag=1_coverage=5.0000_length=1232 |  |  |  |  |  |  |
| DTU_2020_1007236_2_MG_RL_CPH_Sewage_683_S66_L002_contig=k141.1204437_flag=1_coverage=5.0000_length=3810 |  |  |  |  |  |  |
| DTU_2020_1007242_2_MG_RL_CPH_Sewage_695_S61_L002_contig=k141.935855_flag=1_coverage=9.0000_length=3065 |  |  |  |  |  |  |
| DTU_2020_1007245_1_MG_RL_CPH_Sewage_701_S84_L002_contig=k141.1329984_flag=1_coverage=7.0000_length=4067 |  |  |  |  |  |  |
| DTU_2020_1007261_1_MG_RL_CPH_Sewage_722_S120_L003_contig=k141.59379_flag=1_coverage=5.0000_length=3494 |  |  |  |  |  |  |
| DTU_2020_1007281_2_MG_RL_CPH_Sewage_1_677_S147_L004_contig=k141.106793_flag=1_coverage=4.0000_length=2459 |  |  |  |  |  |  |

**Supplementary table 6**: Taxonomy assignment of the 38 *vanHBX*-containing contigs using a BLASTN lowest common tax level approach (see methods). Only hits of high confidence were considered (e-value < 1e-50).

| **Contig** | **Species** | **Genus** | **Family** | **Order** | **Class** | **Phylum** |
| --- | --- | --- | --- | --- | --- | --- |
| DTU_2020_1007247_1_MG_RL_CPH_Sewage_707_S99_L003_contig=k141.1226461_flag=1_coverage=3.0000_length=2304 | Enterococcus faecalis | Enterococcus | Enterococcaceae | Lactobacillales | Bacilli | Bacillota |
| DTU_2020_1007176_1_MG_RL_CPH_Sewage_597_S23_L001_contig=k141.380984_flag=1_coverage=4.0000_length=1004 | Enterococcus faecium | Enterococcus | Enterococcaceae | Lactobacillales | Bacilli | Bacillota |
| DTU_2020_1007183_2_MG_RL_CPH_Sewage_605_S5_L001_contig=k141.1471296_flag=1_coverage=3.0000_length=1302 | Enterococcus faecium | Enterococcus | Enterococcaceae | Lactobacillales | Bacilli | Bacillota |
| DTU_2020_1007255_1_MG_RL_CPH_Sewage_716_S116_L003_contig=k141.849225_flag=1_coverage=4.0000_length=3242 | Enterococcus faecium | Enterococcus | Enterococcaceae | Lactobacillales | Bacilli | Bacillota |
| DTU_2020_1007272_1_MG_RL_CPH_Sewage_740_S127_L003_contig=k141.896025_flag=1_coverage=4.0000_length=1073 | Enterococcus faecium | Enterococcus | Enterococcaceae | Lactobacillales | Bacilli | Bacillota |
| DTU_2020_1007272_1_MG_RL_CPH_Sewage_740_S127_L003_contig=k141.654893_flag=1_coverage=5.0000_length=1268 | Enterococcus faecium | Enterococcus | Enterococcaceae | Lactobacillales | Bacilli | Bacillota |
| DTU_2020_1007281_1_MG_RL_CPH_Sewage_1_677_S109_L003_contig=k141.1109679_flag=1_coverage=4.0000_length=1432 | Enterococcus faecium | Enterococcus | Enterococcaceae | Lactobacillales | Bacilli | Bacillota |
| DTU_2020_1007282_1_MG_RL_CPH_Sewage_2_677_S110_L003_contig=k141.1515673_flag=1_coverage=3.0000_length=1332 | Enterococcus faecium | Enterococcus | Enterococcaceae | Lactobacillales | Bacilli | Bacillota |
| DTU_2020_1007236_2_MG_RL_CPH_Sewage_683_S66_L002_contig=k141.1084553_flag=1_coverage=5.0000_length=1239 |  |  |  |  | Bacilli | Bacillota |
| DTU_2020_1007170_1_MG_RL_CPH_Sewage_571_S21_L001_contig=k141.480401_flag=1_coverage=7.0000_length=8167 |  |  |  |  |  | Bacillota |
| DTU_2020_1007226_1_MG_RL_CPH_Sewage_653_S47_L001_contig=k141.304491_flag=1_coverage=5.0000_length=2633 |  |  |  |  |  | Bacillota |
| DTU_2020_1007227_1_MG_RL_CPH_Sewage_654_S71_L002_contig=k141.924412_flag=1_coverage=5.0000_length=3439 |  |  |  |  |  | Bacillota |
| DTU_2020_1007227_1_MG_RL_CPH_Sewage_654_S71_L002_contig=k141.630108_flag=1_coverage=5.0000_length=3943 |  |  |  |  |  |  |
| DTU_2020_1007239_1_MG_RL_CPH_Sewage_689_S53_L001_contig=k141.153683_flag=0_coverage=11.9923_length=31543 |  |  |  |  |  |  |
| DTU_2020_1007242_2_MG_RL_CPH_Sewage_695_S61_L002_contig=k141.1155035_flag=1_coverage=6.0000_length=1607 |  |  |  |  |  |  |
| DTU_2020_1007176_1_MG_RL_CPH_Sewage_597_S23_L001_contig=k141.283653_flag=1_coverage=4.0000_length=2137 |  |  |  |  |  |  |
| DTU_2020_1007245_1_MG_RL_CPH_Sewage_701_S84_L002_contig=k141.1786949_flag=1_coverage=5.0000_length=5604 |  |  |  |  |  |  |
| DTU_2020_1007261_1_MG_RL_CPH_Sewage_722_S120_L003_contig=k141.768631_flag=1_coverage=4.0000_length=1202 |  |  |  |  |  |  |
| DTU_2020_1007276_1_MG_RL_CPH_Sewage_2_586_S106_L003_contig=k141.436631_flag=1_coverage=4.0000_length=1064 |  |  |  |  |  |  |
| DTU_2020_1007158_1_MG_RL_CPH_Sewage_518_S19_L001_contig=k141.1068717_flag=1_coverage=6.0000_length=1168 |  |  |  |  |  |  |
| DTU_2020_1007183_2_MG_RL_CPH_Sewage_605_S5_L001_contig=k141.911556_flag=1_coverage=3.0000_length=1818 |  |  |  |  |  |  |
| DTU_2020_1007189_1_MG_RL_CPH_Sewage_614_S73_L002_contig=k141.655769_flag=1_coverage=5.0000_length=1619 |  |  |  |  |  |  |
| DTU_2020_1007192_1_MG_RL_CPH_Sewage_617_S74_L002_contig=k141.1796091_flag=1_coverage=5.0000_length=1200 |  |  |  |  |  |  |
| DTU_2020_1007203_1_MG_RL_CPH_Sewage_628_S35_L001_contig=k141.654288_flag=1_coverage=5.0000_length=2340 |  |  |  |  |  |  |
| DTU_2020_1007209_1_MG_RL_CPH_Sewage_634_S81_L002_contig=k141.1734332_flag=1_coverage=2.0000_length=1999 |  |  |  |  |  |  |
| DTU_2020_1007222_1_MG_RL_CPH_Sewage_649_S13_L001_contig=k141.1070558_flag=1_coverage=3.0000_length=1121 |  |  |  |  |  |  |
| DTU_2020_1007224_1_MG_RL_CPH_Sewage_651_S45_L001_contig=k141.316039_flag=1_coverage=5.0000_length=1008 |  |  |  |  |  |  |
| DTU_2020_1007224_1_MG_RL_CPH_Sewage_651_S45_L001_contig=k141.616910_flag=1_coverage=3.0000_length=1021 |  |  |  |  |  |  |
| DTU_2020_1007233_1_MG_RL_CPH_Sewage_671_S49_L001_contig=k141.85788_flag=1_coverage=4.0000_length=1720 |  |  |  |  |  |  |
| DTU_2020_1007245_1_MG_RL_CPH_Sewage_701_S84_L002_contig=k141.429197_flag=1_coverage=5.0000_length=1496 |  |  |  |  |  |  |
| DTU_2020_1007247_1_MG_RL_CPH_Sewage_707_S99_L003_contig=k141.1425620_flag=1_coverage=3.0000_length=1337 |  |  |  |  |  |  |
| DTU_2020_1007249_1_MG_RL_CPH_Sewage_710_S135_L003_contig=k141.1369584_flag=1_coverage=5.0000_length=3796 |  |  |  |  |  |  |
| DTU_2020_1007252_1_MG_RL_CPH_Sewage_713_S113_L003_contig=k141.674571_flag=1_coverage=3.0000_length=2490 |  |  |  |  |  |  |
| DTU_2020_1007258_1_MG_RL_CPH_Sewage_719_S118_L003_contig=k141.1019652_flag=1_coverage=5.0000_length=3298 |  |  |  |  |  |  |
| DTU_2020_1007264_1_MG_RL_CPH_Sewage_725_S138_L003_contig=k141.769187_flag=1_coverage=5.0000_length=1194 |  |  |  |  |  |  |
| DTU_2020_1007264_1_MG_RL_CPH_Sewage_725_S138_L003_contig=k141.1119708_flag=1_coverage=4.0000_length=2213 |  |  |  |  |  |  |
| DTU_2020_1007267_1_MG_RL_CPH_Sewage_731_S124_L003_contig=k141.414725_flag=1_coverage=4.0000_length=1145 |  |  |  |  |  |  |
| DTU_2020_1007281_1_MG_RL_CPH_Sewage_1_677_S109_L003_contig=k141.1087927_flag=1_coverage=3.0000_length=3131 |  |  |  |  |  |  |

**Supplementary table 7***:* Taxonomic annotation comparison analysis of sequences containing the *vanHAX* resistance gene cluster (MAG vs contig based approach). Taxonomic annotation was performed on each contig using a BLASTN lowest common taxa approach (see methods). If a contig was placed in a bin, the taxonomic information obtained from CheckM was annotated as well. In addition, if the bin was of high quality (HQ, bin>=90% complete and bin=<5% contamination) or medium quality (MQ, bin>=70% complete and bin=<10% contamination), the taxonomic information from GTDB_Tk was annotated. Contig k141.1302651 and k141.690041 were resolved using mmseqs2 with the LCA algorithm using the GTDB database after ambiguous classification by BLASTN.

| **Contig** | **Contig taxa (BLASTN, 1e-50)** | **Bin** | **Bin taxa (CheckM)** | **MAG taxa (GTDB_Tk)** |
| --- | --- | --- | --- | --- |
| DTU_2020_1007206_1_MG_RL_CPH_Sewage_631_S36_L001_contig=k141.731722_flag=1_coverage=6.0000_length=5312 | p__Bacillota;c_Bacilli;o__Lactobacillales;f__Enterococcaceae;g__Enterococcus;s__Enterococcus_faecium | DTU_2020_1007206_1_MG_RL_CPH_Sewage_631_S36_L001.bin.101 | k__Bacteria |  |
| DTU_2020_1007233_1_MG_RL_CPH_Sewage_671_S49_L001_contig=k141.302991_flag=1_coverage=7.0000_length=6139 | p__Bacillota;c_Bacilli;o__Lactobacillales;f__Enterococcaceae;g__Enterococcus;s__Enterococcus_faecium | DTU_2020_1007233_1_MG_RL_CPH_Sewage_671_S49_L001.bin.21 | k__Bacteria |  |
| DTU_2020_1007203_1_MG_RL_CPH_Sewage_628_S35_L001_contig=k141.472694_flag=0_coverage=12.5386_length=8996 | p__Bacillota;c__Bacilli;o__Lactobacillales;g__Enterococcus | DTU_2020_1007203_1_MG_RL_CPH_Sewage_628_S35_L001.bin.64 | root |  |
| DTU_2020_1007158_1_MG_RL_CPH_Sewage_518_S19_L001_contig=k141.1302651_flag=0_coverage=20.9198_length=11118 | p__Bacillota;c__Bacilli;o__Lactobacillales;f__Enterococcaceae;g__Enterococcus;s__Enterococcus C saigonensis |  |  |  |
| DTU_2020_1007183_2_MG_RL_CPH_Sewage_605_S5_L001_contig=k141.1475009_flag=0_coverage=10.9653_length=6477 | p__Bacillota;c__Bacilli |  |  |  |
| DTU_2020_1007209_1_MG_RL_CPH_Sewage_634_S81_L002_contig=k141.97176_flag=1_coverage=20.9523_length=9793 | p__Bacillota;c__Bacilli |  |  |  |
| DTU_2020_1007189_1_MG_RL_CPH_Sewage_614_S73_L002_contig=k141.996307_flag=0_coverage=25.9388_length=9337 | p__Bacillota;c__Bacilli |  |  |  |
| DTU_2020_1007192_1_MG_RL_CPH_Sewage_617_S74_L002_contig=k141.969396_flag=0_coverage=12.0000_length=8899 | p__Bacillota;c__Bacilli | DTU_2020_1007192_1_MG_RL_CPH_Sewage_617_S74_L002.bin.43 | f__Flavobacteriaceae |  |
| DTU_2020_1007195_1_MG_RL_CPH_Sewage_620_S32_L001_contig=k141.357718_flag=0_coverage=11.9463_length=9146 | p__Bacillota;c__Bacilli | DTU_2020_1007195_1_MG_RL_CPH_Sewage_620_S32_L001.bin.19 | f__Flavobacteriaceae |  |
| DTU_2020_1007200_1_MG_RL_CPH_Sewage_625_S78_L002_contig=k141.998976_flag=0_coverage=27.2334_length=11620 | p__Bacillota;c__Bacilli | DTU_2020_1007200_1_MG_RL_CPH_Sewage_625_S78_L002.bin.67 | o__Lactobacillales | d__Bacteria;p__Firmicutes;c__Bacilli;o__Lactobacillales;f__Streptococcaceae;g__Streptococcus;s__Streptococcus |
| DTU_2020_1007200_1_MG_RL_CPH_Sewage_625_S78_L002_contig=k141.1853072_flag=0_coverage=30.4378_length=1315 | p__Bacillota;c__Bacilli |  |  |  |
| DTU_2020_1007216_1_MG_RL_CPH_Sewage_642_S10_L001_contig=k141.551381_flag=0_coverage=5.9738_length=4343 | p__Bacillota;c__Bacilli | DTU_2020_1007216_1_MG_RL_CPH_Sewage_642_S10_L001.bin.42 | k__Archaea |  |
| DTU_2020_1007203_1_MG_RL_CPH_Sewage_628_S35_L001_contig=k141.1545277_flag=0_coverage=3.9122_length=1644 | p__Bacillota;c__Bacilli | DTU_2020_1007203_1_MG_RL_CPH_Sewage_628_S35_L001.bin.34 | k__Bacteria |  |
| DTU_2020_1007203_1_MG_RL_CPH_Sewage_628_S35_L001_contig=k141.1054120_flag=0_coverage=10.9633_length=6136 | p__Bacillota;c__Bacilli | DTU_2020_1007203_1_MG_RL_CPH_Sewage_628_S35_L001.bin.64 | root |  |
| DTU_2020_1007216_1_MG_RL_CPH_Sewage_642_S10_L001_contig=k141.589603_flag=0_coverage=19.8588_length=6196 | p__Bacillota;c__Bacilli | DTU_2020_1007216_1_MG_RL_CPH_Sewage_642_S10_L001.bin.80 | o__Lactobacillales | d__Bacteria;p__Firmicutes;c__Bacilli;o__Lactobacillales;f__Streptococcaceae;g__Streptococcus;s__Streptococcus |
| DTU_2020_1007216_2_MG_RL_CPH_Sewage_642_S101_L003_contig=k141.147418_flag=1_coverage=9.0000_length=11514 | p__Bacillota;c__Bacilli | DTU_2020_1007216_2_MG_RL_CPH_Sewage_642_S101_L003.bin.10 | o__Lactobacillales | d__Bacteria;p__Firmicutes;c__Bacilli;o__Lactobacillales;f__Streptococcaceae;g__Streptococcus;s__Streptococcus |
| DTU_2020_1007219_1_MG_RL_CPH_Sewage_646_S11_L001_contig=k141.834467_flag=0_coverage=20.1061_length=9073 | p__Bacillota;c__Bacilli |  |  |  |
| DTU_2020_1007219_2_MG_RL_CPH_Sewage_646_S155_L004_contig=k141.893480_flag=0_coverage=18.9337_length=6114 | p__Bacillota;c__Bacilli |  |  |  |
| DTU_2020_1007282_1_MG_RL_CPH_Sewage_2_677_S110_L003_contig=k141.690041_flag=0_coverage=42.9164_length=16677 | p__Bacillota;c__Bacilli;o__Lactobacillales;f__Enterococcaceae;g__Enterococcus;s__Enterococcus C saigonensis |  |  |  |
| DTU_2020_1007222_1_MG_RL_CPH_Sewage_649_S13_L001_contig=k141.1124866_flag=1_coverage=20.9603_length=11234 | p__Bacillota;c__Bacilli |  |  |  |
| DTU_2020_1007281_1_MG_RL_CPH_Sewage_1_677_S109_L003_contig=k141.437863_flag=0_coverage=34.9180_length=9259 | p__Bacillota;c__Bacilli |  |  |  |
| DTU_2020_1007224_1_MG_RL_CPH_Sewage_651_S45_L001_contig=k141.1792110_flag=0_coverage=26.1828_length=9307 | p__Bacillota;c__Bacilli |  |  |  |
| DTU_2020_1007242_2_MG_RL_CPH_Sewage_695_S61_L002_contig=k141.528624_flag=1_coverage=5.0000_length=2158 | p__Bacillota;c__Bacilli | DTU_2020_1007242_2_MG_RL_CPH_Sewage_695_S61_L002.bin.68 | k__Bacteria |  |
| DTU_2020_1007245_1_MG_RL_CPH_Sewage_701_S84_L002_contig=k141.1213296_flag=1_coverage=7.0000_length=3065 | p__Bacillota;c__Bacilli | DTU_2020_1007245_1_MG_RL_CPH_Sewage_701_S84_L002.bin.5 | o__Bacteroidales |  |
| DTU_2020_1007227_1_MG_RL_CPH_Sewage_654_S71_L002_contig=k141.1235928_flag=0_coverage=34.0000_length=11174 | p__Bacillota;c__Bacilli |  |  |  |
| DTU_2020_1007230_2_MG_RL_CPH_Sewage_662_S421_L004_contig=k141.61240_flag=1_coverage=4.0000_length=1663 |  |  |  |  |
| DTU_2020_1007176_1_MG_RL_CPH_Sewage_597_S23_L001_contig=k141.88194_flag=1_coverage=4.0000_length=1270 |  |  |  |  |
| DTU_2020_1007180_2_MG_RL_CPH_Sewage_602_S60_L002_contig=k141.755715_flag=1_coverage=6.0000_length=3062 |  |  |  |  |
| DTU_2020_1007183_2_MG_RL_CPH_Sewage_605_S5_L001_contig=k141.220326_flag=0_coverage=10.5869_length=6750 |  | DTU_2020_1007183_2_MG_RL_CPH_Sewage_605_S5_L001.bin.30 | k__Archaea |  |
| DTU_2020_1007186_2_MG_RL_CPH_Sewage_608_S27_L001_contig=k141.304762_flag=1_coverage=9.1216_length=4162 |  |  |  |  |
| DTU_2020_1007216_1_MG_RL_CPH_Sewage_642_S10_L001_contig=k141.598620_flag=0_coverage=19.0291_length=7700 |  | DTU_2020_1007216_1_MG_RL_CPH_Sewage_642_S10_L001.bin.36 | root |  |
| DTU_2020_1007219_2_MG_RL_CPH_Sewage_646_S155_L004_contig=k141.778204_flag=0_coverage=22.0000_length=2895 |  |  |  |  |
| DTU_2020_1007226_1_MG_RL_CPH_Sewage_653_S47_L001_contig=k141.2164112_flag=0_coverage=9.3948_length=4027 |  |  |  |  |
| DTU_2020_1007230_3_MG_RL_CPH_Sewage_662_S70_L002_contig=k141.468504_flag=1_coverage=5.0000_length=1784 |  | DTU_2020_1007230_3_MG_RL_CPH_Sewage_662_S70_L002.bin.44 | k__Bacteria |  |
| DTU_2020_1007230_3_MG_RL_CPH_Sewage_662_S70_L002_contig=k141.1151667_flag=1_coverage=5.0000_length=1232 |  |  |  |  |
| DTU_2020_1007236_2_MG_RL_CPH_Sewage_683_S66_L002_contig=k141.1204437_flag=1_coverage=5.0000_length=3810 |  |  |  |  |
| DTU_2020_1007242_2_MG_RL_CPH_Sewage_695_S61_L002_contig=k141.935855_flag=1_coverage=9.0000_length=3065 |  |  |  |  |
| DTU_2020_1007245_1_MG_RL_CPH_Sewage_701_S84_L002_contig=k141.1329984_flag=1_coverage=7.0000_length=4067 |  | DTU_2020_1007245_1_MG_RL_CPH_Sewage_701_S84_L002.bin.44 | k__Bacteria |  |
| DTU_2020_1007261_1_MG_RL_CPH_Sewage_722_S120_L003_contig=k141.59379_flag=1_coverage=5.0000_length=3494 |  | DTU_2020_1007261_1_MG_RL_CPH_Sewage_722_S120_L003.bin.93 | k__Bacteria |  |
| DTU_2020_1007281_2_MG_RL_CPH_Sewage_1_677_S147_L004_contig=k141.106793_flag=1_coverage=4.0000_length=2459 |  | DTU_2020_1007281_2_MG_RL_CPH_Sewage_1_677_S147_L004.bin.3 | o__Lactobacillales | d__Bacteria;p__Firmicutes;c__Bacilli;o__Lactobacillales;f__Aerococcaceae;g__Trichococcus;s__Trichococcus |

**Supplementary table 8***:* Taxonomic annotation comparison analysis of sequences containing the *vanHBX* resistance gene cluster (MAG vs contig based approach). Taxonomic annotation was performed on each contig using a BLASTN lowest common taxa approach (see methods). If a contig was placed in a bin, the taxonomic information obtained from CheckM was annotated as well. In addition, if the bin was of high quality (HQ, bin>=90% complete and bin=<5% contamination) or medium quality (MQ, bin>=70% complete and bin=<10% contamination), the taxonomic information from GTDB_Tk was annotated.

| **Contig** | **Contig taxa (BLASTN, 1e-50)** | **Bin** | **Bin taxa (CheckM)** | **MAG taxa (GTDB_Tk)** |
| --- | --- | --- | --- | --- |
| DTU_2020_1007247_1_MG_RL_CPH_Sewage_707_S99_L003_contig=k141.1226461_flag=1_coverage=3.0000_length=2304 | p__Bacillota;c__Bacilli;o__Lactobacillales;f__Enterococcaceae;g__Enterococcus;s__Enterococcus faecalis |  |  |  |
| DTU_2020_1007176_1_MG_RL_CPH_Sewage_597_S23_L001_contig=k141.380984_flag=1_coverage=4.0000_length=1004 | p__Bacillota;c__Bacilli;o__Lactobacillales;f__Enterococcaceae;g__Enterococcus;s__Enterococcus_faecium |  |  |  |
| DTU_2020_1007183_2_MG_RL_CPH_Sewage_605_S5_L001_contig=k141.1471296_flag=1_coverage=3.0000_length=1302 | p__Bacillota;c__Bacilli;o__Lactobacillales;f__Enterococcaceae;g__Enterococcus;s__Enterococcus_faecium |  |  |  |
| DTU_2020_1007255_1_MG_RL_CPH_Sewage_716_S116_L003_contig=k141.849225_flag=1_coverage=4.0000_length=3242 | p__Bacillota;c__Bacilli;o__Lactobacillales;f__Enterococcaceae;g__Enterococcus;s__Enterococcus_faecium |  |  |  |
| DTU_2020_1007272_1_MG_RL_CPH_Sewage_740_S127_L003_contig=k141.896025_flag=1_coverage=4.0000_length=1073 | p__Bacillota;c__Bacilli;o__Lactobacillales;f__Enterococcaceae;g__Enterococcus;s__Enterococcus_faecium |  |  |  |
| DTU_2020_1007272_1_MG_RL_CPH_Sewage_740_S127_L003_contig=k141.654893_flag=1_coverage=5.0000_length=1268 | p__Bacillota;c__Bacilli;o__Lactobacillales;f__Enterococcaceae;g__Enterococcus;s__Enterococcus_faecium |  |  |  |
| DTU_2020_1007281_1_MG_RL_CPH_Sewage_1_677_S109_L003_contig=k141.1109679_flag=1_coverage=4.0000_length=1432 | p__Bacillota;c__Bacilli;o__Lactobacillales;f__Enterococcaceae;g__Enterococcus;s__Enterococcus_faecium |  |  |  |
| DTU_2020_1007282_1_MG_RL_CPH_Sewage_2_677_S110_L003_contig=k141.1515673_flag=1_coverage=3.0000_length=1332 | p__Bacillota;c__Bacilli;o__Lactobacillales;f__Enterococcaceae;g__Enterococcus;s__Enterococcus_faecium |  |  |  |
| DTU_2020_1007236_2_MG_RL_CPH_Sewage_683_S66_L002_contig=k141.1084553_flag=1_coverage=5.0000_length=1239 | p__Bacillota;c__Bacilli |  |  |  |
| DTU_2020_1007170_1_MG_RL_CPH_Sewage_571_S21_L001_contig=k141.480401_flag=1_coverage=7.0000_length=8167 | p__Bacillota |  |  |  |
| DTU_2020_1007226_1_MG_RL_CPH_Sewage_653_S47_L001_contig=k141.304491_flag=1_coverage=5.0000_length=2633 | p__Bacillota |  |  |  |
| DTU_2020_1007227_1_MG_RL_CPH_Sewage_654_S71_L002_contig=k141.924412_flag=1_coverage=5.0000_length=3439 | p__Bacillota |  |  |  |
| DTU_2020_1007227_1_MG_RL_CPH_Sewage_654_S71_L002_contig=k141.630108_flag=1_coverage=5.0000_length=3943 |  |  |  |  |
| DTU_2020_1007239_1_MG_RL_CPH_Sewage_689_S53_L001_contig=k141.153683_flag=0_coverage=11.9923_length=31543 |  |  |  |  |
| DTU_2020_1007242_2_MG_RL_CPH_Sewage_695_S61_L002_contig=k141.1155035_flag=1_coverage=6.0000_length=1607 |  |  |  |  |
| DTU_2020_1007176_1_MG_RL_CPH_Sewage_597_S23_L001_contig=k141.283653_flag=1_coverage=4.0000_length=2137 |  |  |  |  |
| DTU_2020_1007245_1_MG_RL_CPH_Sewage_701_S84_L002_contig=k141.1786949_flag=1_coverage=5.0000_length=5604 |  |  |  |  |
| DTU_2020_1007261_1_MG_RL_CPH_Sewage_722_S120_L003_contig=k141.768631_flag=1_coverage=4.0000_length=1202 |  |  |  |  |
| DTU_2020_1007276_1_MG_RL_CPH_Sewage_2_586_S106_L003_contig=k141.436631_flag=1_coverage=4.0000_length=1064 |  |  |  |  |
| DTU_2020_1007158_1_MG_RL_CPH_Sewage_518_S19_L001_contig=k141.1068717_flag=1_coverage=6.0000_length=1168 |  |  |  |  |
| DTU_2020_1007183_2_MG_RL_CPH_Sewage_605_S5_L001_contig=k141.911556_flag=1_coverage=3.0000_length=1818 |  |  |  |  |
| DTU_2020_1007189_1_MG_RL_CPH_Sewage_614_S73_L002_contig=k141.655769_flag=1_coverage=5.0000_length=1619 |  |  |  |  |
| DTU_2020_1007192_1_MG_RL_CPH_Sewage_617_S74_L002_contig=k141.1796091_flag=1_coverage=5.0000_length=1200 |  |  |  |  |
| DTU_2020_1007203_1_MG_RL_CPH_Sewage_628_S35_L001_contig=k141.654288_flag=1_coverage=5.0000_length=2340 |  |  |  |  |
| DTU_2020_1007209_1_MG_RL_CPH_Sewage_634_S81_L002_contig=k141.1734332_flag=1_coverage=2.0000_length=1999 |  |  |  |  |
| DTU_2020_1007222_1_MG_RL_CPH_Sewage_649_S13_L001_contig=k141.1070558_flag=1_coverage=3.0000_length=1121 |  |  |  |  |
| DTU_2020_1007224_1_MG_RL_CPH_Sewage_651_S45_L001_contig=k141.316039_flag=1_coverage=5.0000_length=1008 |  |  |  |  |
| DTU_2020_1007224_1_MG_RL_CPH_Sewage_651_S45_L001_contig=k141.616910_flag=1_coverage=3.0000_length=1021 |  |  |  |  |
| DTU_2020_1007233_1_MG_RL_CPH_Sewage_671_S49_L001_contig=k141.85788_flag=1_coverage=4.0000_length=1720 |  |  |  |  |
| DTU_2020_1007245_1_MG_RL_CPH_Sewage_701_S84_L002_contig=k141.429197_flag=1_coverage=5.0000_length=1496 |  |  |  |  |
| DTU_2020_1007247_1_MG_RL_CPH_Sewage_707_S99_L003_contig=k141.1425620_flag=1_coverage=3.0000_length=1337 |  |  |  |  |
| DTU_2020_1007249_1_MG_RL_CPH_Sewage_710_S135_L003_contig=k141.1369584_flag=1_coverage=5.0000_length=3796 |  |  |  |  |
| DTU_2020_1007252_1_MG_RL_CPH_Sewage_713_S113_L003_contig=k141.674571_flag=1_coverage=3.0000_length=2490 |  | DTU_2020_1007252_1_MG_RL_CPH_Sewage_713_S113_L003.bin.12 | k__Bacteria |  |
| DTU_2020_1007258_1_MG_RL_CPH_Sewage_719_S118_L003_contig=k141.1019652_flag=1_coverage=5.0000_length=3298 |  | DTU_2020_1007258_1_MG_RL_CPH_Sewage_719_S118_L003.bin.90 | k__Bacteria | d__Bacteria;p__Bacteroidota;c__Bacteroidia;o__Bacteroidales;f__Bacteroidaceae;g__Bacteroides;s__Bacteroides |
| DTU_2020_1007264_1_MG_RL_CPH_Sewage_725_S138_L003_contig=k141.769187_flag=1_coverage=5.0000_length=1194 |  |  |  |  |
| DTU_2020_1007264_1_MG_RL_CPH_Sewage_725_S138_L003_contig=k141.1119708_flag=1_coverage=4.0000_length=2213 |  |  |  |  |
| DTU_2020_1007267_1_MG_RL_CPH_Sewage_731_S124_L003_contig=k141.414725_flag=1_coverage=4.0000_length=1145 |  |  |  |  |
| DTU_2020_1007281_1_MG_RL_CPH_Sewage_1_677_S109_L003_contig=k141.1087927_flag=1_coverage=3.0000_length=3131 |  |  |  |  |

**Supplementary table 9:** Summary table of the 12 Hi-C sequenced metagenomic sewage samples. The data is based on the raw Hi-C reads. The table contains the fragments per sample (Hi-C fragCount), the number of fragments aligned per sample to one of the 989 PanRes glycopeptide reference genes from either ResFinder or ResfinderFG (Hi-C Glyco fragCountAln), the percentage of aligned fragments out of the total number of fragment counts for that given sample (Hi-C Glyco percAln (%)) as well as the number of aligned fragments to the entire PanRes database (Hi-C fragCountAln) and the percentage of aligned fragments out of the total fragmentCount for that sample (Hi-C percAln %).

| **MG sample name** | **Hi-C sample name** | **Hi-C fragCount** | **Hi-C glyco fragCountAln** | **Hi-C glyco percAln (%)** | **Hi-C fragCountAln** | **Hi-C percAln (%)** | **Date of isolation** |
| --- | --- | --- | --- | --- | --- | --- | --- |
| DTU_2020_1007236_1_MG_RL_CPH_Sewage_683 | cph-683 | 3014436 | 176 | 6e-05 | 3343 | 0.00111 | 2020-05-28 |
| DTU_2020_1007200_1_MG_RL_CPH_Sewage_625 | cph-625 | 4831803 | 314 | 6e-05 | 7370 | 0.00153 | 2020-02-29 |
| DTU_2020_1007230_2_MG_RL_CPH_Sewage_662 | cph-662 | 4526983 | 170 | 4e-05 | 3917 | 0.00087 | 2020-05-05 |
| DTU_2020_1007206_1_MG_RL_CPH_Sewage_631 | cph-631 | 3020949 | 145 | 5e-05 | 1235 | 0.00041 | 2020-02-11 |
| DTU_2020_1007198_1_MG_RL_CPH_Sewage_623 | cph-623 | 3497202 | 125 | 4e-05 | 2685 | 0.00077 | 2020-01-22 |
| DTU_2020_1007227_1_MG_RL_CPH_Sewage_654 | cph-654 | 3196105 | 152 | 5e-05 | 3003 | 0.00094 | 2020-04-27 |
| DTU_2020_1007249_1_MG_RL_CPH_Sewage_710 | cph-710 | 3735852 | 74 | 2e-05 | 4433 | 0.00119 | 2020-06-29 |
| DTU_2020_1007222_1_MG_RL_CPH_Sewage_649 | cph-649 | 8342130 | 492 | 6e-05 | 5342 | 0.00064 | 2020-03-20 |
| DTU_2020_1007215_2_MG_RL_CPH_Sewage_640 | cph-640 | 3098526 | 153 | 5e-05 | 2465 | 0.0008 | 2020-04-02 |
| DTU_2020_1007239_1_MG_RL_CPH_Sewage_689 | cph-689 | 3680703 | 196 | 5e-05 | 2392 | 0.00065 | 2020-06-04 |
| DTU_2020_1007192_1_MG_RL_CPH_Sewage_617 | cph-617 | 3685263 | 255 | 7e-05 | 3170 | 0.00086 | 2020-01-07 |
| DTU_2020_1007216_2_MG_RL_CPH_Sewage_642 | cph-642 | 4178006 | 312 | 7e-05 | 3111 | 0.00074 | 2020-03-05 |

**Supplementary table 10**: Vancomycin resistance genes within the Hi-C metagenomic data. If a contig contained either the *vanHAX_2_m97297* or *vanHBX_1_af192329* gene cluster, it is listed here. If the contig belonged to one of the clusters i.e. Hi-C bins, it is listed here as well.

| **Gene** | **Sample** | **Contig** | **Hi-C cluster** |
| --- | --- | --- | --- |
| vanHBX_1_af192330 | cph-617 | k141_2199614 |  |
| vanHBX_1_af192330 | cph-625 | k141_624811 |  |
| vanHBX_1_af192330 | cph-625 | k141_169250 |  |
| vanHBX_1_af192330 | cph-625 | k141_121600 |  |
| vanHBX_1_af192330 | cph-625 | k141_57541 |  |
| vanHBX_1_af192330 | cph-640 | k141_2592145 |  |
| vanHBX_1_af192330 | cph-649 | k141_343717 |  |
| vanHBX_1_af192330 | cph-649 | k141_1043235 |  |
| vanHBX_1_af192330 | cph-649 | k141_2595780 |  |
| vanHBX_1_af192330 | cph-662 | k141_3597014 |  |
| vanHBX_1_af192330 | cph-689 | k141_10858702 |  |
| vanHBX_1_af192330 | cph-623 | k141_277946 |  |
| vanHBX_1_af192330 | cph-631 | k141_692737 |  |
| vanHBX_1_af192330 | cph-631 | k141_929121 |  |
| vanHBX_1_af192330 | cph-642 | k141_3116245 |  |
| vanHBX_1_af192330 | cph-642 | k141_483720 |  |
| vanHBX_1_af192330 | cph-642 | k141_1871856 |  |
| vanHBX_1_af192330 | cph-642 | k141_1447104 |  |
| vanHBX_1_af192330 | cph-654 | k141_410380 |  |
| vanHBX_1_af192330 | cph-654 | k141_610568 |  |
| vanHBX_1_af192330 | cph-683 | k141_3106163 |  |
| vanHBX_1_af192330 | cph-710 | k141_54845 |  |
| vanHAX_2_m97316 | cph-617 | k141_2346236 |  |
| vanHAX_2_m97316 | cph-625 | k141_2339302 |  |
| vanHAX_2_m97316 | cph-625 | k141_2317423 |  |
| vanHAX_2_m97316 | cph-625 | k141_2183710 |  |
| vanHAX_2_m97316 | cph-640 | k141_1870923 |  |
| vanHAX_2_m97316 | cph-649 | k141_1914074 | cph-649_bin_2 |
| vanHAX_2_m97316 | cph-662 | k141_4125729 |  |
| vanHAX_2_m97316 | cph-689 | k141_11877556 |  |
| vanHAX_2_m97316 | cph-689 | k141_6986355 |  |
| vanHAX_2_m97316 | cph-623 | k141_2113570 | cph-623_bin_2 |
| vanHAX_2_m97316 | cph-631 | k141_504333 |  |
| vanHAX_2_m97316 | cph-642 | k141_1571987 |  |
| vanHAX_2_m97316 | cph-642 | k141_1111964 | cph-642_bin_6 |
| vanHAX_2_m97316 | cph-642 | k141_625701 | cph-642_bin_6 |
| vanHAX_2_m97316 | cph-654 | k141_194960 |  |
| vanHAX_2_m97316 | cph-654 | k141_1755641 |  |
| vanHAX_2_m97316 | cph-654 | k141_2038509 |  |
| vanHAX_2_m97316 | cph-683 | k141_2262417 |  |
| vanHAX_2_m97316 | cph-683 | k141_1466881 | cph-683_bin_1 |
| vanHAX_2_m97316 | cph-710 | k141_589123 |  |

**Supplementary table 11**: Quality assessment of the *vanHAX_2_m97297* containing Hi-C clusters using CheckM. **A)** Quality of the clusters before decontamination. **B)** Quality of the clusters after decontamination.

**A**

|  | No. contigs before decontamination | Completeness before decontamination | Contamination before decontamination |
| --- | --- | --- | --- |
| cph-623_bin_2 | 1797 | 98.63% | 84.73% |
| cph-642_bin_6 | 480 | 91.28% | 37.48% |
| cph-649_bin_2 | 1089 | 96.72% | 49.70% |
| cph-683_bin_1 | 1498 | 27.59% | 6.9% |

**B**

|  | No. contigs after decontamination | Completeness after decontamination | Contamination after decontamination |
| --- | --- | --- | --- |
| cph-623_bin_2 | 1414 | 98.63% | 42.75% |
| cph-642_bin_6 | 416 | 89.45% | 25.20% |
| cph-649_bin_2 | 902 | 96.72% | 27.60% |
| cph-683_bin_1 | 1498 | 27.59% | 6.9% |

**Supplementary table 12***:* Linking Hi-C reads containing *vanHAX* or vanHBX to metagenomic assembled genomes (MAGs). Taxonomic annotation of these MAGs was performed to link the genes to a bacterial taxa.

| **Hi-C read** | **Bin** | **Completeness** | **Contamination** | **Strain heterogeneity** | **GTDB_TK classification** |
| --- | --- | --- | --- | --- | --- |
| NGSNJ-086:927:GW2302222891st:3:2676:28085:32283_1:N:0:CTCTATCG+TCAGGCTT_pan_6936_cph-625 | DTU_2020_1007200_1_MG_RL_CPH_Sewage_625_S78_L002.bin.67 | 91.17 | 3.51 | 28.57 | d__Bacteria;p__Firmicutes;c__Bacilli;o__Lactobacillales;f__Streptococcaceae;g__Streptococcus;s__Streptococcus parasuis |
| NGSNJ-086:927:GW2302222891st:3:2672:24306:1564_1:N:0:CTCTATCG+TCAGGCTT_pan_6936_cph-625 | DTU_2020_1007200_1_MG_RL_CPH_Sewage_625_S78_L002.bin.67 | 91.17 | 3.51 | 28.57 | d__Bacteria;p__Firmicutes;c__Bacilli;o__Lactobacillales;f__Streptococcaceae;g__Streptococcus;s__Streptococcus parasuis |
| NGSNJ-086:927:GW2302222891st:3:2667:18032:13275_1:N:0:CTCTATCG+TCAGGCTT_pan_6936_cph-625 | DTU_2020_1007200_1_MG_RL_CPH_Sewage_625_S78_L002.bin.67 | 91.17 | 3.51 | 28.57 | d__Bacteria;p__Firmicutes;c__Bacilli;o__Lactobacillales;f__Streptococcaceae;g__Streptococcus;s__Streptococcus parasuis |
| NGSNJ-086:927:GW2302222891st:3:2638:25400:22654_2:N:0:CTCTATCG+TCAGGCTT_pan_6936_cph-625 | DTU_2020_1007200_1_MG_RL_CPH_Sewage_625_S78_L002.bin.67 | 91.17 | 3.51 | 28.57 | d__Bacteria;p__Firmicutes;c__Bacilli;o__Lactobacillales;f__Streptococcaceae;g__Streptococcus;s__Streptococcus parasuis |
| NGSNJ-086:927:GW2302222891st:3:2570:24569:32581_1:N:0:CTCTATCG+TCAGGCTT_pan_6936_cph-625 | DTU_2020_1007200_1_MG_RL_CPH_Sewage_625_S78_L002.bin.67 | 91.17 | 3.51 | 28.57 | d__Bacteria;p__Firmicutes;c__Bacilli;o__Lactobacillales;f__Streptococcaceae;g__Streptococcus;s__Streptococcus parasuis |
| NGSNJ-086:927:GW2302222891st:3:2564:26612:29575_1:N:0:CTCTATCG+TCAGGCTT_pan_6936_cph-625 | DTU_2020_1007200_1_MG_RL_CPH_Sewage_625_S78_L002.bin.67 | 91.17 | 3.51 | 28.57 | d__Bacteria;p__Firmicutes;c__Bacilli;o__Lactobacillales;f__Streptococcaceae;g__Streptococcus;s__Streptococcus parasuis |
| NGSNJ-086:927:GW2302222891st:3:2557:26259:5290_2:N:0:ATCTATCG+TCAGGCTT_pan_6936_cph-625 | DTU_2020_1007200_1_MG_RL_CPH_Sewage_625_S78_L002.bin.67 | 91.17 | 3.51 | 28.57 | d__Bacteria;p__Firmicutes;c__Bacilli;o__Lactobacillales;f__Streptococcaceae;g__Streptococcus;s__Streptococcus parasuis |
| NGSNJ-086:927:GW2302222891st:3:2546:25762:33082_1:N:0:CTCTATCG+TCAGGCTT_pan_6936_cph-625 | DTU_2020_1007200_1_MG_RL_CPH_Sewage_625_S78_L002.bin.67 | 91.17 | 3.51 | 28.57 | d__Bacteria;p__Firmicutes;c__Bacilli;o__Lactobacillales;f__Streptococcaceae;g__Streptococcus;s__Streptococcus parasuis |
| NGSNJ-086:927:GW2302222891st:3:2518:12283:5635_2:N:0:CTCTATCA+TCAGGCTT_pan_6936_cph-625 | DTU_2020_1007200_1_MG_RL_CPH_Sewage_625_S78_L002.bin.67 | 91.17 | 3.51 | 28.57 | d__Bacteria;p__Firmicutes;c__Bacilli;o__Lactobacillales;f__Streptococcaceae;g__Streptococcus;s__Streptococcus parasuis |
| NGSNJ-086:927:GW2302222891st:3:2441:28465:34006_1:N:0:CTCTATCG+TCAGGCTT_pan_6936_cph-625 | DTU_2020_1007200_1_MG_RL_CPH_Sewage_625_S78_L002.bin.67 | 91.17 | 3.51 | 28.57 | d__Bacteria;p__Firmicutes;c__Bacilli;o__Lactobacillales;f__Streptococcaceae;g__Streptococcus;s__Streptococcus parasuis |
| NGSNJ-086:927:GW2302222891st:3:2425:11279:27070_2:N:0:CTCTATCG+TCAGGCTT_pan_6936_cph-625 | DTU_2020_1007200_1_MG_RL_CPH_Sewage_625_S78_L002.bin.67 | 91.17 | 3.51 | 28.57 | d__Bacteria;p__Firmicutes;c__Bacilli;o__Lactobacillales;f__Streptococcaceae;g__Streptococcus;s__Streptococcus parasuis |
| NGSNJ-086:927:GW2302222891st:3:2416:9905:20275_2:N:0:CTCTATAG+TCAGGCTT_pan_6936_cph-625 | DTU_2020_1007200_1_MG_RL_CPH_Sewage_625_S78_L002.bin.67 | 91.17 | 3.51 | 28.57 | d__Bacteria;p__Firmicutes;c__Bacilli;o__Lactobacillales;f__Streptococcaceae;g__Streptococcus;s__Streptococcus parasuis |
| NGSNJ-086:927:GW2302222891st:3:2409:3866:6089_1:N:0:CTCTATCG+TCAGGCTT_pan_6936_cph-625 | DTU_2020_1007200_1_MG_RL_CPH_Sewage_625_S78_L002.bin.67 | 91.17 | 3.51 | 28.57 | d__Bacteria;p__Firmicutes;c__Bacilli;o__Lactobacillales;f__Streptococcaceae;g__Streptococcus;s__Streptococcus parasuis |
| NGSNJ-086:927:GW2302222891st:3:2373:14262:18521_1:N:0:CTCTATCG+TCAGGCTT_pan_6936_cph-625 | DTU_2020_1007200_1_MG_RL_CPH_Sewage_625_S78_L002.bin.67 | 91.17 | 3.51 | 28.57 | d__Bacteria;p__Firmicutes;c__Bacilli;o__Lactobacillales;f__Streptococcaceae;g__Streptococcus;s__Streptococcus parasuis |
| NGSNJ-086:927:GW2302222891st:3:2360:23113:35289_1:N:0:CTCTATCG+TCAGGCTT_pan_6936_cph-625 | DTU_2020_1007200_1_MG_RL_CPH_Sewage_625_S78_L002.bin.67 | 91.17 | 3.51 | 28.57 | d__Bacteria;p__Firmicutes;c__Bacilli;o__Lactobacillales;f__Streptococcaceae;g__Streptococcus;s__Streptococcus parasuis |
| NGSNJ-086:927:GW2302222891st:3:2330:1371:17237_1:N:0:CTCTATCG+TCAGCCTT_pan_6936_cph-625 | DTU_2020_1007200_1_MG_RL_CPH_Sewage_625_S78_L002.bin.67 | 91.17 | 3.51 | 28.57 | d__Bacteria;p__Firmicutes;c__Bacilli;o__Lactobacillales;f__Streptococcaceae;g__Streptococcus;s__Streptococcus parasuis |
| NGSNJ-086:927:GW2302222891st:3:2325:18258:27445_1:N:0:CTCTATCG+TCAGGCTT_pan_6936_cph-625 | DTU_2020_1007200_1_MG_RL_CPH_Sewage_625_S78_L002.bin.67 | 91.17 | 3.51 | 28.57 | d__Bacteria;p__Firmicutes;c__Bacilli;o__Lactobacillales;f__Streptococcaceae;g__Streptococcus;s__Streptococcus parasuis |
| NGSNJ-086:927:GW2302222891st:3:2325:16975:15076_1:N:0:CTCTATCG+TCAGGCTT_pan_6936_cph-625 | DTU_2020_1007200_1_MG_RL_CPH_Sewage_625_S78_L002.bin.67 | 91.17 | 3.51 | 28.57 | d__Bacteria;p__Firmicutes;c__Bacilli;o__Lactobacillales;f__Streptococcaceae;g__Streptococcus;s__Streptococcus parasuis |
| NGSNJ-086:927:GW2302222891st:3:2311:30120:2394_1:N:0:CTCTATCG+TCAGGCTT_pan_6936_cph-625 | DTU_2020_1007200_1_MG_RL_CPH_Sewage_625_S78_L002.bin.67 | 91.17 | 3.51 | 28.57 | d__Bacteria;p__Firmicutes;c__Bacilli;o__Lactobacillales;f__Streptococcaceae;g__Streptococcus;s__Streptococcus parasuis |
| NGSNJ-086:927:GW2302222891st:3:2301:27407:31360_2:N:0:CTCTATCG+TCAGGCTT_pan_6936_cph-625 | DTU_2020_1007200_1_MG_RL_CPH_Sewage_625_S78_L002.bin.67 | 91.17 | 3.51 | 28.57 | d__Bacteria;p__Firmicutes;c__Bacilli;o__Lactobacillales;f__Streptococcaceae;g__Streptococcus;s__Streptococcus parasuis |
| NGSNJ-086:927:GW2302222891st:3:2301:21034:21292_1:N:0:CTCTATCG+TCAGGCTT_pan_6936_cph-625 | DTU_2020_1007200_1_MG_RL_CPH_Sewage_625_S78_L002.bin.67 | 91.17 | 3.51 | 28.57 | d__Bacteria;p__Firmicutes;c__Bacilli;o__Lactobacillales;f__Streptococcaceae;g__Streptococcus;s__Streptococcus parasuis |
| NGSNJ-086:927:GW2302222891st:3:2271:31177:25614_2:N:0:CTCTATCG+TCAGGCTT_pan_6936_cph-625 | DTU_2020_1007200_1_MG_RL_CPH_Sewage_625_S78_L002.bin.67 | 91.17 | 3.51 | 28.57 | d__Bacteria;p__Firmicutes;c__Bacilli;o__Lactobacillales;f__Streptococcaceae;g__Streptococcus;s__Streptococcus parasuis |
| NGSNJ-086:927:GW2302222891st:3:2267:5873:7968_1:N:0:CTCTATCG+TCAGGCTT_pan_6936_cph-625 | DTU_2020_1007200_1_MG_RL_CPH_Sewage_625_S78_L002.bin.67 | 91.17 | 3.51 | 28.57 | d__Bacteria;p__Firmicutes;c__Bacilli;o__Lactobacillales;f__Streptococcaceae;g__Streptococcus;s__Streptococcus parasuis |
| NGSNJ-086:927:GW2302222891st:3:2212:22327:10629_2:N:0:CTCTATCG+TCAGGCTT_pan_6936_cph-625 | DTU_2020_1007200_1_MG_RL_CPH_Sewage_625_S78_L002.bin.67 | 91.17 | 3.51 | 28.57 | d__Bacteria;p__Firmicutes;c__Bacilli;o__Lactobacillales;f__Streptococcaceae;g__Streptococcus;s__Streptococcus parasuis |
| NGSNJ-086:927:GW2302222891st:3:2204:28302:21198_2:N:0:CTCTATCG+TCAGGCTT_pan_6936_cph-625 | DTU_2020_1007200_1_MG_RL_CPH_Sewage_625_S78_L002.bin.67 | 91.17 | 3.51 | 28.57 | d__Bacteria;p__Firmicutes;c__Bacilli;o__Lactobacillales;f__Streptococcaceae;g__Streptococcus;s__Streptococcus parasuis |
| NGSNJ-086:927:GW2302222891st:3:2170:1958:7545_1:N:0:CTCTATCG+GCAGGCTT_pan_6936_cph-625 | DTU_2020_1007200_1_MG_RL_CPH_Sewage_625_S78_L002.bin.67 | 91.17 | 3.51 | 28.57 | d__Bacteria;p__Firmicutes;c__Bacilli;o__Lactobacillales;f__Streptococcaceae;g__Streptococcus;s__Streptococcus parasuis |
| NGSNJ-086:927:GW2302222891st:3:2168:25816:35837_1:N:0:CTCTATCG+TCAGGCTT_pan_6936_cph-625 | DTU_2020_1007200_1_MG_RL_CPH_Sewage_625_S78_L002.bin.67 | 91.17 | 3.51 | 28.57 | d__Bacteria;p__Firmicutes;c__Bacilli;o__Lactobacillales;f__Streptococcaceae;g__Streptococcus;s__Streptococcus parasuis |
| NGSNJ-086:927:GW2302222891st:3:2168:15944:34867_1:N:0:CTCTATCG+TCAGGCTT_pan_6936_cph-625 | DTU_2020_1007200_1_MG_RL_CPH_Sewage_625_S78_L002.bin.67 | 91.17 | 3.51 | 28.57 | d__Bacteria;p__Firmicutes;c__Bacilli;o__Lactobacillales;f__Streptococcaceae;g__Streptococcus;s__Streptococcus parasuis |
| NGSNJ-086:927:GW2302222891st:3:2163:10077:3443_1:N:0:CTCTATCG+TCAGGCTT_pan_6936_cph-625 | DTU_2020_1007200_1_MG_RL_CPH_Sewage_625_S78_L002.bin.67 | 91.17 | 3.51 | 28.57 | d__Bacteria;p__Firmicutes;c__Bacilli;o__Lactobacillales;f__Streptococcaceae;g__Streptococcus;s__Streptococcus parasuis |
| NGSNJ-086:927:GW2302222891st:3:2162:4472:25770_1:N:0:CTCTATCG+TCAGGCTT_pan_6936_cph-625 | DTU_2020_1007200_1_MG_RL_CPH_Sewage_625_S78_L002.bin.67 | 91.17 | 3.51 | 28.57 | d__Bacteria;p__Firmicutes;c__Bacilli;o__Lactobacillales;f__Streptococcaceae;g__Streptococcus;s__Streptococcus parasuis |
| NGSNJ-086:927:GW2302222891st:3:2158:1705:31031_2:N:0:CTCTATCG+TCAGGCTT_pan_6936_cph-625 | DTU_2020_1007200_1_MG_RL_CPH_Sewage_625_S78_L002.bin.67 | 91.17 | 3.51 | 28.57 | d__Bacteria;p__Firmicutes;c__Bacilli;o__Lactobacillales;f__Streptococcaceae;g__Streptococcus;s__Streptococcus parasuis |
| NGSNJ-086:927:GW2302222891st:3:2157:10013:35524_1:N:0:CTCTATCG+TCAGGCTT_pan_6936_cph-625 | DTU_2020_1007200_1_MG_RL_CPH_Sewage_625_S78_L002.bin.67 | 91.17 | 3.51 | 28.57 | d__Bacteria;p__Firmicutes;c__Bacilli;o__Lactobacillales;f__Streptococcaceae;g__Streptococcus;s__Streptococcus parasuis |
| NGSNJ-086:927:GW2302222891st:3:2150:12789:32346_1:N:0:CTCTATCG+TCAGGCTT_pan_6936_cph-625 | DTU_2020_1007200_1_MG_RL_CPH_Sewage_625_S78_L002.bin.67 | 91.17 | 3.51 | 28.57 | d__Bacteria;p__Firmicutes;c__Bacilli;o__Lactobacillales;f__Streptococcaceae;g__Streptococcus;s__Streptococcus parasuis |
| NGSNJ-086:927:GW2302222891st:3:2141:13187:23390_1:N:0:CTCTATCG+TCAGGCTT_pan_6936_cph-625 | DTU_2020_1007200_1_MG_RL_CPH_Sewage_625_S78_L002.bin.67 | 91.17 | 3.51 | 28.57 | d__Bacteria;p__Firmicutes;c__Bacilli;o__Lactobacillales;f__Streptococcaceae;g__Streptococcus;s__Streptococcus parasuis |
| NGSNJ-086:927:GW2302222891st:3:2118:19307:33833_1:N:0:CTCTATCG+TCAGGCTT_pan_6936_cph-625 | DTU_2020_1007200_1_MG_RL_CPH_Sewage_625_S78_L002.bin.67 | 91.17 | 3.51 | 28.57 | d__Bacteria;p__Firmicutes;c__Bacilli;o__Lactobacillales;f__Streptococcaceae;g__Streptococcus;s__Streptococcus parasuis |
| NGSNJ-086:927:GW2302222891st:3:2118:19434:33739_1:N:0:CTCTATCG+TCAGGCTT_pan_6936_cph-625 | DTU_2020_1007200_1_MG_RL_CPH_Sewage_625_S78_L002.bin.67 | 91.17 | 3.51 | 28.57 | d__Bacteria;p__Firmicutes;c__Bacilli;o__Lactobacillales;f__Streptococcaceae;g__Streptococcus;s__Streptococcus parasuis |
| NGSNJ-086:927:GW2302222891st:3:2118:19416:33708_1:N:0:CTCTATCG+TCAGGCTT_pan_6936_cph-625 | DTU_2020_1007200_1_MG_RL_CPH_Sewage_625_S78_L002.bin.67 | 91.17 | 3.51 | 28.57 | d__Bacteria;p__Firmicutes;c__Bacilli;o__Lactobacillales;f__Streptococcaceae;g__Streptococcus;s__Streptococcus parasuis |
| NGSNJ-086:927:GW2302222891st:3:2118:26648:22717_2:N:0:CTCTATCG+TCAGGCTT_pan_6936_cph-625 | DTU_2020_1007200_1_MG_RL_CPH_Sewage_625_S78_L002.bin.67 | 91.17 | 3.51 | 28.57 | d__Bacteria;p__Firmicutes;c__Bacilli;o__Lactobacillales;f__Streptococcaceae;g__Streptococcus;s__Streptococcus parasuis |
| NGSNJ-086:927:GW2302222891st:3:2116:15374:10019_1:N:0:CTCTATCG+TCAGGCTT_pan_6936_cph-625 | DTU_2020_1007200_1_MG_RL_CPH_Sewage_625_S78_L002.bin.67 | 91.17 | 3.51 | 28.57 | d__Bacteria;p__Firmicutes;c__Bacilli;o__Lactobacillales;f__Streptococcaceae;g__Streptococcus;s__Streptococcus parasuis |
| NGSNJ-086:927:GW2302222891st:3:2110:3152:36041_1:N:0:CTCTATCG+TCAGGCTT_pan_6936_cph-625 | DTU_2020_1007200_1_MG_RL_CPH_Sewage_625_S78_L002.bin.67 | 91.17 | 3.51 | 28.57 | d__Bacteria;p__Firmicutes;c__Bacilli;o__Lactobacillales;f__Streptococcaceae;g__Streptococcus;s__Streptococcus parasuis |
| NGSNJ-086:927:GW2302222891st:3:2105:17806:16861_1:N:0:CTCTATCG+TCAGGCTT_pan_6936_cph-625 | DTU_2020_1007200_1_MG_RL_CPH_Sewage_625_S78_L002.bin.67 | 91.17 | 3.51 | 28.57 | d__Bacteria;p__Firmicutes;c__Bacilli;o__Lactobacillales;f__Streptococcaceae;g__Streptococcus;s__Streptococcus parasuis |
| NGSNJ-086:927:GW2302222891st:3:2105:1208:10567_1:N:0:CTCTATCG+TCAGGCTT_pan_6936_cph-625 | DTU_2020_1007200_1_MG_RL_CPH_Sewage_625_S78_L002.bin.67 | 91.17 | 3.51 | 28.57 | d__Bacteria;p__Firmicutes;c__Bacilli;o__Lactobacillales;f__Streptococcaceae;g__Streptococcus;s__Streptococcus parasuis |
| NGSNJ-086:927:GW2302222891st:3:2104:32651:30921_1:N:0:CTCTATCG+TCAGGCTT_pan_6936_cph-625 | DTU_2020_1007200_1_MG_RL_CPH_Sewage_625_S78_L002.bin.67 | 91.17 | 3.51 | 28.57 | d__Bacteria;p__Firmicutes;c__Bacilli;o__Lactobacillales;f__Streptococcaceae;g__Streptococcus;s__Streptococcus parasuis |
| NGSNJ-086:927:GW2302222891st:3:1668:3486:4773_1:N:0:CTCTATCG+TCAGGCTT_pan_6936_cph-625 | DTU_2020_1007200_1_MG_RL_CPH_Sewage_625_S78_L002.bin.67 | 91.17 | 3.51 | 28.57 | d__Bacteria;p__Firmicutes;c__Bacilli;o__Lactobacillales;f__Streptococcaceae;g__Streptococcus;s__Streptococcus parasuis |
| NGSNJ-086:927:GW2302222891st:3:1661:26223:13589_1:N:0:CTCTATCG+TCAGGCTT_pan_6936_cph-625 | DTU_2020_1007200_1_MG_RL_CPH_Sewage_625_S78_L002.bin.67 | 91.17 | 3.51 | 28.57 | d__Bacteria;p__Firmicutes;c__Bacilli;o__Lactobacillales;f__Streptococcaceae;g__Streptococcus;s__Streptococcus parasuis |
| NGSNJ-086:927:GW2302222891st:3:1647:3360:15107_1:N:0:CTCTATCG+TCAGGCTT_pan_6936_cph-625 | DTU_2020_1007200_1_MG_RL_CPH_Sewage_625_S78_L002.bin.67 | 91.17 | 3.51 | 28.57 | d__Bacteria;p__Firmicutes;c__Bacilli;o__Lactobacillales;f__Streptococcaceae;g__Streptococcus;s__Streptococcus parasuis |
| NGSNJ-086:927:GW2302222891st:3:1647:4092:14904_1:N:0:CTCTATCG+TCAGGCTT_pan_6936_cph-625 | DTU_2020_1007200_1_MG_RL_CPH_Sewage_625_S78_L002.bin.67 | 91.17 | 3.51 | 28.57 | d__Bacteria;p__Firmicutes;c__Bacilli;o__Lactobacillales;f__Streptococcaceae;g__Streptococcus;s__Streptococcus parasuis |
| NGSNJ-086:927:GW2302222891st:3:1635:22589:19038_1:N:0:CTCTATCG+TCAGGCTT_pan_6936_cph-625 | DTU_2020_1007200_1_MG_RL_CPH_Sewage_625_S78_L002.bin.67 | 91.17 | 3.51 | 28.57 | d__Bacteria;p__Firmicutes;c__Bacilli;o__Lactobacillales;f__Streptococcaceae;g__Streptococcus;s__Streptococcus parasuis |
| NGSNJ-086:927:GW2302222891st:3:1620:1877:26506_1:N:0:CTCTATCG+TCAGGCTT_pan_6936_cph-625 | DTU_2020_1007200_1_MG_RL_CPH_Sewage_625_S78_L002.bin.67 | 91.17 | 3.51 | 28.57 | d__Bacteria;p__Firmicutes;c__Bacilli;o__Lactobacillales;f__Streptococcaceae;g__Streptococcus;s__Streptococcus parasuis |
| NGSNJ-086:927:GW2302222891st:3:1608:10601:32440_1:N:0:CTCTATCG+TCAGGCTT_pan_6936_cph-625 | DTU_2020_1007200_1_MG_RL_CPH_Sewage_625_S78_L002.bin.67 | 91.17 | 3.51 | 28.57 | d__Bacteria;p__Firmicutes;c__Bacilli;o__Lactobacillales;f__Streptococcaceae;g__Streptococcus;s__Streptococcus parasuis |
| NGSNJ-086:927:GW2302222891st:3:1551:30897:22842_1:N:0:CTCTATCG+TCAGGCTT_pan_6936_cph-625 | DTU_2020_1007200_1_MG_RL_CPH_Sewage_625_S78_L002.bin.67 | 91.17 | 3.51 | 28.57 | d__Bacteria;p__Firmicutes;c__Bacilli;o__Lactobacillales;f__Streptococcaceae;g__Streptococcus;s__Streptococcus parasuis |
| NGSNJ-086:927:GW2302222891st:3:1551:30798:21731_2:N:0:CTCTATCG+TCAGGCTT_pan_6936_cph-625 | DTU_2020_1007200_1_MG_RL_CPH_Sewage_625_S78_L002.bin.67 | 91.17 | 3.51 | 28.57 | d__Bacteria;p__Firmicutes;c__Bacilli;o__Lactobacillales;f__Streptococcaceae;g__Streptococcus;s__Streptococcus parasuis |
| NGSNJ-086:927:GW2302222891st:3:1540:29206:19476_1:N:0:CTCTATCG+TCAGGCTT_pan_6936_cph-625 | DTU_2020_1007200_1_MG_RL_CPH_Sewage_625_S78_L002.bin.67 | 91.17 | 3.51 | 28.57 | d__Bacteria;p__Firmicutes;c__Bacilli;o__Lactobacillales;f__Streptococcaceae;g__Streptococcus;s__Streptococcus parasuis |
| NGSNJ-086:927:GW2302222891st:3:1531:24343:21324_1:N:0:CTCTATCG+TCAGGCTT_pan_6936_cph-625 | DTU_2020_1007200_1_MG_RL_CPH_Sewage_625_S78_L002.bin.67 | 91.17 | 3.51 | 28.57 | d__Bacteria;p__Firmicutes;c__Bacilli;o__Lactobacillales;f__Streptococcaceae;g__Streptococcus;s__Streptococcus parasuis |
| NGSNJ-086:927:GW2302222891st:3:1530:8775:11209_1:N:0:CTCTATCG+TCAGGCTT_pan_6936_cph-625 | DTU_2020_1007200_1_MG_RL_CPH_Sewage_625_S78_L002.bin.67 | 91.17 | 3.51 | 28.57 | d__Bacteria;p__Firmicutes;c__Bacilli;o__Lactobacillales;f__Streptococcaceae;g__Streptococcus;s__Streptococcus parasuis |
| NGSNJ-086:927:GW2302222891st:3:1462:25265:14935_1:N:0:CTCTATCG+TCAGGCTT_pan_6936_cph-625 | DTU_2020_1007200_1_MG_RL_CPH_Sewage_625_S78_L002.bin.67 | 91.17 | 3.51 | 28.57 | d__Bacteria;p__Firmicutes;c__Bacilli;o__Lactobacillales;f__Streptococcaceae;g__Streptococcus;s__Streptococcus parasuis |
| NGSNJ-086:927:GW2302222891st:3:1405:17508:26334_1:N:0:CTCTATCG+TCAGGCTT_pan_6936_cph-625 | DTU_2020_1007200_1_MG_RL_CPH_Sewage_625_S78_L002.bin.67 | 91.17 | 3.51 | 28.57 | d__Bacteria;p__Firmicutes;c__Bacilli;o__Lactobacillales;f__Streptococcaceae;g__Streptococcus;s__Streptococcus parasuis |
| NGSNJ-086:927:GW2302222891st:3:1354:3766:36229_1:N:0:CTCAATCG+TCAGGCTT_pan_6936_cph-625 | DTU_2020_1007200_1_MG_RL_CPH_Sewage_625_S78_L002.bin.67 | 91.17 | 3.51 | 28.57 | d__Bacteria;p__Firmicutes;c__Bacilli;o__Lactobacillales;f__Streptococcaceae;g__Streptococcus;s__Streptococcus parasuis |
| NGSNJ-086:927:GW2302222891st:3:1312:4544:30123_2:N:0:CTCTATCG+TCAGGCTT_pan_6936_cph-625 | DTU_2020_1007200_1_MG_RL_CPH_Sewage_625_S78_L002.bin.67 | 91.17 | 3.51 | 28.57 | d__Bacteria;p__Firmicutes;c__Bacilli;o__Lactobacillales;f__Streptococcaceae;g__Streptococcus;s__Streptococcus parasuis |
| NGSNJ-086:927:GW2302222891st:3:1306:32262:3505_2:N:0:CTCTATCG+GCAGGCTT_pan_6936_cph-625 | DTU_2020_1007200_1_MG_RL_CPH_Sewage_625_S78_L002.bin.67 | 91.17 | 3.51 | 28.57 | d__Bacteria;p__Firmicutes;c__Bacilli;o__Lactobacillales;f__Streptococcaceae;g__Streptococcus;s__Streptococcus parasuis |
| NGSNJ-086:927:GW2302222891st:3:1266:11080:4429_1:N:0:CTCTATCG+TCAGGCTT_pan_6936_cph-625 | DTU_2020_1007200_1_MG_RL_CPH_Sewage_625_S78_L002.bin.67 | 91.17 | 3.51 | 28.57 | d__Bacteria;p__Firmicutes;c__Bacilli;o__Lactobacillales;f__Streptococcaceae;g__Streptococcus;s__Streptococcus parasuis |
| NGSNJ-086:927:GW2302222891st:3:1242:22462:16908_1:N:0:CTCTATCG+TCAGGCTT_pan_6936_cph-625 | DTU_2020_1007200_1_MG_RL_CPH_Sewage_625_S78_L002.bin.67 | 91.17 | 3.51 | 28.57 | d__Bacteria;p__Firmicutes;c__Bacilli;o__Lactobacillales;f__Streptococcaceae;g__Streptococcus;s__Streptococcus parasuis |
| NGSNJ-086:927:GW2302222891st:3:1229:8820:6120_1:N:0:CTCTATCG+TCAGGCTT_pan_6936_cph-625 | DTU_2020_1007200_1_MG_RL_CPH_Sewage_625_S78_L002.bin.67 | 91.17 | 3.51 | 28.57 | d__Bacteria;p__Firmicutes;c__Bacilli;o__Lactobacillales;f__Streptococcaceae;g__Streptococcus;s__Streptococcus parasuis |
| NGSNJ-086:927:GW2302222891st:3:1229:7789:4993_1:N:0:CTCTATCG+TCAGGCTT_pan_6936_cph-625 | DTU_2020_1007200_1_MG_RL_CPH_Sewage_625_S78_L002.bin.67 | 91.17 | 3.51 | 28.57 | d__Bacteria;p__Firmicutes;c__Bacilli;o__Lactobacillales;f__Streptococcaceae;g__Streptococcus;s__Streptococcus parasuis |
| NGSNJ-086:927:GW2302222891st:3:1210:2311:26757_1:N:0:CTCTATCG+TCAGGCTT_pan_6936_cph-625 | DTU_2020_1007200_1_MG_RL_CPH_Sewage_625_S78_L002.bin.67 | 91.17 | 3.51 | 28.57 | d__Bacteria;p__Firmicutes;c__Bacilli;o__Lactobacillales;f__Streptococcaceae;g__Streptococcus;s__Streptococcus parasuis |
| NGSNJ-086:927:GW2302222891st:3:1209:11288:34757_1:N:0:CTCTATCG+TCAGGCTT_pan_6936_cph-625 | DTU_2020_1007200_1_MG_RL_CPH_Sewage_625_S78_L002.bin.67 | 91.17 | 3.51 | 28.57 | d__Bacteria;p__Firmicutes;c__Bacilli;o__Lactobacillales;f__Streptococcaceae;g__Streptococcus;s__Streptococcus parasuis |
| NGSNJ-086:927:GW2302222891st:3:1205:6524:28541_1:N:0:CTCTATCG+TCAGGCTT_pan_6936_cph-625 | DTU_2020_1007200_1_MG_RL_CPH_Sewage_625_S78_L002.bin.67 | 91.17 | 3.51 | 28.57 | d__Bacteria;p__Firmicutes;c__Bacilli;o__Lactobacillales;f__Streptococcaceae;g__Streptococcus;s__Streptococcus parasuis |
| NGSNJ-086:927:GW2302222891st:3:1205:6515:28494_1:N:0:CTCTATCG+TCAGGCTT_pan_6936_cph-625 | DTU_2020_1007200_1_MG_RL_CPH_Sewage_625_S78_L002.bin.67 | 91.17 | 3.51 | 28.57 | d__Bacteria;p__Firmicutes;c__Bacilli;o__Lactobacillales;f__Streptococcaceae;g__Streptococcus;s__Streptococcus parasuis |
| NGSNJ-086:927:GW2302222891st:3:1175:12518:6981_2:N:0:CTCTATCG+TCAGGCTT_pan_6936_cph-625 | DTU_2020_1007200_1_MG_RL_CPH_Sewage_625_S78_L002.bin.67 | 91.17 | 3.51 | 28.57 | d__Bacteria;p__Firmicutes;c__Bacilli;o__Lactobacillales;f__Streptococcaceae;g__Streptococcus;s__Streptococcus parasuis |
| NGSNJ-086:927:GW2302222891st:3:1168:12255:5713_1:N:0:CTCTATCG+TCAGGCTT_pan_6936_cph-625 | DTU_2020_1007200_1_MG_RL_CPH_Sewage_625_S78_L002.bin.67 | 91.17 | 3.51 | 28.57 | d__Bacteria;p__Firmicutes;c__Bacilli;o__Lactobacillales;f__Streptococcaceae;g__Streptococcus;s__Streptococcus parasuis |
| NGSNJ-086:927:GW2302222891st:3:1132:19045:1094_1:N:0:CTCTATCG+NCAGGCTT_pan_6936_cph-625 | DTU_2020_1007200_1_MG_RL_CPH_Sewage_625_S78_L002.bin.67 | 91.17 | 3.51 | 28.57 | d__Bacteria;p__Firmicutes;c__Bacilli;o__Lactobacillales;f__Streptococcaceae;g__Streptococcus;s__Streptococcus parasuis |
| NGSNJ-086:927:GW2302222891st:3:1126:20311:21480_1:N:0:CTCTATCG+TCAGGCTT_pan_6936_cph-625 | DTU_2020_1007200_1_MG_RL_CPH_Sewage_625_S78_L002.bin.67 | 91.17 | 3.51 | 28.57 | d__Bacteria;p__Firmicutes;c__Bacilli;o__Lactobacillales;f__Streptococcaceae;g__Streptococcus;s__Streptococcus parasuis |
| NGSNJ-086:927:GW2302222891st:3:1125:24080:13448_2:N:0:CTCTATCG+TCAGGCTT_pan_6936_cph-625 | DTU_2020_1007200_1_MG_RL_CPH_Sewage_625_S78_L002.bin.67 | 91.17 | 3.51 | 28.57 | d__Bacteria;p__Firmicutes;c__Bacilli;o__Lactobacillales;f__Streptococcaceae;g__Streptococcus;s__Streptococcus parasuis |
| NGSNJ-086:927:GW2302222891st:3:1122:21884:25050_1:N:0:CTCTATCG+TCAGGCTT_pan_6936_cph-625 | DTU_2020_1007200_1_MG_RL_CPH_Sewage_625_S78_L002.bin.67 | 91.17 | 3.51 | 28.57 | d__Bacteria;p__Firmicutes;c__Bacilli;o__Lactobacillales;f__Streptococcaceae;g__Streptococcus;s__Streptococcus parasuis |
| NGSNJ-086:927:GW2302222891st:3:1114:8305:34444_1:N:0:CTCTATCG+TCAGGCTT_pan_6936_cph-625 | DTU_2020_1007200_1_MG_RL_CPH_Sewage_625_S78_L002.bin.67 | 91.17 | 3.51 | 28.57 | d__Bacteria;p__Firmicutes;c__Bacilli;o__Lactobacillales;f__Streptococcaceae;g__Streptococcus;s__Streptococcus parasuis |
| NGSNJ-086:927:GW2302222891st:3:1114:32461:29559_1:N:0:CTCTATCG+TCAGGCTT_pan_6936_cph-625 | DTU_2020_1007200_1_MG_RL_CPH_Sewage_625_S78_L002.bin.67 | 91.17 | 3.51 | 28.57 | d__Bacteria;p__Firmicutes;c__Bacilli;o__Lactobacillales;f__Streptococcaceae;g__Streptococcus;s__Streptococcus parasuis |
| NGSNJ-086:927:GW2302222891st:3:1109:32271:1799_2:N:0:CTCTATCG+TAAGGCTT_pan_6936_cph-625 | DTU_2020_1007200_1_MG_RL_CPH_Sewage_625_S78_L002.bin.67 | 91.17 | 3.51 | 28.57 | d__Bacteria;p__Firmicutes;c__Bacilli;o__Lactobacillales;f__Streptococcaceae;g__Streptococcus;s__Streptococcus parasuis |
| NGSNJ-086:927:GW2302222891st:3:1105:2483:10802_1:N:0:CTCTATCG+TCAGGCTT_pan_6936_cph-625 | DTU_2020_1007200_1_MG_RL_CPH_Sewage_625_S78_L002.bin.67 | 91.17 | 3.51 | 28.57 | d__Bacteria;p__Firmicutes;c__Bacilli;o__Lactobacillales;f__Streptococcaceae;g__Streptococcus;s__Streptococcus parasuis |
| NGSNJ-086:927:GW2302222891st:3:2650:28049:1376_1:N:0:CCACATTG+AAGTGTCG_pan_6936_cph-642 | DTU_2020_1007216_2_MG_RL_CPH_Sewage_642_S101_L003.bin.10 | 96.08 | 4.95 | 27.27 | d__Bacteria;p__Firmicutes;c__Bacilli;o__Lactobacillales;f__Streptococcaceae;g__Streptococcus;s__Streptococcus parasuis |
| NGSNJ-086:927:GW2302222891st:3:2632:15492:5588_1:N:0:CCACATTG+AAGTGTCG_pan_6936_cph-642 | DTU_2020_1007216_2_MG_RL_CPH_Sewage_642_S101_L003.bin.10 | 96.08 | 4.95 | 27.27 | d__Bacteria;p__Firmicutes;c__Bacilli;o__Lactobacillales;f__Streptococcaceae;g__Streptococcus;s__Streptococcus parasuis |
| NGSNJ-086:927:GW2302222891st:3:2620:9182:15483_1:N:0:CCACATTG+ACGTGTCG_pan_6936_cph-642 | DTU_2020_1007216_2_MG_RL_CPH_Sewage_642_S101_L003.bin.10 | 96.08 | 4.95 | 27.27 | d__Bacteria;p__Firmicutes;c__Bacilli;o__Lactobacillales;f__Streptococcaceae;g__Streptococcus;s__Streptococcus parasuis |
| NGSNJ-086:927:GW2302222891st:3:2620:10122:15045_1:N:0:CCACATTG+AAGTGTCG_pan_6936_cph-642 | DTU_2020_1007216_2_MG_RL_CPH_Sewage_642_S101_L003.bin.10 | 96.08 | 4.95 | 27.27 | d__Bacteria;p__Firmicutes;c__Bacilli;o__Lactobacillales;f__Streptococcaceae;g__Streptococcus;s__Streptococcus parasuis |
| NGSNJ-086:927:GW2302222891st:3:2619:29568:25269_1:N:0:CCACATTG+AAGTGTCG_pan_6936_cph-642 | DTU_2020_1007216_2_MG_RL_CPH_Sewage_642_S101_L003.bin.10 | 96.08 | 4.95 | 27.27 | d__Bacteria;p__Firmicutes;c__Bacilli;o__Lactobacillales;f__Streptococcaceae;g__Streptococcus;s__Streptococcus parasuis |
| NGSNJ-086:927:GW2302222891st:3:2612:21875:26036_2:N:0:CCACATTG+AAGTGTCG_pan_6936_cph-642 | DTU_2020_1007216_2_MG_RL_CPH_Sewage_642_S101_L003.bin.10 | 96.08 | 4.95 | 27.27 | d__Bacteria;p__Firmicutes;c__Bacilli;o__Lactobacillales;f__Streptococcaceae;g__Streptococcus;s__Streptococcus parasuis |
| NGSNJ-086:927:GW2302222891st:3:2607:24560:16814_1:N:0:CCACATTG+AAGTGTCG_pan_6936_cph-642 | DTU_2020_1007216_2_MG_RL_CPH_Sewage_642_S101_L003.bin.10 | 96.08 | 4.95 | 27.27 | d__Bacteria;p__Firmicutes;c__Bacilli;o__Lactobacillales;f__Streptococcaceae;g__Streptococcus;s__Streptococcus parasuis |
| NGSNJ-086:927:GW2302222891st:3:2604:31069:35822_1:N:0:CCACATTG+AAGTGTCG_pan_6936_cph-642 | DTU_2020_1007216_2_MG_RL_CPH_Sewage_642_S101_L003.bin.10 | 96.08 | 4.95 | 27.27 | d__Bacteria;p__Firmicutes;c__Bacilli;o__Lactobacillales;f__Streptococcaceae;g__Streptococcus;s__Streptococcus parasuis |
| NGSNJ-086:927:GW2302222891st:3:2604:18602:13448_1:N:0:CCACATTG+AAGTGTCG_pan_6936_cph-642 | DTU_2020_1007216_2_MG_RL_CPH_Sewage_642_S101_L003.bin.10 | 96.08 | 4.95 | 27.27 | d__Bacteria;p__Firmicutes;c__Bacilli;o__Lactobacillales;f__Streptococcaceae;g__Streptococcus;s__Streptococcus parasuis |
| NGSNJ-086:927:GW2302222891st:3:2604:23891:4977_1:N:0:CCACATTG+ACGTGTCG_pan_6936_cph-642 | DTU_2020_1007216_2_MG_RL_CPH_Sewage_642_S101_L003.bin.10 | 96.08 | 4.95 | 27.27 | d__Bacteria;p__Firmicutes;c__Bacilli;o__Lactobacillales;f__Streptococcaceae;g__Streptococcus;s__Streptococcus parasuis |
| NGSNJ-086:927:GW2302222891st:3:2576:8901:28025_1:N:0:CCACATTG+AAGTGTCG_pan_6936_cph-642 | DTU_2020_1007216_2_MG_RL_CPH_Sewage_642_S101_L003.bin.10 | 96.08 | 4.95 | 27.27 | d__Bacteria;p__Firmicutes;c__Bacilli;o__Lactobacillales;f__Streptococcaceae;g__Streptococcus;s__Streptococcus parasuis |
| NGSNJ-086:927:GW2302222891st:3:2560:27498:5682_2:N:0:CCACATTG+AAGTGTCG_pan_6936_cph-642 | DTU_2020_1007216_2_MG_RL_CPH_Sewage_642_S101_L003.bin.10 | 96.08 | 4.95 | 27.27 | d__Bacteria;p__Firmicutes;c__Bacilli;o__Lactobacillales;f__Streptococcaceae;g__Streptococcus;s__Streptococcus parasuis |
| NGSNJ-086:927:GW2302222891st:3:2546:21169:34710_2:N:0:CCACATTG+AAGTGTCG_pan_6936_cph-642 | DTU_2020_1007216_2_MG_RL_CPH_Sewage_642_S101_L003.bin.10 | 96.08 | 4.95 | 27.27 | d__Bacteria;p__Firmicutes;c__Bacilli;o__Lactobacillales;f__Streptococcaceae;g__Streptococcus;s__Streptococcus parasuis |
| NGSNJ-086:927:GW2302222891st:3:2501:13612:2644_1:N:0:CCACATTG+AAGTGTCG_pan_6936_cph-642 | DTU_2020_1007216_2_MG_RL_CPH_Sewage_642_S101_L003.bin.10 | 96.08 | 4.95 | 27.27 | d__Bacteria;p__Firmicutes;c__Bacilli;o__Lactobacillales;f__Streptococcaceae;g__Streptococcus;s__Streptococcus parasuis |
| NGSNJ-086:927:GW2302222891st:3:2463:6153:3537_1:N:0:CCACATTG+AAGTGTCG_pan_6936_cph-642 | DTU_2020_1007216_2_MG_RL_CPH_Sewage_642_S101_L003.bin.10 | 96.08 | 4.95 | 27.27 | d__Bacteria;p__Firmicutes;c__Bacilli;o__Lactobacillales;f__Streptococcaceae;g__Streptococcus;s__Streptococcus parasuis |
| NGSNJ-086:927:GW2302222891st:3:2416:27181:32221_2:N:0:CCACATTG+AAGTGTCG_pan_6936_cph-642 | DTU_2020_1007216_2_MG_RL_CPH_Sewage_642_S101_L003.bin.10 | 96.08 | 4.95 | 27.27 | d__Bacteria;p__Firmicutes;c__Bacilli;o__Lactobacillales;f__Streptococcaceae;g__Streptococcus;s__Streptococcus parasuis |
| NGSNJ-086:927:GW2302222891st:3:2316:20265:25410_1:N:0:CCACATTG+AAGTGTCG_pan_6936_cph-642 | DTU_2020_1007216_2_MG_RL_CPH_Sewage_642_S101_L003.bin.10 | 96.08 | 4.95 | 27.27 | d__Bacteria;p__Firmicutes;c__Bacilli;o__Lactobacillales;f__Streptococcaceae;g__Streptococcus;s__Streptococcus parasuis |
| NGSNJ-086:927:GW2302222891st:3:2315:21594:4820_1:N:0:CCACATTG+AAGTGTCG_pan_6936_cph-642 | DTU_2020_1007216_2_MG_RL_CPH_Sewage_642_S101_L003.bin.10 | 96.08 | 4.95 | 27.27 | d__Bacteria;p__Firmicutes;c__Bacilli;o__Lactobacillales;f__Streptococcaceae;g__Streptococcus;s__Streptococcus parasuis |
| NGSNJ-086:927:GW2302222891st:3:2259:19913:6417_1:N:0:CCACATTG+AAGTGTCG_pan_6936_cph-642 | DTU_2020_1007216_2_MG_RL_CPH_Sewage_642_S101_L003.bin.10 | 96.08 | 4.95 | 27.27 | d__Bacteria;p__Firmicutes;c__Bacilli;o__Lactobacillales;f__Streptococcaceae;g__Streptococcus;s__Streptococcus parasuis |
| NGSNJ-086:927:GW2302222891st:3:2243:24551:19398_1:N:0:CCACATTG+AAGTGTCG_pan_6936_cph-642 | DTU_2020_1007216_2_MG_RL_CPH_Sewage_642_S101_L003.bin.10 | 96.08 | 4.95 | 27.27 | d__Bacteria;p__Firmicutes;c__Bacilli;o__Lactobacillales;f__Streptococcaceae;g__Streptococcus;s__Streptococcus parasuis |
| NGSNJ-086:927:GW2302222891st:3:2225:9670:17926_1:N:0:CCACATTG+AAGTGTCG_pan_6936_cph-642 | DTU_2020_1007216_2_MG_RL_CPH_Sewage_642_S101_L003.bin.10 | 96.08 | 4.95 | 27.27 | d__Bacteria;p__Firmicutes;c__Bacilli;o__Lactobacillales;f__Streptococcaceae;g__Streptococcus;s__Streptococcus parasuis |
| NGSNJ-086:927:GW2302222891st:3:2207:30861:18552_1:N:0:CCACATTG+AAGTGTCG_pan_6936_cph-642 | DTU_2020_1007216_2_MG_RL_CPH_Sewage_642_S101_L003.bin.10 | 96.08 | 4.95 | 27.27 | d__Bacteria;p__Firmicutes;c__Bacilli;o__Lactobacillales;f__Streptococcaceae;g__Streptococcus;s__Streptococcus parasuis |
| NGSNJ-086:927:GW2302222891st:3:2205:32307:12978_1:N:0:CCACATTG+AAGTGTCG_pan_6936_cph-642 | DTU_2020_1007216_2_MG_RL_CPH_Sewage_642_S101_L003.bin.10 | 96.08 | 4.95 | 27.27 | d__Bacteria;p__Firmicutes;c__Bacilli;o__Lactobacillales;f__Streptococcaceae;g__Streptococcus;s__Streptococcus parasuis |
| NGSNJ-086:927:GW2302222891st:3:2175:31403:21652_2:N:0:CCACATTG+AAGTGTCG_pan_6936_cph-642 | DTU_2020_1007216_2_MG_RL_CPH_Sewage_642_S101_L003.bin.10 | 96.08 | 4.95 | 27.27 | d__Bacteria;p__Firmicutes;c__Bacilli;o__Lactobacillales;f__Streptococcaceae;g__Streptococcus;s__Streptococcus parasuis |
| NGSNJ-086:927:GW2302222891st:3:2172:19551:3505_1:N:0:CCACATTG+AAGTGTAG_pan_6936_cph-642 | DTU_2020_1007216_2_MG_RL_CPH_Sewage_642_S101_L003.bin.10 | 96.08 | 4.95 | 27.27 | d__Bacteria;p__Firmicutes;c__Bacilli;o__Lactobacillales;f__Streptococcaceae;g__Streptococcus;s__Streptococcus parasuis |
| NGSNJ-086:927:GW2302222891st:3:2170:20555:34303_1:N:0:CCACATTG+AAGTGTCG_pan_6936_cph-642 | DTU_2020_1007216_2_MG_RL_CPH_Sewage_642_S101_L003.bin.10 | 96.08 | 4.95 | 27.27 | d__Bacteria;p__Firmicutes;c__Bacilli;o__Lactobacillales;f__Streptococcaceae;g__Streptococcus;s__Streptococcus parasuis |
| NGSNJ-086:927:GW2302222891st:3:2163:16062:2973_1:N:0:CCACATTG+CAGTGTCG_pan_6936_cph-642 | DTU_2020_1007216_2_MG_RL_CPH_Sewage_642_S101_L003.bin.10 | 96.08 | 4.95 | 27.27 | d__Bacteria;p__Firmicutes;c__Bacilli;o__Lactobacillales;f__Streptococcaceae;g__Streptococcus;s__Streptococcus parasuis |
| NGSNJ-086:927:GW2302222891st:3:2155:13621:24048_1:N:0:CCACATTG+AAGTGTCG_pan_6936_cph-642 | DTU_2020_1007216_2_MG_RL_CPH_Sewage_642_S101_L003.bin.10 | 96.08 | 4.95 | 27.27 | d__Bacteria;p__Firmicutes;c__Bacilli;o__Lactobacillales;f__Streptococcaceae;g__Streptococcus;s__Streptococcus parasuis |
| NGSNJ-086:927:GW2302222891st:3:2146:14705:4946_1:N:0:CCACATTG+AAGTGTCG_pan_6936_cph-642 | DTU_2020_1007216_2_MG_RL_CPH_Sewage_642_S101_L003.bin.10 | 96.08 | 4.95 | 27.27 | d__Bacteria;p__Firmicutes;c__Bacilli;o__Lactobacillales;f__Streptococcaceae;g__Streptococcus;s__Streptococcus parasuis |
| NGSNJ-086:927:GW2302222891st:3:2145:2022:6684_1:N:0:CCACATTG+AAGTGTCG_pan_6936_cph-642 | DTU_2020_1007216_2_MG_RL_CPH_Sewage_642_S101_L003.bin.10 | 96.08 | 4.95 | 27.27 | d__Bacteria;p__Firmicutes;c__Bacilli;o__Lactobacillales;f__Streptococcaceae;g__Streptococcus;s__Streptococcus parasuis |
| NGSNJ-086:927:GW2302222891st:3:2144:21061:34334_1:N:0:CCACATTG+ACGTGTCG_pan_6936_cph-642 | DTU_2020_1007216_2_MG_RL_CPH_Sewage_642_S101_L003.bin.10 | 96.08 | 4.95 | 27.27 | d__Bacteria;p__Firmicutes;c__Bacilli;o__Lactobacillales;f__Streptococcaceae;g__Streptococcus;s__Streptococcus parasuis |
| NGSNJ-086:927:GW2302222891st:3:2144:1271:6950_1:N:0:CCACATTG+AAGTGTCG_pan_6936_cph-642 | DTU_2020_1007216_2_MG_RL_CPH_Sewage_642_S101_L003.bin.10 | 96.08 | 4.95 | 27.27 | d__Bacteria;p__Firmicutes;c__Bacilli;o__Lactobacillales;f__Streptococcaceae;g__Streptococcus;s__Streptococcus parasuis |
| NGSNJ-086:927:GW2302222891st:3:2134:26323:5619_1:N:0:CCACATTG+AAGTGTCG_pan_6936_cph-642 | DTU_2020_1007216_2_MG_RL_CPH_Sewage_642_S101_L003.bin.10 | 96.08 | 4.95 | 27.27 | d__Bacteria;p__Firmicutes;c__Bacilli;o__Lactobacillales;f__Streptococcaceae;g__Streptococcus;s__Streptococcus parasuis |
| NGSNJ-086:927:GW2302222891st:3:2127:15284:12117_1:N:0:CCACATTG+AAGTGTCG_pan_6936_cph-642 | DTU_2020_1007216_2_MG_RL_CPH_Sewage_642_S101_L003.bin.10 | 96.08 | 4.95 | 27.27 | d__Bacteria;p__Firmicutes;c__Bacilli;o__Lactobacillales;f__Streptococcaceae;g__Streptococcus;s__Streptococcus parasuis |
| NGSNJ-086:927:GW2302222891st:3:2118:24496:15452_1:N:0:CCACATTG+AAGTGTCG_pan_6936_cph-642 | DTU_2020_1007216_2_MG_RL_CPH_Sewage_642_S101_L003.bin.10 | 96.08 | 4.95 | 27.27 | d__Bacteria;p__Firmicutes;c__Bacilli;o__Lactobacillales;f__Streptococcaceae;g__Streptococcus;s__Streptococcus parasuis |
| NGSNJ-086:927:GW2302222891st:3:2113:6099:4257_2:N:0:CCACATTG+AAGTGTCG_pan_6936_cph-642 | DTU_2020_1007216_2_MG_RL_CPH_Sewage_642_S101_L003.bin.10 | 96.08 | 4.95 | 27.27 | d__Bacteria;p__Firmicutes;c__Bacilli;o__Lactobacillales;f__Streptococcaceae;g__Streptococcus;s__Streptococcus parasuis |
| NGSNJ-086:927:GW2302222891st:3:2113:6234:4053_2:N:0:CCACATTG+AAGTGTCG_pan_6936_cph-642 | DTU_2020_1007216_2_MG_RL_CPH_Sewage_642_S101_L003.bin.10 | 96.08 | 4.95 | 27.27 | d__Bacteria;p__Firmicutes;c__Bacilli;o__Lactobacillales;f__Streptococcaceae;g__Streptococcus;s__Streptococcus parasuis |
| NGSNJ-086:927:GW2302222891st:3:2103:12599:36307_1:N:0:CCACATTG+ACGTGTCG_pan_6936_cph-642 | DTU_2020_1007216_2_MG_RL_CPH_Sewage_642_S101_L003.bin.10 | 96.08 | 4.95 | 27.27 | d__Bacteria;p__Firmicutes;c__Bacilli;o__Lactobacillales;f__Streptococcaceae;g__Streptococcus;s__Streptococcus parasuis |
| NGSNJ-086:927:GW2302222891st:3:1676:12066:21574_1:N:0:CCACATTG+AAGTGTCG_pan_6936_cph-642 | DTU_2020_1007216_2_MG_RL_CPH_Sewage_642_S101_L003.bin.10 | 96.08 | 4.95 | 27.27 | d__Bacteria;p__Firmicutes;c__Bacilli;o__Lactobacillales;f__Streptococcaceae;g__Streptococcus;s__Streptococcus parasuis |
| NGSNJ-086:927:GW2302222891st:3:1671:5005:28166_1:N:0:CCACATTG+AAGTGTCG_pan_6936_cph-642 | DTU_2020_1007216_2_MG_RL_CPH_Sewage_642_S101_L003.bin.10 | 96.08 | 4.95 | 27.27 | d__Bacteria;p__Firmicutes;c__Bacilli;o__Lactobacillales;f__Streptococcaceae;g__Streptococcus;s__Streptococcus parasuis |
| NGSNJ-086:927:GW2302222891st:3:1661:22652:28761_2:N:0:CCACATTG+AAGTGTCG_pan_6936_cph-642 | DTU_2020_1007216_2_MG_RL_CPH_Sewage_642_S101_L003.bin.10 | 96.08 | 4.95 | 27.27 | d__Bacteria;p__Firmicutes;c__Bacilli;o__Lactobacillales;f__Streptococcaceae;g__Streptococcus;s__Streptococcus parasuis |
| NGSNJ-086:927:GW2302222891st:3:1658:16604:1219_1:N:0:CCACATTG+AAGTGTCG_pan_6936_cph-642 | DTU_2020_1007216_2_MG_RL_CPH_Sewage_642_S101_L003.bin.10 | 96.08 | 4.95 | 27.27 | d__Bacteria;p__Firmicutes;c__Bacilli;o__Lactobacillales;f__Streptococcaceae;g__Streptococcus;s__Streptococcus parasuis |
| NGSNJ-086:927:GW2302222891st:3:1656:28935:13745_1:N:0:CCACATTG+AAGTGTCG_pan_6936_cph-642 | DTU_2020_1007216_2_MG_RL_CPH_Sewage_642_S101_L003.bin.10 | 96.08 | 4.95 | 27.27 | d__Bacteria;p__Firmicutes;c__Bacilli;o__Lactobacillales;f__Streptococcaceae;g__Streptococcus;s__Streptococcus parasuis |
| NGSNJ-086:927:GW2302222891st:3:1646:25934:10019_1:N:0:CCACATTG+AAGTGTCG_pan_6936_cph-642 | DTU_2020_1007216_2_MG_RL_CPH_Sewage_642_S101_L003.bin.10 | 96.08 | 4.95 | 27.27 | d__Bacteria;p__Firmicutes;c__Bacilli;o__Lactobacillales;f__Streptococcaceae;g__Streptococcus;s__Streptococcus parasuis |
| NGSNJ-086:927:GW2302222891st:3:1634:23095:17472_1:N:0:CCACATTG+AAGTGTCG_pan_6936_cph-642 | DTU_2020_1007216_2_MG_RL_CPH_Sewage_642_S101_L003.bin.10 | 96.08 | 4.95 | 27.27 | d__Bacteria;p__Firmicutes;c__Bacilli;o__Lactobacillales;f__Streptococcaceae;g__Streptococcus;s__Streptococcus parasuis |
| NGSNJ-086:927:GW2302222891st:3:1631:25997:27727_2:N:0:CCACATTG+AAGTGTCG_pan_6936_cph-642 | DTU_2020_1007216_2_MG_RL_CPH_Sewage_642_S101_L003.bin.10 | 96.08 | 4.95 | 27.27 | d__Bacteria;p__Firmicutes;c__Bacilli;o__Lactobacillales;f__Streptococcaceae;g__Streptococcus;s__Streptococcus parasuis |
| NGSNJ-086:927:GW2302222891st:3:1602:28230:24455_1:N:0:CCACATTG+AAGTGTCG_pan_6936_cph-642 | DTU_2020_1007216_2_MG_RL_CPH_Sewage_642_S101_L003.bin.10 | 96.08 | 4.95 | 27.27 | d__Bacteria;p__Firmicutes;c__Bacilli;o__Lactobacillales;f__Streptococcaceae;g__Streptococcus;s__Streptococcus parasuis |
| NGSNJ-086:927:GW2302222891st:3:1602:7446:14356_2:N:0:CCACATTG+AAGTGTCG_pan_6936_cph-642 | DTU_2020_1007216_2_MG_RL_CPH_Sewage_642_S101_L003.bin.10 | 96.08 | 4.95 | 27.27 | d__Bacteria;p__Firmicutes;c__Bacilli;o__Lactobacillales;f__Streptococcaceae;g__Streptococcus;s__Streptococcus parasuis |
| NGSNJ-086:927:GW2302222891st:3:1601:21730:16391_1:N:0:CCACATTG+AAGTGTCG_pan_6936_cph-642 | DTU_2020_1007216_2_MG_RL_CPH_Sewage_642_S101_L003.bin.10 | 96.08 | 4.95 | 27.27 | d__Bacteria;p__Firmicutes;c__Bacilli;o__Lactobacillales;f__Streptococcaceae;g__Streptococcus;s__Streptococcus parasuis |
| NGSNJ-086:927:GW2302222891st:3:1568:32117:5541_1:N:0:CCACATTG+AAGTGTCG_pan_6936_cph-642 | DTU_2020_1007216_2_MG_RL_CPH_Sewage_642_S101_L003.bin.10 | 96.08 | 4.95 | 27.27 | d__Bacteria;p__Firmicutes;c__Bacilli;o__Lactobacillales;f__Streptococcaceae;g__Streptococcus;s__Streptococcus parasuis |
| NGSNJ-086:927:GW2302222891st:3:1538:27371:25535_1:N:0:CCACATTG+AAGTGTCG_pan_6936_cph-642 | DTU_2020_1007216_2_MG_RL_CPH_Sewage_642_S101_L003.bin.10 | 96.08 | 4.95 | 27.27 | d__Bacteria;p__Firmicutes;c__Bacilli;o__Lactobacillales;f__Streptococcaceae;g__Streptococcus;s__Streptococcus parasuis |
| NGSNJ-086:927:GW2302222891st:3:1529:2248:25676_1:N:0:CCACATTG+AAGTGTCG_pan_6936_cph-642 | DTU_2020_1007216_2_MG_RL_CPH_Sewage_642_S101_L003.bin.10 | 96.08 | 4.95 | 27.27 | d__Bacteria;p__Firmicutes;c__Bacilli;o__Lactobacillales;f__Streptococcaceae;g__Streptococcus;s__Streptococcus parasuis |
| NGSNJ-086:927:GW2302222891st:3:1524:12500:21825_1:N:0:CCACATTG+AAGTGTCG_pan_6936_cph-642 | DTU_2020_1007216_2_MG_RL_CPH_Sewage_642_S101_L003.bin.10 | 96.08 | 4.95 | 27.27 | d__Bacteria;p__Firmicutes;c__Bacilli;o__Lactobacillales;f__Streptococcaceae;g__Streptococcus;s__Streptococcus parasuis |
| NGSNJ-086:927:GW2302222891st:3:1519:30978:2065_1:N:0:CCACATTG+AAGTGTCG_pan_6936_cph-642 | DTU_2020_1007216_2_MG_RL_CPH_Sewage_642_S101_L003.bin.10 | 96.08 | 4.95 | 27.27 | d__Bacteria;p__Firmicutes;c__Bacilli;o__Lactobacillales;f__Streptococcaceae;g__Streptococcus;s__Streptococcus parasuis |
| NGSNJ-086:927:GW2302222891st:3:1512:7627:23218_1:N:0:CCACATTG+AAGTGTCC_pan_6936_cph-642 | DTU_2020_1007216_2_MG_RL_CPH_Sewage_642_S101_L003.bin.10 | 96.08 | 4.95 | 27.27 | d__Bacteria;p__Firmicutes;c__Bacilli;o__Lactobacillales;f__Streptococcaceae;g__Streptococcus;s__Streptococcus parasuis |
| NGSNJ-086:927:GW2302222891st:3:1501:32027:34350_1:N:0:CCACATTG+AAGTGTCG_pan_6936_cph-642 | DTU_2020_1007216_2_MG_RL_CPH_Sewage_642_S101_L003.bin.10 | 96.08 | 4.95 | 27.27 | d__Bacteria;p__Firmicutes;c__Bacilli;o__Lactobacillales;f__Streptococcaceae;g__Streptococcus;s__Streptococcus parasuis |
| NGSNJ-086:927:GW2302222891st:3:1453:19768:17284_1:N:0:CCACATTG+AAGTGTCG_pan_6936_cph-642 | DTU_2020_1007216_2_MG_RL_CPH_Sewage_642_S101_L003.bin.10 | 96.08 | 4.95 | 27.27 | d__Bacteria;p__Firmicutes;c__Bacilli;o__Lactobacillales;f__Streptococcaceae;g__Streptococcus;s__Streptococcus parasuis |
| NGSNJ-086:927:GW2302222891st:3:1439:32344:29042_1:N:0:CCACATTG+AAGTGTCG_pan_6936_cph-642 | DTU_2020_1007216_2_MG_RL_CPH_Sewage_642_S101_L003.bin.10 | 96.08 | 4.95 | 27.27 | d__Bacteria;p__Firmicutes;c__Bacilli;o__Lactobacillales;f__Streptococcaceae;g__Streptococcus;s__Streptococcus parasuis |
| NGSNJ-086:927:GW2302222891st:3:1437:30581:34319_2:N:0:CCACATTG+AAGTGTCG_pan_6936_cph-642 | DTU_2020_1007216_2_MG_RL_CPH_Sewage_642_S101_L003.bin.10 | 96.08 | 4.95 | 27.27 | d__Bacteria;p__Firmicutes;c__Bacilli;o__Lactobacillales;f__Streptococcaceae;g__Streptococcus;s__Streptococcus parasuis |
| NGSNJ-086:927:GW2302222891st:3:1408:7943:7639_1:N:0:CCACATTG+AAGTTTCG_pan_6936_cph-642 | DTU_2020_1007216_2_MG_RL_CPH_Sewage_642_S101_L003.bin.10 | 96.08 | 4.95 | 27.27 | d__Bacteria;p__Firmicutes;c__Bacilli;o__Lactobacillales;f__Streptococcaceae;g__Streptococcus;s__Streptococcus parasuis |
| NGSNJ-086:927:GW2302222891st:3:1408:7717:6965_1:N:0:CCACATTG+AAGTGTCG_pan_6936_cph-642 | DTU_2020_1007216_2_MG_RL_CPH_Sewage_642_S101_L003.bin.10 | 96.08 | 4.95 | 27.27 | d__Bacteria;p__Firmicutes;c__Bacilli;o__Lactobacillales;f__Streptococcaceae;g__Streptococcus;s__Streptococcus parasuis |
| NGSNJ-086:927:GW2302222891st:3:1406:13883:35712_2:N:0:CCACATTG+AAGTGTCG_pan_6936_cph-642 | DTU_2020_1007216_2_MG_RL_CPH_Sewage_642_S101_L003.bin.10 | 96.08 | 4.95 | 27.27 | d__Bacteria;p__Firmicutes;c__Bacilli;o__Lactobacillales;f__Streptococcaceae;g__Streptococcus;s__Streptococcus parasuis |
| NGSNJ-086:927:GW2302222891st:3:1406:7310:9048_1:N:0:CCACATTG+AAGTGTCG_pan_6936_cph-642 | DTU_2020_1007216_2_MG_RL_CPH_Sewage_642_S101_L003.bin.10 | 96.08 | 4.95 | 27.27 | d__Bacteria;p__Firmicutes;c__Bacilli;o__Lactobacillales;f__Streptococcaceae;g__Streptococcus;s__Streptococcus parasuis |
| NGSNJ-086:927:GW2302222891st:3:1378:32868:25316_1:N:0:CCACATTG+AAGTGTCG_pan_6936_cph-642 | DTU_2020_1007216_2_MG_RL_CPH_Sewage_642_S101_L003.bin.10 | 96.08 | 4.95 | 27.27 | d__Bacteria;p__Firmicutes;c__Bacilli;o__Lactobacillales;f__Streptococcaceae;g__Streptococcus;s__Streptococcus parasuis |
| NGSNJ-086:927:GW2302222891st:3:1377:21260:5619_1:N:0:CCACATTG+AAGTGTCG_pan_6936_cph-642 | DTU_2020_1007216_2_MG_RL_CPH_Sewage_642_S101_L003.bin.10 | 96.08 | 4.95 | 27.27 | d__Bacteria;p__Firmicutes;c__Bacilli;o__Lactobacillales;f__Streptococcaceae;g__Streptococcus;s__Streptococcus parasuis |
| NGSNJ-086:927:GW2302222891st:3:1359:2248:17065_2:N:0:CCACATTG+AAGTGTCG_pan_6936_cph-642 | DTU_2020_1007216_2_MG_RL_CPH_Sewage_642_S101_L003.bin.10 | 96.08 | 4.95 | 27.27 | d__Bacteria;p__Firmicutes;c__Bacilli;o__Lactobacillales;f__Streptococcaceae;g__Streptococcus;s__Streptococcus parasuis |
| NGSNJ-086:927:GW2302222891st:3:1350:8865:12242_2:N:0:CCACATTG+AAGTGTCG_pan_6936_cph-642 | DTU_2020_1007216_2_MG_RL_CPH_Sewage_642_S101_L003.bin.10 | 96.08 | 4.95 | 27.27 | d__Bacteria;p__Firmicutes;c__Bacilli;o__Lactobacillales;f__Streptococcaceae;g__Streptococcus;s__Streptococcus parasuis |
| NGSNJ-086:927:GW2302222891st:3:1273:17942:22451_1:N:0:CCACAATG+AAGTGTCG_pan_6936_cph-642 | DTU_2020_1007216_2_MG_RL_CPH_Sewage_642_S101_L003.bin.10 | 96.08 | 4.95 | 27.27 | d__Bacteria;p__Firmicutes;c__Bacilli;o__Lactobacillales;f__Streptococcaceae;g__Streptococcus;s__Streptococcus parasuis |
| NGSNJ-086:927:GW2302222891st:3:1256:8079:13009_1:N:0:CCACATTG+AAGGGTCG_pan_6936_cph-642 | DTU_2020_1007216_2_MG_RL_CPH_Sewage_642_S101_L003.bin.10 | 96.08 | 4.95 | 27.27 | d__Bacteria;p__Firmicutes;c__Bacilli;o__Lactobacillales;f__Streptococcaceae;g__Streptococcus;s__Streptococcus parasuis |
| NGSNJ-086:927:GW2302222891st:3:1241:28510:15671_2:N:0:CCACATTG+AAGTGTCG_pan_6936_cph-642 | DTU_2020_1007216_2_MG_RL_CPH_Sewage_642_S101_L003.bin.10 | 96.08 | 4.95 | 27.27 | d__Bacteria;p__Firmicutes;c__Bacilli;o__Lactobacillales;f__Streptococcaceae;g__Streptococcus;s__Streptococcus parasuis |
| NGSNJ-086:927:GW2302222891st:3:1210:2166:4586_2:N:0:CCACATTG+AAGTGTCG_pan_6936_cph-642 | DTU_2020_1007216_2_MG_RL_CPH_Sewage_642_S101_L003.bin.10 | 96.08 | 4.95 | 27.27 | d__Bacteria;p__Firmicutes;c__Bacilli;o__Lactobacillales;f__Streptococcaceae;g__Streptococcus;s__Streptococcus parasuis |
| NGSNJ-086:927:GW2302222891st:3:1207:30770:11882_1:N:0:CCACATTG+AAGTGTCG_pan_6936_cph-642 | DTU_2020_1007216_2_MG_RL_CPH_Sewage_642_S101_L003.bin.10 | 96.08 | 4.95 | 27.27 | d__Bacteria;p__Firmicutes;c__Bacilli;o__Lactobacillales;f__Streptococcaceae;g__Streptococcus;s__Streptococcus parasuis |
| NGSNJ-086:927:GW2302222891st:3:1202:29686:18615_1:N:0:CCACATTG+AAGTGTCG_pan_6936_cph-642 | DTU_2020_1007216_2_MG_RL_CPH_Sewage_642_S101_L003.bin.10 | 96.08 | 4.95 | 27.27 | d__Bacteria;p__Firmicutes;c__Bacilli;o__Lactobacillales;f__Streptococcaceae;g__Streptococcus;s__Streptococcus parasuis |
| NGSNJ-086:927:GW2302222891st:3:1202:29677:18599_1:N:0:CCACATTG+AAGTGTCG_pan_6936_cph-642 | DTU_2020_1007216_2_MG_RL_CPH_Sewage_642_S101_L003.bin.10 | 96.08 | 4.95 | 27.27 | d__Bacteria;p__Firmicutes;c__Bacilli;o__Lactobacillales;f__Streptococcaceae;g__Streptococcus;s__Streptococcus parasuis |
| NGSNJ-086:927:GW2302222891st:3:1164:23267:12352_1:N:0:CCACATTG+AAGTGTCG_pan_6936_cph-642 | DTU_2020_1007216_2_MG_RL_CPH_Sewage_642_S101_L003.bin.10 | 96.08 | 4.95 | 27.27 | d__Bacteria;p__Firmicutes;c__Bacilli;o__Lactobacillales;f__Streptococcaceae;g__Streptococcus;s__Streptococcus parasuis |
| NGSNJ-086:927:GW2302222891st:3:1151:32452:32769_1:N:0:CCACATTG+AAGTGTCG_pan_6936_cph-642 | DTU_2020_1007216_2_MG_RL_CPH_Sewage_642_S101_L003.bin.10 | 96.08 | 4.95 | 27.27 | d__Bacteria;p__Firmicutes;c__Bacilli;o__Lactobacillales;f__Streptococcaceae;g__Streptococcus;s__Streptococcus parasuis |
| NGSNJ-086:927:GW2302222891st:3:1129:14995:15969_2:N:0:CCACATTG+AAGTGTCG_pan_6936_cph-642 | DTU_2020_1007216_2_MG_RL_CPH_Sewage_642_S101_L003.bin.10 | 96.08 | 4.95 | 27.27 | d__Bacteria;p__Firmicutes;c__Bacilli;o__Lactobacillales;f__Streptococcaceae;g__Streptococcus;s__Streptococcus parasuis |

**Supplementary table 13***:* Plasmid identification and characterization of Hi-C assembled plasmids containing *vanHAX* and *vanHBX* resistance genes.

| **Contig** | **Contig length** | **ARGs (ResFinder)** | **Genes (Platon)** | **ARGs (Platon)** | **Plasmid annotation (Platon)** | **Plasmid annotation (ProxiMeta)** |
| --- | --- | --- | --- | --- | --- | --- |
| pan_6936_resfinder\|vanhax_2_m97297_k141_4125729_cph-662 | 9082 | vanHAX | A0A073K5I5 Resolvase  Q06240 Sensor protein VanS  Q05709 D-specific alpha-keto acid dehydrogenase  A0A0F6V063 D-alanine--D-alanine ligase  Q06241 D-alanyl-D-alanine dipeptidase | vanA  vanH  vanR  vanS  vanX | NC_008768.1 Enterococcus faecium plasmid pVEF1 | NC_008821.1  Enterococcus faecium plasmid pVEF2 |
| pan_6936_resfinder\|vanhax_2_m97297_k141_2262417_cph-683 | 9082 | vanHAX | A0A073K5I5 Resolvase  Q05709 D-specific alpha-keto acid dehydrogenase  A0A0F6V063 D-alanine--D-alanine ligase  Q06241 D-alanyl-D-alanine dipeptidase | vanA  vanH   vanR  vanS  vanX | NC_008768.1 Enterococcus faecium plasmid pVEF1 | NC_008821.1  Enterococcus faecium plasmid pVEF2 |
| pan_6936_resfinder\|vanhax_2_m97297_k141_11877556_cph-689 | 1189 | vanHAX | Q05709 D-specific alpha-keto acid dehydrogenase | vanH | NC_008768.1  Enterococcus faecium plasmid pVEF1 | NC_014959.1    Enterococcus faecium plasmid pS177 |
| pan_6936_resfinder\|vanhax_2_m97297_k141_2183710_cph-625 | 9083 | vanHAX | A0A073K5I5 Resolvase  Q06240 Sensor protein VanS  Q05709 D-specific alpha-keto acid dehydrogenase  A0A0F6V063 D-alanine--D-alanine ligase  Q06241 D-alanyl-D-alanine dipeptidase | vanA  vanH  vanR  vanS  vanX | NC_008768.1 Enterococcus faecium plasmid pVEF1 | NC_008821.1  Enterococcus faecium plasmid pVEF2 |
| pan_6936_resfinder\|vanhax_2_m97297_k141_504333_cph-631 | 11304 | vanHAX | A0A073K5I5 Resolvase  Q06240 Sensor protein VanS  Q05709 D-specific alpha-keto acid dehydrogenase  A0A0F6V063 D-alanine--D-alanine ligase   Q06241 D-alanyl-D-alanine dipeptidase  UPI00032D8ABD VanY-A/VanY-F/VanY-M family D-Ala-D-Ala carboxypeptidase  Q06242 Protein VanZ | vanA  vanH  vanR  vanS  vanX  vanY  vanZ-A | NC_008768.1  Enterococcus faecium plasmid pVEF1 | NC_008821.1  Enterococcus faecium plasmid pVEF2 |
| pan_6936_resfinder\|vanhax_2_m97297_k141_6986355_cph-689 | 4034 | vanHAX | Q06241 D-alanyl-D-alanine dipeptidase  UPI00032D8ABD VanY-A/VanY-F/VanY-M family D-Ala-D-Ala carboxypeptidase  Q06242 Protein VanZ | vanA  vanX  vanY  vanZ-A | NC_016967.1  Enterococcus faecium plasmid pZB18 | NZ_CP059757.1  Enterococcus faecium strain A10290 plasmid pA10290_P2 |
| pan_6936_resfinder\|vanhax_2_m97297_k141_2346236_cph-617 | 12205 | vanHAX | A0A073K5I5 Resolvase  Q06240 Sensor protein VanS  Q05709 D-specific alpha-keto acid dehydrogenase  A0A0F6V063 D-alanine--D-alanine ligase  Q06241 D-alanyl-D-alanine dipeptidase  UPI00032D8ABD VanY-A/VanY-F/VanY-M family D-Ala-D-Ala carboxypeptidase  Q06242 Protein VanZ | vanA  vanH  vanR  vanS  vanX  vanY  vanZ-A | NC_016967.1  Enterococcus faecium plasmid pZB18 | NC_005054.1  Staphylococcus aureus plasmid pLW043 |
| pan_6936_resfinder\|vanhax_2_m97297_k141_589123_cph-710 | 9440 | vanHAX | A0A073K5I5 Resolvase  Q06240 Sensor protein VanS  Q05709 D-specific alpha-keto acid dehydrogenase  A0A0F6V063 D-alanine--D-alanine ligase  Q06241 D-alanyl-D-alanine dipeptidase  UPI00032D8ABD VanY-A/VanY-F/VanY-M family D-Ala-D-Ala carboxypeptidase  Q06242 Protein VanZ | vanA  vanH  vanR  vanS  vanX  vanY  vanZ-A | NC_016967.1  Enterococcus faecium plasmid pZB18 | NC_016967.1  Enterococcus faecium plasmid pZB18 |
| pan_6936_resfinder\|vanhax_2_m97297_k141_1755641_cph-654 | 4937 | vanHAX | Q05709 D-specific alpha-keto acid dehydrogenase  A0A0F6V063 D-alanine--D-alanine ligase  Q06241 D-alanyl-D-alanine dipeptidase  UPI00032D8ABD VanY-A/VanY-F/VanY-M family D-Ala-D-Ala carboxypeptidase  Q06242 Protein VanZ | vanA  vanH  vanX  vanY  vanZ-A | NZ_CP043327.1  Enterococcus durans strain VREdu plasmid pSULI | NZ_CP040238.1  Enterococcus faecium strain VB3025 plasmid unnamed2  (record removed by RefSeq staff) |
| pan_6936_resfinder\|vanhax_2_m97297_k141_2038509_cph-654 | 6259 | vanHAX | A0A073K5I5 Resolvase  Q06240 Sensor protein VanS | vanR  vanS | NC_005054.1  Staphylococcus aureus plasmid pLW043 | NC_005054.1  Staphylococcus aureus plasmid pLW043 |
| pan_7222_resfinder\|vanhbx_1_af192329_k141_10858702_cph-689 | 11784 | vanHBX | Q47744 Regulatory protein VanRB  Q47745 Sensor protein VanSB  A0A1A7T3C1 VanY protein, D-alanyl-D-alanine carboxypeptidase  Q47748 D-specific alpha-keto acid dehydrogenase  Q47749 D-alanyl-D-alanine dipeptidase  J7QXS5 Uncharacterized protein  N2AXX1 Excisionase  J7Q5L1 Integrase | vanA  vanH  vanR  vanS  vanX | NZ_LR135359.1  Enterococcus faecium isolate E7948 plasmid 3 | NZ_LR135359.1  Enterococcus faecium isolate E7948 plasmid 3 |
| pan_7222_resfinder\|vanhbx_1_af192329_k141_3106163_cph-683 | 8311 | vanHBX | Q9L8Y6 HTH cro/C1-type domain-containing protein  Q47744 Regulatory protein VanRB  Q47745 Sensor protein VanSB  A0A1A7T3C1 VanY protein, D-alanyl-D-alanine carboxypeptidase  Q47748 D-specific alpha-keto acid dehydrogenase  Q47749 D-alanyl-D-alanine dipeptidase | vanA  vanH  vanR  vanS  vanX | NZ_LR135359.1  Enterococcus faecium isolate E7948 plasmid 3 | NZ_KR066794.1  Enterococcus faecium strain Efm0123 plasmid pJEG050 |
| pan_7222_resfinder\|vanhbx_1_af192329_k141_3597014_cph-662 | 3583 | vanHBX | Q47748 D-specific alpha-keto acid dehydrogenase  Q47749 D-alanyl-D-alanine dipeptidase | vanA  vanH  vanX | NC_011642.1  Enterococcus faecalis plasmid pMG2200 | NZ_KR066794.1  Enterococcus faecium strain Efm0123 plasmid pJEG050 |
| pan_7222_resfinder\|vanhbx_1_af192329_k141_967247_cph-640 | 1027 | vanHBX |  |  | NC_011642.1  Enterococcus faecalis plasmid pMG2200 | NC_011642.1  Enterococcus faecalis plasmid pMG2200 |
| pan_7222_resfinder\|vanhbx_1_af192329_k141_2592145_cph-640 | 4801 | vanHBX | Q47748 D-specific alpha-keto acid dehydrogenase  Q47749 D-alanyl-D-alanine dipeptidase  J7QXS5 Uncharacterized protein  N2AXX1 Excisionase | vanA  vanX | NC_011642.1  Enterococcus faecalis plasmid pMG2200 | NZ_KR066794.1  Enterococcus faecium strain Efm0123 plasmid pJEG050 |
| pan_7222_resfinder\|vanhbx_1_af192329_k141_410380_cph-654 | 1938 | vanHBX |  | vanA | NC_011642.1  Enterococcus faecalis plasmid pMG2200 | NZ_KR066794.1  Enterococcus faecium strain Efm0123 plasmid pJEG050 |
| pan_7222_resfinder\|vanhbx_1_af192329_k141_2199614_cph-617 | 4310 | vanHBX | Q47748 D-specific alpha-keto acid dehydrogenase  Q47749 D-alanyl-D-alanine dipeptidase  J7QXS5 Uncharacterized protein | vanA  vanH  vanX | NC_011642.1  Enterococcus faecalis plasmid pMG2200 | NZ_KR066794.1  Enterococcus faecium strain Efm0123 plasmid pJEG050 |

**Supplementary table 14***:* Plasmid identification and characterization of Hi-C assembled contigs containing *vanHAX* and *vanHBX* resistance genes.

| **Sample** | **Contig** | **Contig length** | **ARG (ResFinder)** | **Genes (Platon)** | **ARGs (Platon)** | **Plasmid (Platon)** |
| --- | --- | --- | --- | --- | --- | --- |
| cph-617 | k141_2346236 | 12205 | vanHAX | Q06242 Protein VanZ  UPI00032D8ABD VanY-A/VanY-F/VanY-M family D-Ala-D-Ala carboxypeptidase  Q06241 D-alanyl-D-alanine dipeptidase  A0A0F6V063 D-alanine--D-alanine ligase  Q05709 D-specific alpha-keto acid dehydrogenase  E0X5A7 VanS  A0A073K5I5 Resolvase | vanA  vanH  vanR  vanS  vanX  vanY  vanZ-A | NC_016967.1 Enterococcus faecium plasmid pZB18, complete sequence |
| cph-625 | k141_2183710 | 9083 | vanHAX | A0A073K5I5 Resolvase  E0X5A7 VanS  Q05709 D-specific alpha-keto acid dehydrogenase  A0A0F6V063 D-alanine--D-alanine ligase  Q06241 D-alanyl-D-alanine dipeptidase | vanA  vanH  vanR  vanS  vanX | NC_008768.1 Enterococcus faecium plasmid pVEF1, complete sequence |
| cph-625 | k141_2317423 | 2586 | vanHAX |  |  | Not a plasmid |
| cph-625 | k141_2339302 | 278 | vanHAX |  |  | Not a plasmid |
| cph-640 | k141_1870923 | 15281 | vanHAX | Q06242 Protein VanZ  UPI00032D8ABD VanY-A/VanY-F/VanY-M family D-Ala-D-Ala carboxypeptidase  Q06241 D-alanyl-D-alanine dipeptidase  A0A0F6V063 D-alanine--D-alanine ligase  Q05709 D-specific alpha-keto acid dehydrogenase  E0X5A7 VanS  A0A073K5I5 Resolvase | vanA  vanH  vanR  vanS  vanX  vanY  vanZ-A | Not a plasmid |
| cph-649 | k141_1914074 | 12372 | vanHAX | A0A073K5I5 Resolvase  Q06240 Sensor protein VanS  Q05709 D-specific alpha-keto acid dehydrogenase  A0A0F6V063 D-alanine--D-alanine ligase  Q06241 D-alanyl-D-alanine dipeptidase | vanA  vanH  vanR  vanS  vanX | Not a plasmid |
| cph-662 | k141_4125729 | 9082 | vanHAX | A0A073K5I5 Resolvase  E0X5A7 VanS  Q05709 D-specific alpha-keto acid dehydrogenase  A0A0F6V063 D-alanine--D-alanine ligase  Q06241 D-alanyl-D-alanine dipeptidase | vanA  vanH  vanR  vanS  vanX | NC_008768.1 Enterococcus faecium plasmid pVEF1, complete sequence |
| cph-689 | k141_6986355 | 4034 | vanHAX | Q06241 D-alanyl-D-alanine dipeptidase  UPI00032D8ABD VanY-A/VanY-F/VanY-M family D-Ala-D-Ala carboxypeptidase  Q06242 Protein VanZ | vanA  vanX  vanY  vanZ-A | NC_016967.1 Enterococcus faecium plasmid pZB18, complete sequence |
| cph-689 | k141_11877556 | 1189 | vanHAX | Q05709 D-specific alpha-keto acid dehydrogenase | vanH | NC_008768.1 Enterococcus faecium plasmid pVEF1, complete sequence |
| cph-623 | k141_2113570 | 15297 | vanHAX | A0A073K5I5 Resolvase  E0X5A7 VanS  Q05709 D-specific alpha-keto acid dehydrogenase  A0A0F6V063 D-alanine--D-alanine ligase  Q06241 D-alanyl-D-alanine dipeptidase  UPI00032D8ABD VanY-A/VanY-F/VanY-M family D-Ala-D-Ala carboxypeptidase  Q06242 Protein VanZ | vanA  vanH  vanR  vanS  vanX  vanY  vanZ-A | Not a plasmid |
| cph-631 | k141_504333 | 11304 | vanHAX | Q06242 Protein VanZ  UPI00032D8ABD VanY-A/VanY-F/VanY-M family D-Ala-D-Ala carboxypeptidase  Q06241 D-alanyl-D-alanine dipeptidase  A0A0F6V063 D-alanine--D-alanine ligase  Q05709 D-specific alpha-keto acid dehydrogenase  E0X5A7 VanS  A0A073K5I5 Resolvase | vanA  vanH  vanR  vanS  vanX  vanY  vanZ-A | NC_008768.1 Enterococcus faecium plasmid pVEF1, complete sequence |
| cph-642 | k141_625701 | 10065 | VanHAX | Q05709 D-specific alpha-keto acid dehydrogenase  A0A0F6V063 D-alanine--D-alanine ligase  Q06241 D-alanyl-D-alanine dipeptidase  UPI00032D8ABD VanY-A/VanY-F/VanY-M family D-Ala-D-Ala carboxypeptidase  Q06242 Protein VanZ | vanA  vanH  vanX  vanY  vanZ-A | Not a plasmid |
| cph-642 | k141_1111964 | 10933 | VanHAX | A0A073K5I5 Resolvase  Q06240 Sensor protein VanS | vanR  vanS | Not a plasmid |
| cph-642 | k141_1571987 | 1253 | VanHAX |  |  | Not a plasmid |
| cph-654 | k141_2038509 | 6259 | VanHAX | A0A073K5I5 Resolvase  E0X5A7 VanS | vanR  vanS | NC_005054.1 Staphylococcus aureus plasmid pLW043 |
| cph-654 | k141_1755641 | 4937 | VanHAX | Q05709 D-specific alpha-keto acid dehydrogenase  A0A0F6V063 D-alanine--D-alanine ligase  Q06241 D-alanyl-D-alanine dipeptidase  UPI00032D8ABD VanY-A/VanY-F/VanY-M family D-Ala-D-Ala carboxypeptidase  Q06242 Protein VanZ | vanA  vanH  vanX  vanY  vanZ-A | NZ_CP043327.1 Enterococcus durans strain VREdu plasmid pSULI |
| cph-654 | k141_194960 | 1449 | VanHAX |  |  | Not a plasmid |
| cph-683 | k141_1466881 | 4382 | VanHAX |  |  | Not a plasmid |
| cph-683 | k141_2262417 | 9082 | VanHAX | A0A073K5I5 Resolvase  E0X5A7 VanS  Q05709 D-specific alpha-keto acid dehydrogenase  A0A0F6V063 D-alanine--D-alanine ligase  Q06241 D-alanyl-D-alanine dipeptidase | vanA  vanH  vanR  vanS  vanX | NC_008768.1 Enterococcus faecium plasmid pVEF1, complete sequence |
| cph-710 | k141_589123 | 9440 | VanHAX | Q06242 Protein VanZ  UPI00032D8ABD VanY-A/VanY-F/VanY-M family D-Ala-D-Ala carboxypeptidase  Q06241 D-alanyl-D-alanine dipeptidase  A0A0F6V063 D-alanine--D-alanine ligase  Q05709 D-specific alpha-keto acid dehydrogenase  Q06240 Sensor protein VanS  A0A073K5I5 Resolvase | vanA  vanH  vanR  vanS  vanX  vanY  vanZ-A | NC_016967.1 Enterococcus faecium plasmid pZB18, complete sequence |
| cph-617 | k141_2199614 | 4310 | vanHBX | B6ZHJ7 HTH_16 domain-containing protein  Q714P2 Uncharacterized protein  Q47749 D-alanyl-D-alanine dipeptidase  Q47748 D-specific alpha-keto acid dehydrogenase | vanA  vanH  vanX | NC_011642.1 Enterococcus faecalis plasmid pMG2200, complete sequence |
| cph-625 | k141_57541 | 619 | vanHBX |  |  | NC_011642.1 Enterococcus faecalis plasmid pMG2200 |
| cph-625 | k141_121600 | 310 | vanHBX |  |  | NZ_LR135359.1 Enterococcus faecium isolate E7948 plasmid 3 |
| cph-625 | k141_169250 | 306 | vanHBX |  |  | NC_011642.1 Enterococcus faecalis plasmid pMG2200 |
| cph-625 | k141_624811 | 1060 | vanHBX |  |  | NC_011642.1 Enterococcus faecalis plasmid pMG2200 |
| cph-640 | k141_2592145 | 4801 | vanHBX | Q47748 D-specific alpha-keto acid dehydrogenase  Q47749 D-alanyl-D-alanine dipeptidase  Q714P2 Uncharacterized protein  B6ZHJ7 HTH_16 domain-containing protein  N2AXX1 Excisionase | vanA  vanX | NC_011642.1 Enterococcus faecalis plasmid pMG2200 |
| cph-649 | k141_2595780 | 467 | vanHBX |  |  | NC_011642.1 Enterococcus faecalis plasmid pMG2200 |
| cph-649 | k141_1043235 | 593 | vanHBX |  |  | NC_011642.1 Enterococcus faecalis plasmid pMG2200 |
| cph-649 | k141_343717 | 435 | vanHBX |  |  | NZ_LR135359.1 Enterococcus faecium isolate E7948 plasmid 3 |
| cph-662 | k141_3597014 | 3583 | vanHBX | Q47747 Vancomycin B-type resistance protein VanW  Q47748 D-specific alpha-keto acid dehydrogenase  Q47749 D-alanyl-D-alanine dipeptidase | vanA  vanH  vanW  vanX | NC_011642.1 Enterococcus faecalis plasmid pMG2200 |
| cph-689 | k141_10858702 | 11784 | vanHBX | J7Q5L1 Integrase  N2AXX1 Excisionase  B6ZHJ7 HTH_16 domain-containing protein  Q714P2 Uncharacterized protein  Q47749 D-alanyl-D-alanine dipeptidase  Q47748 D-specific alpha-keto acid dehydrogenase  Q47747 Vancomycin B-type resistance protein VanW  A0A1A7T3C1 VanY protein, D-alanyl-D-alanine carboxypeptidase  Q47745 Sensor protein VanSB  Q47744 Regulatory protein VanRB | vanA  vanH  vanR  vanS  vanW  vanX  vanY | NZ_LR135359.1 Enterococcus faecium isolate E7948 plasmid 3 |
| cph-623 | k141_277946 | 311 | vanHBX |  |  | NC_011642.1 Enterococcus faecalis plasmid pMG2200 |
| cph-631 | k141_929121 | 317 | vanHBX |  |  | NC_011642.1 Enterococcus faecalis plasmid pMG2200 |
| cph-631 | k141_692737 | 445 | vanHBX |  |  | NC_011642.1 Enterococcus faecalis plasmid pMG2200 |
| cph-642 | k141_1447104 | 561 | vanHBX |  |  | NZ_LR135359.1 Enterococcus faecium isolate E7948 plasmid 3 |
| cph-642 | k141_1871856 | 378 | vanHBX |  |  | NC_011642.1 Enterococcus faecalis plasmid pMG2200 |
| cph-642 | k141_483720 | 386 | vanHBX |  |  | NC_011642.1 Enterococcus faecalis plasmid pMG2200 |
| cph-642 | k141_3116245 | 686 | vanHBX |  |  | NC_011642.1 Enterococcus faecalis plasmid pMG2200 |
| cph-654 | k141_610568 | 403 | vanHBX |  |  | NZ_LR135359.1 Enterococcus faecium isolate E7948 plasmid 3 |
| cph-654 | k141_410380 | 1938 | vanHBX |  |  | NC_011642.1 Enterococcus faecalis plasmid pMG2200 |
| cph-683 | k141_3106163 | 8311 | vanHBX | Q9L8Y6 HTH cro/C1-type domain-containing protein  J7QJY3 Transposase  Q47744 Regulatory protein VanRB  Q47745 Sensor protein VanSB  A0A1A7T3C1 VanY protein, D-alanyl-D-alanine carboxypeptidase  Q47747 Vancomycin B-type resistance protein VanW  Q47748 D-specific alpha-keto acid dehydrogenase  Q47749 D-alanyl-D-alanine dipeptidase | vanA  vanH  vanR  vanS  vanW  vanX  vanY | NZ_LR135359.1 Enterococcus faecium isolate E7948 plasmid 3 |
| cph-710 | k141_54845 | 1197 | vanHBX |  | vanA | NC_011642.1 Enterococcus faecalis plasmid pMG2200 |

**Supplementary table 15***:* Plasmid identification and characterization of conventional contigs containing *vanHAX* and *vanHBX* resistance genes.

| **Contig** | **Contig length** | **ARG (ResFinder)** | **Genes (Platon)** | **ARGs (Platon)** | **Plasmid (Platon)** |
| --- | --- | --- | --- | --- | --- |
| DTU_2020_1007158_1_MG_RL_CPH_Sewage_518_S19_L001_contig=k141.1302651_flag=0_coverage=20.9198_length=11118 | 11118 | vanHAX | A0A073K5I5 Resolvase  E0X5A7 VanS  Q05709 D-specific alpha-keto acid dehydrogenase  A0A0F6V063 D-alanine--D-alanine ligase  Q06241 D-alanyl-D-alanine dipeptidase  UPI00032D8ABD VanY-A/VanY-F/VanY-M family D-Ala-D-Ala carboxypeptidase  Q06242 Protein VanZ | vanA  vanH  vanR  vanS  vanX  vanY  vanZ-A | NC_019213.1 Enterococcus faecalis plasmid pWZ909 |
| DTU_2020_1007176_1_MG_RL_CPH_Sewage_597_S23_L001_contig=k141.88194_flag=1_coverage=4.0000_length=1270 | 1270 | vanHAX | A0A0F6V063 D-alanine--D-alanine ligase | vanA | NC_008768.1 Enterococcus faecium plasmid pVEF1 |
| DTU_2020_1007180_2_MG_RL_CPH_Sewage_602_S60_L002_contig=k141.755715_flag=1_coverage=6.0000_length=3062 | 3062 | vanHAX | A0A0F6V063 D-alanine--D-alanine ligase  Q05709 D-specific alpha-keto acid dehydrogenase | vanA  vanH | NC_008768.1 Enterococcus faecium plasmid pVEF2 |
| DTU_2020_1007183_2_MG_RL_CPH_Sewage_605_S5_L001_contig=k141.1475009_flag=0_coverage=10.9653_length=6477 | 6477 | vanHAX | A0A073K5I5 Resolvase  E0X5A7 VanS | vanR  vanS | NC_005054.1 Staphylococcus aureus plasmid pLW043 |
| DTU_2020_1007183_2_MG_RL_CPH_Sewage_605_S5_L001_contig=k141.220326_flag=0_coverage=10.5869_length=6750 | 6750 | vanHAX | Q05709 D-specific alpha-keto acid dehydrogenase  A0A0F6V063 D-alanine--D-alanine ligase  Q06241 D-alanyl-D-alanine dipeptidase  UPI00032D8ABD VanY-A/VanY-F/VanY-M family D-Ala-D-Ala carboxypeptidase  Q06242 Protein VanZ | vanA  vanH  vanX  vanY  vanZ-A | Not a plasmid |
| DTU_2020_1007186_2_MG_RL_CPH_Sewage_608_S27_L001_contig=k141.304762_flag=1_coverage=9.1216_length=4162 | 4162 | vanHAX | Q05709 D-specific alpha-keto acid dehydrogenase  A0A0F6V063 D-alanine--D-alanine ligase  Q06241 D-alanyl-D-alanine dipeptidase | vanA  vanH  vanX | NC_008768.1 Enterococcus faecium plasmid pVEF1 |
| DTU_2020_1007189_1_MG_RL_CPH_Sewage_614_S73_L002_contig=k141.996307_flag=0_coverage=25.9388_length=9337 | 9337 | vanHAX | A0A073K5I5 Resolvase  E0X5A7 VanS  Q05709 D-specific alpha-keto acid dehydrogenase  A0A0F6V063 D-alanine--D-alanine ligase  Q06241 D-alanyl-D-alanine dipeptidase | vanA  vanH  vanR  vanS  vanX | NC_005054.1 Staphylococcus aureus plasmid pLW043 |
| DTU_2020_1007192_1_MG_RL_CPH_Sewage_617_S74_L002_contig=k141.969396_flag=0_coverage=12.0000_length=8899 | 8899 | vanHAX | A0A073K5I5 Resolvase  E0X5A7 VanS  Q05709 D-specific alpha-keto acid dehydrogenase  A0A0F6V063 D-alanine--D-alanine ligase  Q06241 D-alanyl-D-alanine dipeptidase | vanA  vanH  vanR  vanS  vanX | NC_016967.1 Enterococcus faecium plasmid pZB18 |
| DTU_2020_1007195_1_MG_RL_CPH_Sewage_620_S32_L001_contig=k141.357718_flag=0_coverage=11.9463_length=9146 | 9146 | vanHAX | A0A073K5I5 Resolvase  E0X5A7 VanS  Q05709 D-specific alpha-keto acid dehydrogenase  A0A0F6V063 D-alanine--D-alanine ligase  Q06241 D-alanyl-D-alanine dipeptidase | vanA  vanH  vanR  vanS  vanX | NC_008768.1 Enterococcus faecium plasmid pVEF1 |
| DTU_2020_1007200_1_MG_RL_CPH_Sewage_625_S78_L002_contig=k141.998976_flag=0_coverage=27.2334_length=11620 | 11620 | vanHAX | A0A073K5I5 Resolvase  E0X5A7 VanS  Q05709 D-specific alpha-keto acid dehydrogenase  A0A0F6V063 D-alanine--D-alanine ligase  Q06241 D-alanyl-D-alanine dipeptidase  UPI00032D8ABD VanY-A/VanY-F/VanY-M family D-Ala-D-Ala carboxypeptidase  Q06242 Protein VanZ | vanA  vanH  vanR  vanS  vanX  vanY  vanZ-A | NC_016967.1 Enterococcus faecium plasmid pZB18 |
| DTU_2020_1007200_1_MG_RL_CPH_Sewage_625_S78_L002_contig=k141.1853072_flag=0_coverage=30.4378_length=1315 | 1315 | vanHAX |  |  | NC_022602.1 Carnobacterium inhibens subsp. gilichinskyi plasmid pWNCR47 |
| DTU_2020_1007203_1_MG_RL_CPH_Sewage_628_S35_L001_contig=k141.1545277_flag=0_coverage=3.9122_length=1644 | 1644 | vanHAX |  |  | Not a plasmid |
| DTU_2020_1007203_1_MG_RL_CPH_Sewage_628_S35_L001_contig=k141.472694_flag=0_coverage=12.5386_length=8996 | 8996 | vanHAX | Q06242 Protein VanZ  UPI00032D8ABD VanY-A/VanY-F/VanY-M family D-Ala-D-Ala carboxypeptidase  Q06241 D-alanyl-D-alanine dipeptidase  A0A0F6V063 D-alanine--D-alanine ligase  Q05709 D-specific alpha-keto acid dehydrogenase | vanA  vanH  vanX  vanY  vanZ-A | Not a plasmid |
| DTU_2020_1007203_1_MG_RL_CPH_Sewage_628_S35_L001_contig=k141.1054120_flag=0_coverage=10.9633_length=6136 | 6136 | vanHAX | A0A073K5I5 Resolvase  E0X5A7 VanS | vanR  vanS | NC_016967.1 Enterococcus faecium plasmid pZB18 |
| DTU_2020_1007206_1_MG_RL_CPH_Sewage_631_S36_L001_contig=k141.731722_flag=1_coverage=6.0000_length=5312 | 5312 | vanHAX | Q06240 Sensor protein VanS  Q05709 D-specific alpha-keto acid dehydrogenase  A0A0F6V063 D-alanine--D-alanine ligase  Q06241 D-alanyl-D-alanine dipeptidase | vanA  vanH  vanS  vanX | NC_008768.1 Enterococcus faecium plasmid pVEF1 |
| DTU_2020_1007209_1_MG_RL_CPH_Sewage_634_S81_L002_contig=k141.97176_flag=1_coverage=20.9523_length=9793 | 9793 | vanHAX | Q06241 D-alanyl-D-alanine dipeptidase  A0A0F6V063 D-alanine--D-alanine ligase  Q05709 D-specific alpha-keto acid dehydrogenase  E0X5A7 VanS  A0A073K5I5 Resolvase | vanA  vanH  vanR  vanS  vanX | NC_005054.1 Staphylococcus aureus plasmid pLW043 |
| DTU_2020_1007216_1_MG_RL_CPH_Sewage_642_S10_L001_contig=k141.598620_flag=0_coverage=19.0291_length=7700 | 7700 | vanHAX | Q06242 Protein VanZ  UPI00032D8ABD VanY-A/VanY-F/VanY-M family D-Ala-D-Ala carboxypeptidase  Q06241 D-alanyl-D-alanine dipeptidase  A0A0F6V063 D-alanine--D-alanine ligase  Q05709 D-specific alpha-keto acid dehydrogenase | vanA  vanH  vanX  vanY  vanZ-A | Not a plasmid |
| DTU_2020_1007216_1_MG_RL_CPH_Sewage_642_S10_L001_contig=k141.551381_flag=0_coverage=5.9738_length=4343 | 4343 | vanHAX |  |  | Not a plasmid |
| DTU_2020_1007216_1_MG_RL_CPH_Sewage_642_S10_L001_contig=k141.589603_flag=0_coverage=19.8588_length=6196 | 6196 | vanHAX | A0A073K5I5 Resolvase  E0X5A7 VanS | vanR  vanS | NC_008768.1 Enterococcus faecium plasmid pVEF1 |
| DTU_2020_1007216_2_MG_RL_CPH_Sewage_642_S101_L003_contig=k141.147418_flag=1_coverage=9.0000_length=11514 | 11514 | vanHAX | A0A073K5I5 Resolvase  E0X5A7 VanS  Q05709 D-specific alpha-keto acid dehydrogenase  A0A0F6V063 D-alanine--D-alanine ligase  Q06241 D-alanyl-D-alanine dipeptidase  UPI00032D8ABD VanY-A/VanY-F/VanY-M family D-Ala-D-Ala carboxypeptidase  Q06242 Protein VanZ | vanA  vanH  vanR  vanS  vanX  vanY  vanZ-A | NC_016967.1 Enterococcus faecium plasmid pZB18 |
| DTU_2020_1007219_1_MG_RL_CPH_Sewage_646_S11_L001_contig=k141.834467_flag=0_coverage=20.1061_length=9073 | 9073 | vanHAX | A0A073K5I5 Resolvase  E0X5A7 VanS  Q05709 D-specific alpha-keto acid dehydrogenase  A0A0F6V063 D-alanine--D-alanine ligase  Q06241 D-alanyl-D-alanine dipeptidase | vanA  vanH  vanR  vanS  vanX | NZ_CP043327.1 Enterococcus durans strain VREdu plasmid pSULI |
| DTU_2020_1007219_2_MG_RL_CPH_Sewage_646_S155_L004_contig=k141.893480_flag=0_coverage=18.9337_length=6114 | 6114 | vanHAX | A0A073K5I5 Resolvase  E0X5A7 VanS | vanR  vanS | NZ_LR135346.1 Enterococcus faecium isolate E8202 plasmid 3 |
| DTU_2020_1007219_2_MG_RL_CPH_Sewage_646_S155_L004_contig=k141.778204_flag=0_coverage=22.0000_length=2895 | 2895 | vanHAX | Q05709 D-specific alpha-keto acid dehydrogenase  A0A0F6V063 D-alanine--D-alanine ligase  Q06241 D-alanyl-D-alanine dipeptidase | vanA  vanH  vanX | NC_008768.1 Enterococcus faecium plasmid pVEF1 |
| DTU_2020_1007222_1_MG_RL_CPH_Sewage_649_S13_L001_contig=k141.1124866_flag=1_coverage=20.9603_length=11234 | 11234 | vanHAX | A0A073K5I5 Resolvase  E0X5A7 VanS  Q05709 D-specific alpha-keto acid dehydrogenase  A0A0F6V063 D-alanine--D-alanine ligase  Q06241 D-alanyl-D-alanine dipeptidase  UPI00032D8ABD VanY-A/VanY-F/VanY-M family D-Ala-D-Ala carboxypeptidase  Q06242 Protein VanZ | vanA  vanH  vanR  vanS  vanX  vanY  vanZ-A | NC_016967.1 Enterococcus faecium plasmid pZB18 |
| DTU_2020_1007224_1_MG_RL_CPH_Sewage_651_S45_L001_contig=k141.1792110_flag=0_coverage=26.1828_length=9307 | 9307 | vanHAX | A0A073K5I5 Resolvase  E0X5A7 VanS  Q05709 D-specific alpha-keto acid dehydrogenase  A0A0F6V063 D-alanine--D-alanine ligase  Q06241 D-alanyl-D-alanine dipeptidase | vanA  vanH  vanR  vanS  vanX | NC_016967.1 Enterococcus faecium plasmid pZB18 |
| DTU_2020_1007226_1_MG_RL_CPH_Sewage_653_S47_L001_contig=k141.2164112_flag=0_coverage=9.3948_length=4027 | 4027 | vanHAX | Q05709 D-specific alpha-keto acid dehydrogenase  A0A0F6V063 D-alanine--D-alanine ligase  Q06241 D-alanyl-D-alanine dipeptidase | vanA  vanH  vanS  vanX | NC_008768.1 Enterococcus faecium plasmid pVEF1 |
| DTU_2020_1007227_1_MG_RL_CPH_Sewage_654_S71_L002_contig=k141.1235928_flag=0_coverage=34.0000_length=11174 | 11174 | vanHAX | UPI00032D8ABD VanY-A/VanY-F/VanY-M family D-Ala-D-Ala carboxypeptidase  Q06241 D-alanyl-D-alanine dipeptidase  A0A0F6V063 D-alanine--D-alanine ligase  Q05709 D-specific alpha-keto acid dehydrogenase  E0X5A7 VanS  Q9RMA4 Vanr protein  A0A073K5I5 Resolvase | vanA  vanH  vanR  vanS  vanX  vanY | NC_008768.1 Enterococcus faecium plasmid pVEF1 |
| DTU_2020_1007230_2_MG_RL_CPH_Sewage_662_S421_L004_contig=k141.61240_flag=1_coverage=4.0000_length=1663 | 1663 | vanHAX | A0A0F6V063 D-alanine--D-alanine ligase | vanA | NC_008768.1 Enterococcus faecium plasmid pVEF1 |
| DTU_2020_1007230_3_MG_RL_CPH_Sewage_662_S70_L002_contig=k141.468504_flag=1_coverage=5.0000_length=1784 | 1784 | vanHAX | Q05709 D-specific alpha-keto acid dehydrogenase | vanH | NC_008768.1 Enterococcus faecium plasmid pVEF1 |
| DTU_2020_1007230_3_MG_RL_CPH_Sewage_662_S70_L002_contig=k141.1151667_flag=1_coverage=5.0000_length=1232 | 1232 | vanHAX | A0A4U9PU57 D-alanine--D-lactate ligase  Q06241 D-alanyl-D-alanine dipeptidase | vanX | NC_008768.1 Enterococcus faecium plasmid pVEF1 |
| DTU_2020_1007233_1_MG_RL_CPH_Sewage_671_S49_L001_contig=k141.302991_flag=1_coverage=7.0000_length=6139 | 6139 | vanHAX | Q06242 Protein VanZ  UPI00032D8ABD VanY-A/VanY-F/VanY-M family D-Ala-D-Ala carboxypeptidase  Q06241 D-alanyl-D-alanine dipeptidase  A0A0F6V063 D-alanine--D-alanine ligase  Q05709 D-specific alpha-keto acid dehydrogenase  Q06240 Sensor protein VanS | vanA  vanH  vanS  vanX  vanY  vanZ-A | NC_008768.1 Enterococcus faecium plasmid pVEF1 |
| DTU_2020_1007236_2_MG_RL_CPH_Sewage_683_S66_L002_contig=k141.1204437_flag=1_coverage=5.0000_length=3810 | 3810 | vanHAX | E0X5A7 VanS  Q05709 D-specific alpha-keto acid dehydrogenase  A0A0F6V063 D-alanine--D-alanine ligase | vanA  vanH  vanS | NC_008768.1 Enterococcus faecium plasmid pVEF1 |
| DTU_2020_1007242_2_MG_RL_CPH_Sewage_695_S61_L002_contig=k141.935855_flag=1_coverage=9.0000_length=3065 | 3065 | vanHAX | Q05709 D-specific alpha-keto acid dehydrogenase  A0A0F6V063 D-alanine--D-alanine ligase | vanA  vanH | NC_008768.1 Enterococcus faecium plasmid pVEF1 |
| DTU_2020_1007242_2_MG_RL_CPH_Sewage_695_S61_L002_contig=k141.528624_flag=1_coverage=5.0000_length=2158 | 2158 | vanHAX | UPI00032D8ABD VanY-A/VanY-F/VanY-M family D-Ala-D-Ala carboxypeptidase | vanY | NC_008768.1 Enterococcus faecium plasmid pVEF1 |
| DTU_2020_1007245_1_MG_RL_CPH_Sewage_701_S84_L002_contig=k141.1329984_flag=1_coverage=7.0000_length=4067 | 4067 | vanHAX | A0A0F6V063 D-alanine--D-alanine ligase  Q06241 D-alanyl-D-alanine dipeptidase  UPI00032D8ABD VanY-A/VanY-F/VanY-M family D-Ala-D-Ala carboxypeptidase  Q06242 Protein VanZ | vanA  vanX  vanY  vanZ-A | NC_008768.1 Enterococcus faecium plasmid pVEF1 |
| DTU_2020_1007245_1_MG_RL_CPH_Sewage_701_S84_L002_contig=k141.1213296_flag=1_coverage=7.0000_length=3065 | 3065 | vanHAX | Q06240 Sensor protein VanS  Q05709 D-specific alpha-keto acid dehydrogenase | vanH  vanR  vanS | NC_008768.1 Enterococcus faecium plasmid pVEF1 |
| DTU_2020_1007261_1_MG_RL_CPH_Sewage_722_S120_L003_contig=k141.59379_flag=1_coverage=5.0000_length=3494 | 3494 | vanHAX | Q06241 D-alanyl-D-alanine dipeptidase  A0A0F6V063 D-alanine--D-alanine ligase  Q05709 D-specific alpha-keto acid dehydrogenase | vanA  vanH  vanX | NC_008768.1 Enterococcus faecium plasmid pVEF1 |
| DTU_2020_1007281_1_MG_RL_CPH_Sewage_1_677_S109_L003_contig=k141.437863_flag=0_coverage=34.9180_length=9259 | 9259 | vanHAX | A0A073K5I5 Resolvase  E0X5A7 VanS  Q05709 D-specific alpha-keto acid dehydrogenase  A0A0F6V063 D-alanine--D-alanine ligase  Q06241 D-alanyl-D-alanine dipeptidase | vanA  vanH  vanR  vanS  vanX | NZ_LR135346.1 Enterococcus faecium isolate E8202 plasmid 3 |
| DTU_2020_1007281_2_MG_RL_CPH_Sewage_1_677_S147_L004_contig=k141.106793_flag=1_coverage=4.0000_length=2459 | 2459 | vanHAX | A0A0F6V063 D-alanine--D-alanine ligase  Q05709 D-specific alpha-keto acid dehydrogenase | vanA  vanH | NC_008768.1 Enterococcus faecium plasmid pVEF1 |
| DTU_2020_1007282_1_MG_RL_CPH_Sewage_2_677_S110_L003_contig=k141.690041_flag=0_coverage=42.9164_length=16677 | 16677 | vanHAX | Q06242 Protein VanZ  UPI00032D8ABD VanY-A/VanY-F/VanY-M family D-Ala-D-Ala carboxypeptidase  Q06241 D-alanyl-D-alanine dipeptidase  A0A0F6V063 D-alanine--D-alanine ligase  Q05709 D-specific alpha-keto acid dehydrogenase  E0X5A7 VanS  A0A073K5I5 Resolvase  A0A377MLU7 UPF0637 family protein  A0A377MN13 Penicillin-binding protein transpeptidase | vanA  vanH  vanR  vanS  vanX  vanY  vanZ-A | Not a plasmid |
| DTU_2020_1007158_1_MG_RL_CPH_Sewage_518_S19_L001_contig=k141.1068717_flag=1_coverage=6.0000_length=1168 | 1168 | vanHBX |  |  | NZ_LR135359.1 Enterococcus faecium isolate E7948 plasmid 3 |
| DTU_2020_1007170_1_MG_RL_CPH_Sewage_571_S21_L001_contig=k141.480401_flag=1_coverage=7.0000_length=8167 | 8167 | vanHBX | Q47745 Sensor protein VanSB  A0A1A7T3C1 VanY protein, D-alanyl-D-alanine carboxypeptidase  Q47747 Vancomycin B-type resistance protein VanW  Q47748 D-specific alpha-keto acid dehydrogenase  Q47749 D-alanyl-D-alanine dipeptidase  Q714P2 Uncharacterized protein  B6ZHJ7 HTH_16 domain-containing protein  N2AXX1 Excisionase | vanA  vanH  vanS  vanW  vanX  vanY | NZ_LR135359.1 Enterococcus faecium isolate E7948 plasmid 3 |
| DTU_2020_1007176_1_MG_RL_CPH_Sewage_597_S23_L001_contig=k141.380984_flag=1_coverage=4.0000_length=1004 | 1004 | vanHBX |  |  | NZ_LR135359.1 Enterococcus faecium isolate E7948 plasmid 3 |
| DTU_2020_1007176_1_MG_RL_CPH_Sewage_597_S23_L001_contig=k141.283653_flag=1_coverage=4.0000_length=2137 | 2137 | vanHBX | Q47749 D-alanyl-D-alanine dipeptidase  Q714P2 Uncharacterized protein  B6ZHJ7 HTH_16 domain-containing protein |  | NC_011642.1 Enterococcus faecalis plasmid pMG2200 |
| DTU_2020_1007183_2_MG_RL_CPH_Sewage_605_S5_L001_contig=k141.1471296_flag=1_coverage=3.0000_length=1302 | 1302 | vanHBX |  |  | NZ_LR135359.1 Enterococcus faecium isolate E7948 plasmid 3 |
| DTU_2020_1007183_2_MG_RL_CPH_Sewage_605_S5_L001_contig=k141.911556_flag=1_coverage=3.0000_length=1818 | 1818 | vanHBX | Q47749 D-alanyl-D-alanine dipeptidase | vanX | NC_011642.1 Enterococcus faecalis plasmid pMG2200 |
| DTU_2020_1007189_1_MG_RL_CPH_Sewage_614_S73_L002_contig=k141.655769_flag=1_coverage=5.0000_length=1619 | 1619 | vanHBX |  | vanA | NC_011642.1 Enterococcus faecalis plasmid pMG2200 |
| DTU_2020_1007192_1_MG_RL_CPH_Sewage_617_S74_L002_contig=k141.1796091_flag=1_coverage=5.0000_length=1200 | 1200 | vanHBX |  |  | NC_011642.1 Enterococcus faecalis plasmid pMG2200 |
| DTU_2020_1007203_1_MG_RL_CPH_Sewage_628_S35_L001_contig=k141.654288_flag=1_coverage=5.0000_length=2340 | 2340 | vanHBX |  | vanA | NC_011642.1 Enterococcus faecalis plasmid pMG2200 |
| DTU_2020_1007209_1_MG_RL_CPH_Sewage_634_S81_L002_contig=k141.1734332_flag=1_coverage=2.0000_length=1999 | 1999 | vanHBX | Q47747 Vancomycin B-type resistance protein VanW | vanW | NZ_LR135359.1 Enterococcus faecium isolate E7948 plasmid 3 |
| DTU_2020_1007222_1_MG_RL_CPH_Sewage_649_S13_L001_contig=k141.1070558_flag=1_coverage=3.0000_length=1121 | 1121 | vanHBX |  |  | NZ_LR135359.1 Enterococcus faecium isolate E7948 plasmid 3 |
| DTU_2020_1007224_1_MG_RL_CPH_Sewage_651_S45_L001_contig=k141.316039_flag=1_coverage=5.0000_length=1008 | 1008 | vanHBX |  |  | NZ_LR135359.1 Enterococcus faecium isolate E7948 plasmid 3 |
| DTU_2020_1007224_1_MG_RL_CPH_Sewage_651_S45_L001_contig=k141.616910_flag=1_coverage=3.0000_length=1021 | 1021 | vanHBX |  |  | NC_011642.1 Enterococcus faecalis plasmid pMG2200 |
| DTU_2020_1007226_1_MG_RL_CPH_Sewage_653_S47_L001_contig=k141.304491_flag=1_coverage=5.0000_length=2633 | 2633 | vanHBX | A0A1A7T3C1 VanY protein, D-alanyl-D-alanine carboxypeptidase  Q47747 Vancomycin B-type resistance protein VanW | vanW  vanY | NZ_LR135359.1 Enterococcus faecium isolate E7948 plasmid 3 |
| DTU_2020_1007227_1_MG_RL_CPH_Sewage_654_S71_L002_contig=k141.924412_flag=1_coverage=5.0000_length=3439 | 3439 | vanHBX | N2AXX1 Excisionase  B6ZHJ7 HTH_16 domain-containing protein  Q714P2 Uncharacterized protein  Q47749 D-alanyl-D-alanine dipeptidase | vanX | NC_011642.1 Enterococcus faecalis plasmid pMG2200 |
| DTU_2020_1007227_1_MG_RL_CPH_Sewage_654_S71_L002_contig=k141.630108_flag=1_coverage=5.0000_length=3943 | 3943 | vanHBX | Q47748 D-specific alpha-keto acid dehydrogenase  Q47747 Vancomycin B-type resistance protein VanW  A0A1A7T3C1 VanY protein, D-alanyl-D-alanine carboxypeptidase | vanH  vanW  vanY | NZ_LR135359.1 Enterococcus faecium isolate E7948 plasmid 3 |
| DTU_2020_1007233_1_MG_RL_CPH_Sewage_671_S49_L001_contig=k141.85788_flag=1_coverage=4.0000_length=1720 | 1720 | vanHBX |  | vanA | NC_011642.1 Enterococcus faecalis plasmid pMG2200 |
| DTU_2020_1007236_2_MG_RL_CPH_Sewage_683_S66_L002_contig=k141.1084553_flag=1_coverage=5.0000_length=1239 | 1239 | vanHBX | Q47748 D-specific alpha-keto acid dehydrogenase |  | NZ_LR135359.1 Enterococcus faecium isolate E7948 plasmid 3 |
| DTU_2020_1007239_1_MG_RL_CPH_Sewage_689_S53_L001_contig=k141.153683_flag=0_coverage=11.9923_length=31543 | 31543 | vanHBX | Q9L903 Uncharacterized protein  A6P2J5 DUF5348 domain-containing protein  A0A3E2U5F7 PcfB family protein  A0A1C6B9R0 Conjugal transfer protein traG  A0A1E3A772 Maff2 family protein  Q9L8Z8 MunI-like protein  J6PBS5 Uncharacterized protein  Q9L8Z5 NLPC_P60 domain-containing protein  Q9L8Z4 Uncharacterized protein  J5DPS2 Uncharacterized protein  A0A0N7CCC4 Uncharacterized protein  J6C5Q4 Cys_rich_VLP domain-containing protein  A0A3E2U5G6 Endonuclease  Q9L8Y7 MobC domain-containing protein  Q9L8Y6 HTH cro/C1-type domain-containing protein  J7QJY3 Transposase  Q47744 Regulatory protein VanRB  Q47745 Sensor protein VanSB  A0A1A7T3C1 VanY protein, D-alanyl-D-alanine carboxypeptidase  Q47747 Vancomycin B-type resistance protein VanW  Q47748 D-specific alpha-keto acid dehydrogenase  Q47749 D-alanyl-D-alanine dipeptidase  Q714P2 Uncharacterized protein  B6ZHJ7 HTH_16 domain-containing protein  N2AXX1 Excisionase  J7Q5L1 Integrase | vanA  vanH  vanR  vanS  vanW  vanX  vanY | Not a plasmid |
| DTU_2020_1007242_2_MG_RL_CPH_Sewage_695_S61_L002_contig=k141.1155035_flag=1_coverage=6.0000_length=1607 | 1607 | vanHBX | Q47749 D-alanyl-D-alanine dipeptidase | vanX | NC_011642.1 Enterococcus faecalis plasmid pMG2200 |
| DTU_2020_1007245_1_MG_RL_CPH_Sewage_701_S84_L002_contig=k141.1786949_flag=1_coverage=5.0000_length=5604 | 5604 | vanHBX | J7Q5L1 Integrase  N2AXX1 Excisionase  B6ZHJ7 HTH_16 domain-containing protein  Q714P2 Uncharacterized protein  Q47749 D-alanyl-D-alanine dipeptidase | vanA  vanX | NC_011642.1 Enterococcus faecalis plasmid pMG2200 |
| DTU_2020_1007245_1_MG_RL_CPH_Sewage_701_S84_L002_contig=k141.429197_flag=1_coverage=5.0000_length=1496 | 1496 | vanHBX | Q47747 Vancomycin B-type resistance protein VanW | vanW | NC_011642.1 Enterococcus faecalis plasmid pMG2200 |
| DTU_2020_1007247_1_MG_RL_CPH_Sewage_707_S99_L003_contig=k141.1425620_flag=1_coverage=3.0000_length=1337 | 1337 | vanHBX |  |  | NZ_LR135359.1 Enterococcus faecium isolate E7948 plasmid 3 |
| DTU_2020_1007247_1_MG_RL_CPH_Sewage_707_S99_L003_contig=k141.1226461_flag=1_coverage=3.0000_length=2304 | 2304 | vanHBX | Q47749 D-alanyl-D-alanine dipeptidase  Q714P2 Uncharacterized protein  B6ZHJ7 HTH_16 domain-containing protein | vanX | NC_011642.1 Enterococcus faecalis plasmid pMG2200 |
| DTU_2020_1007249_1_MG_RL_CPH_Sewage_710_S135_L003_contig=k141.1369584_flag=1_coverage=5.0000_length=3796 | 3796 | vanHBX | Q47747 Vancomycin B-type resistance protein VanW  Q47748 D-specific alpha-keto acid dehydrogenase  Q47749 D-alanyl-D-alanine dipeptidase | vanA  vanH  vanW  vanX | NC_011642.1 Enterococcus faecalis plasmid pMG2200 |
| DTU_2020_1007252_1_MG_RL_CPH_Sewage_713_S113_L003_contig=k141.674571_flag=1_coverage=3.0000_length=2490 | 2490 | vanHBX | Q47749 D-alanyl-D-alanine dipeptidase | vanA  vanX | NC_011642.1 Enterococcus faecalis plasmid pMG2200 |
| DTU_2020_1007255_1_MG_RL_CPH_Sewage_716_S116_L003_contig=k141.849225_flag=1_coverage=4.0000_length=3242 | 3242 | vanHBX | Q47748 D-specific alpha-keto acid dehydrogenase | vanA  vanH | NC_011642.1 Enterococcus faecalis plasmid pMG2200 |
| DTU_2020_1007258_1_MG_RL_CPH_Sewage_719_S118_L003_contig=k141.1019652_flag=1_coverage=5.0000_length=3298 | 3298 | vanHBX | Q47749 D-alanyl-D-alanine dipeptidase  Q714P2 Uncharacterized protein  B6ZHJ7 HTH_16 domain-containing protein | vanA  vanX | NC_011642.1 Enterococcus faecalis plasmid pMG2200 |
| DTU_2020_1007261_1_MG_RL_CPH_Sewage_722_S120_L003_contig=k141.768631_flag=1_coverage=4.0000_length=1202 | 1202 | vanHBX | Q47749 D-alanyl-D-alanine dipeptidase | vanX | NC_011642.1 Enterococcus faecalis plasmid pMG2200 |
| DTU_2020_1007264_1_MG_RL_CPH_Sewage_725_S138_L003_contig=k141.769187_flag=1_coverage=5.0000_length=1194 | 1194 | vanHBX | Q47747 Vancomycin B-type resistance protein VanW | vanW | NC_011642.1 Enterococcus faecalis plasmid pMG2200 |
| DTU_2020_1007264_1_MG_RL_CPH_Sewage_725_S138_L003_contig=k141.1119708_flag=1_coverage=4.0000_length=2213 | 2213 | vanHBX |  | vanA | NC_011642.1 Enterococcus faecalis plasmid pMG2200 |
| DTU_2020_1007267_1_MG_RL_CPH_Sewage_731_S124_L003_contig=k141.414725_flag=1_coverage=4.0000_length=1145 | 1145 | vanHBX |  |  | NZ_LR135359.1 Enterococcus faecium isolate E7948 plasmid 3 |
| DTU_2020_1007272_1_MG_RL_CPH_Sewage_740_S127_L003_contig=k141.896025_flag=1_coverage=4.0000_length=1073 | 1073 | vanHBX | Q714P2 Uncharacterized protein |  | NZ_LR135359.1 Enterococcus faecium isolate E7948 plasmid 3 |
| DTU_2020_1007272_1_MG_RL_CPH_Sewage_740_S127_L003_contig=k141.654893_flag=1_coverage=5.0000_length=1268 | 1268 | vanHBX |  |  | NZ_LR135359.1 Enterococcus faecium isolate E7948 plasmid 3 |
| DTU_2020_1007276_1_MG_RL_CPH_Sewage_2_586_S106_L003_contig=k141.436631_flag=1_coverage=4.0000_length=1064 | 1064 | vanHBX | Q47749 D-alanyl-D-alanine dipeptidase | vanX | NC_011642.1 Enterococcus faecalis plasmid pMG2200 |
| DTU_2020_1007281_1_MG_RL_CPH_Sewage_1_677_S109_L003_contig=k141.1087927_flag=1_coverage=3.0000_length=3131 | 3131 | vanHBX | Q47748 D-specific alpha-keto acid dehydrogenase  Q47747 Vancomycin B-type resistance protein VanW | vanH  vanW | NZ_LR135359.1 Enterococcus faecium isolate E7948 plasmid 3 |
| DTU_2020_1007281_1_MG_RL_CPH_Sewage_1_677_S109_L003_contig=k141.1109679_flag=1_coverage=4.0000_length=1432 | 1432 | vanHBX | Q714P2 Uncharacterized protein |  | NZ_LR135359.1 Enterococcus faecium isolate E7948 plasmid 3 |
| DTU_2020_1007282_1_MG_RL_CPH_Sewage_2_677_S110_L003_contig=k141.1515673_flag=1_coverage=3.0000_length=1332 | 1332 | vanHBX |  |  | NC_011642.1 Enterococcus faecalis plasmid pMG2200 |
